# Asymmetric Anticipatory Emotions and Economic Preferences: Dread, Savoring, Risk, and Time

**DOI:** 10.1111/cogs.70160

**Published:** 2026-01-20

**Authors:** Chris Dawson, Samuel G. B. Johnson

**Affiliations:** ^1^ School of Management University of Bath; ^2^ Department of Psychology University of Waterloo; ^3^ Department of Psychology University of Warwick

**Keywords:** Anticipatory emotions, Intertemporal choice, Risky choice, Behavioral economics

## Abstract

We are often preoccupied with the future, experiencing dread at the thought of future misery and savoring the thought of future pleasure. Prior lab studies have found that these anticipatory emotions influence decision‐making. In this article, using economic survey data to estimate individual differences in anticipatory emotions, we find that the tendency to feel displeasure from anticipating future losses outweighs the pleasure from anticipating equal gains. We then relate asymmetries in anticipatory emotions to key economic preferences, finding that people with more strongly asymmetric anticipatory emotions are more risk‐avoidant (because they obtain more disutility from contemplating downside risk) and more impatient (because they want to minimize the time spent contemplating risks). We conclude by considering how asymmetries in anticipatory emotions may be linked to a range of intertemporal and risky choice phenomena. Overall, our framework explains why risk‐avoidance and impatience are linked, and we provide suggestive evidence for this explanation.

## 1 Introduction

Many decisions, big and small, involve both risk and delay—starting a business, choosing a spouse, even submitting a journal article. Such decisions are often accompanied by powerful anticipatory emotions, such as dread and savoring, felt while waiting for the uncertainty to be resolved. These decisions are much like roulette: Our decision to bet is based not only on the likelihood and magnitude of the potential gains and losses but on how well we imagine we can endure the feeling of dread—or enjoy the feeling of savoring—as the wheel spins. If decision‐makers account for anticipatory emotions when making risky delayed choices, their influence could be considerable: They are experienced continuously from the time of the decision until the event occurs, and anticipating an unpleasant event can even elicit stronger psychological responses than experiencing the event itself (Berns et al., 2006; Lazarus, 1966).

Given their potentially profound influence on decision‐making, this article proposes a model of how anticipatory emotions influence risk and time preferences, along with suggestive evidence for this model. First, we find that negative anticipation (*dread*) is more powerful than positive anticipation (*savoring*). Second, this asymmetry is associated with both risk‐avoidance and impatience. To the extent dread is expected to outweigh savoring, this makes a gamble less attractive. And if the bet is placed, the gambler pleads for the roulette wheel to stop, to resolve the dread‐inducing uncertainty. Thus, asymmetric anticipatory emotions provide the missing psychological link between risk and time preferences.

**Fig 1:**
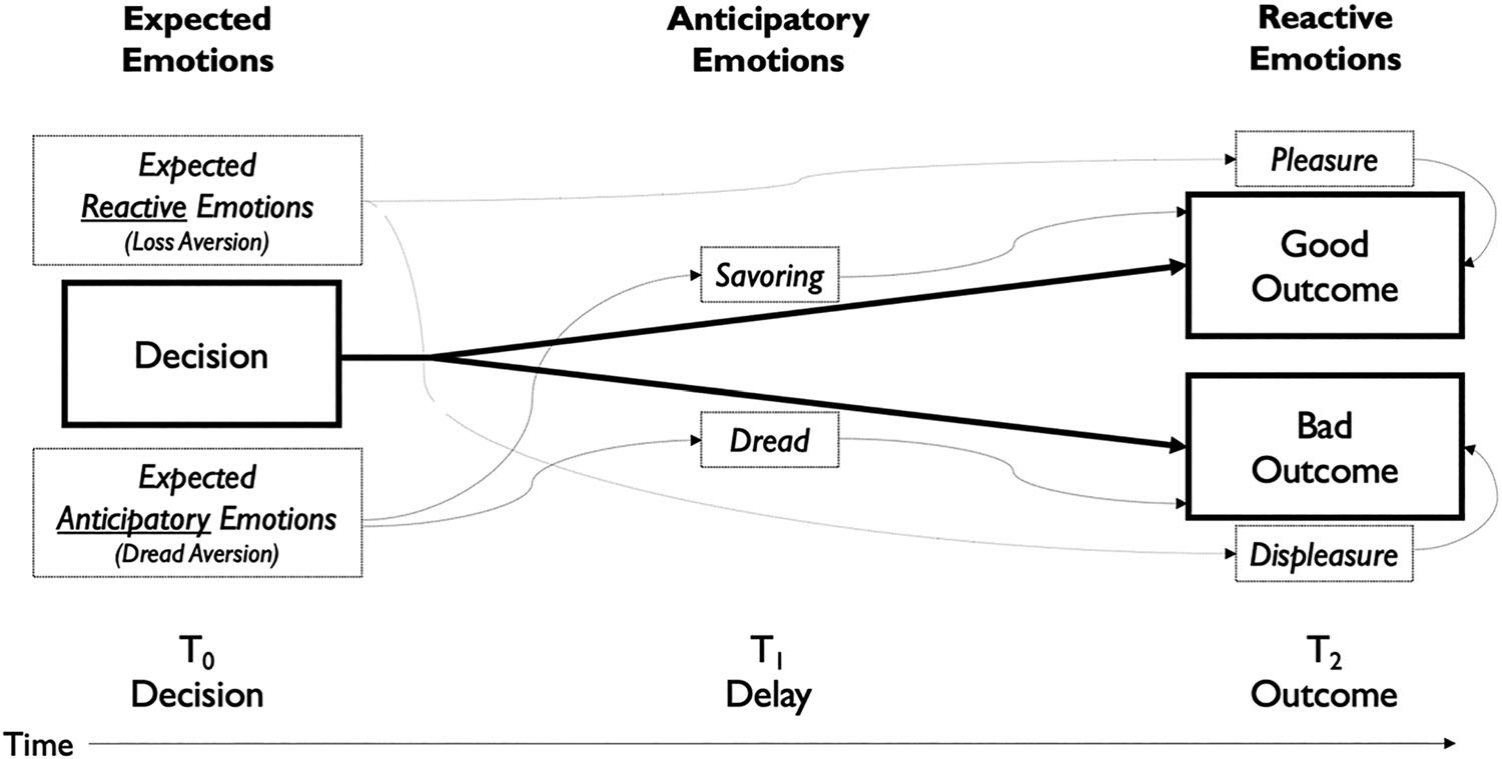
Distinctions among expected, anticipatory, and reactive emotions along the timecourse of decision‐making.

We distinguish three kinds of emotions that influence decision‐making based on their temporal profile (Baumgartner, Pieters, & Bagozzi, 2008; Loewenstein, Weber, Hsee, & Welch, 2001). Consider a choice made at time $T_{0}$ that will deliver a good or bad outcome at $T_{2}$. First, we feel *reactive* emotions in response to resolved outcomes, such as the (dis)pleasure of experienced gains and losses. In Fig. 1, reactive emotions occur at $T_{2}$ in response to outcomes (e.g., money gained or lost at roulette). Second, we feel *anticipatory* emotions before an outcome, generated from mental imagery of the decision's expected consequences—*savoring* or *dread* in anticipation of imagined good or bad $T_{2}$ outcomes (Loewenstein, 1987). Anticipatory emotions occur at $T_{1}$ in Fig. 1 (e.g., while the roulette wheel is spinning). Third, we forecast our future emotions at $T_{0}$ (e.g., when placing bets); these are *expected emotions*. Since reactive and anticipatory emotions occur after the decision is made, these emotions influence choice only through how they are *expected* at $T_{0}$ (Mellers, Schwartz, & Ritov, 1999).

*Expected reactive emotions* manifest in the avoidance, at $T_{0}$, of options expected to lead to negative reactive emotions at $T_{2}$, such as regret or disappointment (Loomes & Sugden, 1982). Famously, people are *loss averse* (Kahneman & Tversky, 1979)—they assume (at $T_{0}$) that the displeasure of losses is greater than the pleasure of gains (at $T_{2}$). Interestingly, loss aversion may be partially an affective‐forecasting error: *forecasts* of loss aversion are large, whereas the *realized* emotional impact of losses and gains is more symmetric (Kermer, Driver‐Linn, Wilson, & Gilbert, 2006). Yet this forecast, even if erroneous, matters greatly for decision‐making: At the time a decision is made ($T_{0}$), we know only how we *expect* it will make us feel.

*Expected anticipatory emotions* are known to influence intertemporal choice. Whereas most behavioral models aim to explain why people *undervalue* future outcomes (Loewenstein & Prelec, 1992), at other times, people value the future *too highly*. Loewenstein (1987) showed that discount rates can be *negative* when future sources of (dis)utility are particularly vivid. For example, Loewenstein's participants were willing to pay more to receive a kiss from a movie star in 3 days than 1 day and more to avoid an electric shock in 1 year than 3 days. Loewenstein assumed that this occurs because people gain or lose utility not only from *consuming* these events (associated with reactive emotions) but also from *imagining* them (associated with anticipatory emotions). Whereas consumption utility will only be attained in the future, and hence is steeply discounted, (dis)utility from savoring or dread is experienced continuously from the time of the decision until the event. Thus, experiencing the shock sooner avoids disutility from dread that more than offsets the discounting of its consumption disutility. Subsequent studies confirm this basic picture (Berns et al., 2006; Harris, 2011; Story et al., 2013).

However, dread and savoring are not equally powerful (Molouki, Hardisty, & Caruso, 2019). The disutility from imagining negative events is greater than the utility of imagining comparable positive events for two reasons (Table 1). First, since people are loss averse, anticipatory emotions at $T_{1}$ should reflect the greater disutility of bad outcomes at $T_{2}$. Just as dread would be greater for a $200 than for a $100 loss, dread for a $100 loss (psychologically valued at $200 due to loss aversion) would be greater than savoring for a $100 gain (valued at $100). Second, dread itself could be a more powerful emotion than savoring—an asymmetry we dub *dread aversion* (Dawson & de Meza, 2018). Imagining a positive outcome is emotionally mixed, whereas imagining a negative outcome is purely negative (Nowlis, Mandel, & McCabe, 2004). Whereas we dread an upcoming electric shock, we experience both savoring *and* impatience over an upcoming vacation. For example, most people prefer to resolve lotteries *sooner* as the magnitude of the payoff increases, suggesting that savoring competes (and sometimes loses) to other emotions such as impatience (Ganguly & Tasoff, 2017). Hence, lab studies tend to find little evidence of savoring even as they find substantial levels of dread (Hardisty & Weber, 2020).

**Table 1:** Loss aversion and dread aversion as channels for economic preferences

	Effect	Loss Aversion	Dread Aversion
Reactive emotions *(Pleasure vs. Displeasure)*	Expected emotions	Displeasure after losses will be greater than pleasure after comparable gains	–
	Risk preferences	*Increases risk‐avoidance*: Eliminating downside risk more valuable with more intense displeasure over losses	–
	Time preferences	–	–
Anticipatory emotions *(Dread vs. Savoring)*	Expected emotions	Dread is more powerful than savoring because the *object* of dread is future displeasure	Dread is itself a more powerful emotion than savoring
	Risk preferences	*Increases risk‐avoidance*: Eliminating downside risk more valuable when dread pertains to more intensely displeasing losses	*Increases risk‐avoidance*: Eliminating downside risk more valuable with more intense dread over potential loss
	Time preferences	*Increases impatience*: Eliminating delay more valuable when dread is accrued over more intensely displeasing losses	*Increases impatience*: Eliminating delay more valuable with more intense dread accruing over the delay

In this article, we argue that asymmetric anticipatory emotions explain a fundamental puzzle about economic preferences: why risk and time preferences are related. Standard models of risk and time preferences treat these as separate dimensions, yet several lines of thinking suggest they are not independent. First, theorists have noted that the future is inherently uncertain, suggesting that risk attitudes are likely to affect intertemporal choices (Bixter & Luhmann, 2015; Dasgupta & Maskin, 2005; Epper & Fehr‐Duda, 2024). Second, risk and time preferences are subject to some of the same anomalies in experiments (Epper & Fehr‐Duda, 2024) and time preferences shift when risk is introduced, undermining the idea that these preferences are independent (Anderson & Stafford, 2009; Hardisty & Pfeffer, 2017). Third, studies of preference elicitation have found that eliciting risk and time preferences simultaneously leads to more plausible discount rates because of the link between impatience and risk‐avoidance (Andersen, Harrison, Lau, & Rutström, 2008). Finally, there is direct evidence that individuals higher in risk‐avoidance are also more impatient (e.g., Anderhub, Güth, Gneezy, & Sonsino, 2003; Dean & Ortoleva, 2019), although some discrepant studies exist (e.g., Sutter, Kocher, Glätzle‐Rützler, & Trautmann, 2013; Tasoff & Zhang, 2022).

However, little empirical work has examined *why* risk and time preferences are intertwined. We propose that asymmetries in anticipatory emotions provide this missing link (Table 1). In the real world, many decisions are both risky *and* the payoffs are delayed. For example, when we decide what job to take, where to live, or who to marry, we will not know the consequences for months or years; likewise, many (if not most) business decisions are both uncertain and delayed. When decisions involve both risk and delay, anticipatory emotions play a powerful role, as we are licensed to imagine both good and bad outcomes until the outcome is resolved (Golman, Gurney, & Loewenstein, 2021). To the extent that anticipatory emotions are asymmetric, disutility from imagining the bad outcomes outweighs the utility from imagining the good outcomes. This implies a link between risk‐avoidance and impatience. Those who experience more intense dread will experience more disutility from future uncertainty, and this disutility will be proportional to the time delay until the uncertainty is resolved. If people correctly forecast these anticipatory emotions, they will be more risk‐avoidant and more impatient: more risk‐avoidant because avoiding risks minimizes disutility from dread and more impatient because resolving uncertainty faster minimizes the time spent experiencing disutility from dread.

Aspects of these predictions have accrued support. On the savoring side, although most people prefer to find out the outcome of a lottery immediately, a minority—who are especially optimistic about the outcome and are more prone to experiencing the “thrill” of waiting—prefer delayed resolution (Kocher, Krawczyk, & van Winden, 2014). On the dread side, using emotion regulation strategies—such as reappraising emotions to manage their impact on decision‐making—decreases risk‐avoidance (Heilman, Crisan, Houser, Miclea, & Miu, 2010), implicating a direct causal link between emotional reasoning and risky choice. This fits the broader association between neuroticism (emotional instability) and risk‐avoidance (Borghans, Golsteyn, Heckman, & Meijers, 2009).

However, the primary obstacle to amassing large‐scale evidence relating anticipatory emotions to economic preferences is measuring anticipatory emotions in economic survey data. Here, we develop a novel approach to jointly estimating anticipatory savoring and dread across a nationally representative sample from the United Kingdom. To our knowledge, this study is the first to elicit individual levels of dread and savoring and relate them to risk and time preferences. Since both loss aversion and dread aversion are expected to contribute to asymmetries in anticipatory emotions, we also estimate individual levels of loss aversion to tease apart these effects.

## 2 Theor**y**

### 2.1 Model

We model a decision made at $T_{0}$ whether to accept a gamble that will be resolved after an interval *T*. We follow prior models (Caplin & Leahy, 2001; Loewenstein, 1987), in assuming that the agent aims to maximize expected *psychological* utility, $U=U_{C}+U_{A}$, where $U_{C}$ is the expected *consumption* utility—expected pleasure or displeasure at the actual outcome at time $t=T_{0}+T$—and $U_{A}$ is expected *anticipatory utility—*expected dread or savoring to be experienced during the interval $\left[T_{0},T_{0}+T\right]$. For example, if the agent is deciding whether to take a coin toss gamble where heads pays +$100 and tails costs –$100, we assume this will depend on the agent's forecasts about both the reactive emotions associated with each potential outcome and the anticipatory emotions associated with uncertainty while waiting for the resolution.

Consider a gamble offering the monetary payoffs ($x_{1}$,…, $x_{n}$) with probabilities ($p_{1}$,…, $p_{n}$) that is resolved after an interval *T*. We make the standard assumption that expected consumption utility $U_{C}$ is given by the psychological value of each payoff, $v_{C}\left(x_{i}\right)$, weighted by a function of its probability, $\;\pi_{C}\left(p_{i}\right)$, and discounted by $\delta_{C}\left(T\right)$:

(1) $$U_{C}=\sum_{i}v_{C}\left(x_{i}\right)\pi_{C}\left(p_{i}\right)\delta_{C}\left(T\right).$$

We assume that $v_{C}\left(x_{i}\right)$ is increasing, that $v_{C}\;\left(0\right)=0$, and, following prospect theory (Kahneman & Tversky, 1979), that people are loss averse: if $x_{i}<0$, then $v_{C}\;\left(x_{i}\right)=-\lambda v_{C}\left(-x_{i}\right)$ for loss aversion parameter λ>1.

We now add $U_{A}$, the expected utility from anticipation:

(2) $$U_{A}=\int_{0}^{T}\sum_{i}\mu \left(x_{i},t\right)\pi_{A}\left(p_{i}\right)\delta_{A}\left(t\right)dt.$$

We allow the weighting function $\pi_{A}\left(p_{i}\right)$ and discounting function $\delta_{A}\left(t\right)$ to potentially differ for consumption and anticipatory utility, and we introduce an imagination function $\mu (x_{i},t)$, which serves an analogous role to the value function for consumption utility by giving the momentary value of the anticipatory emotions associated with outcome $\;x_{i}$ at time t. Eq. (2) simply adds up the expected anticipatory utilities for each outcome *i* and integrates from 0 to T since anticipatory (dis)utility is accumulated continuously until the outcome.

To implement the notion of dread aversion, we define the imagination function $\mu (x_{i},t)$ as follows:

(3) $$\mu \left(x_{i},t\right)=\left\{\begin{array}{c} \alpha v_{A}\left(x_{i}\right)a\left(t\right)\;\mathrm{for}\;\;x_{i}\geq 0\\ -\sigma \alpha v_{A}\left(-x_{i}\right)a\left(t\right)\;\mathrm{for}\;x_{i}<0 \end{array}\right..$$

In Eq. (3), α≥0 is a vividness parameter, capturing individual differences in the vividness (overall level) of anticipatory emotions; σ>1 is a dread aversion parameter, reflecting the extent to which dread is a more powerful emotion than savoring; $v_{A}\left(x_{i}\right)$ is a value function satisfying the same assumptions mentioned above for $v_{C}\left(x_{i}\right)$, but potentially differing in functional form; and a(t) is an attention function taking values in [0,1], which allows the overall level of anticipatory utility to vary over time. For example, anticipatory emotions could be especially powerful immediately after a decision is made and immediately before the outcome is revealed (Baucells & Bellezza, 2017).

This model reflects both rationales for asymmetric anticipatory emotions. First, even if we set σ=1, then loss aversion (λ>1) still implies that $|\mu (-x_{i},t)\left|>\right|\mu (x_{i},t)|$ for positive $x_{i}$ since $|v_{A}\left(-x_{i}\right)\left|>\right|v_{A}\left(x_{i}\right)|$ and $\mu \left(x_{i},t\right)\propto \;v_{A}\left(x_{i}\right)$. That is, contemplating losses is more painful than contemplating gains is pleasurable in part because the subjective value of losses is exaggerated due to loss aversion. This rationale does not assume that dread itself is a stronger emotion than savoring but instead that the *amount* of dread over losing $x_{i}$ would be greater than the amount of savoring over gaining $x_{i}$ since loss aversion says that the disutility of a $x_{i}$ loss is greater than the utility of a $x_{i}$ gain. Second, we *additionally* allow negative anticipatory emotions to be more powerful than positive ones (σ>1) because anticipating negative events is purely aversive, whereas anticipating positive events mixes positive (savoring) and negative (impatience) emotions (Hardisty & Weber, 2020; Nowlis et al., 2004).

### 2.2 Graphical representation

Let us examine graphically how incorporating anticipatory utility changes total utility as a function of $x_{i}$ and t. To simplify calculations and isolate the effects of anticipatory utility, we assume for the purpose of visual depiction (but *not* for proofs presented below) that: (i) probability weighting is linear for both consumption and anticipation [$\pi_{C}\;\left(p_{i}\right)=\pi_{A}\;\left(p_{i}\right)=p_{i}\;$]; (ii) there is no discounting [$\delta_{C}\;\left(t\right)=\delta_{A}\;\left(t\right)=1$]; and (iii) attention is constant, a(t)=1, so that imagination is a function only of the value of the outcome, vividness of imagination, and dread aversion [$\mu \;\left(x_{i},t\right)=\alpha v_{A}\;\left(x_{i}\right)$ for $x_{i}\geq 0$ and $\mu \;\left(x_{i},t\right)=-\sigma \alpha v_{A}\left(-x_{i}\right)$ for $x_{i}<0$]. This allows us to write total utility as

(4) $$U=p_{i}\left[v_{C}\left(x_{i}\right)+\int_{0}^{T}\sum_{i}\mu \left(x_{i},t\right)dt\right].$$

Since $p_{i}$ is constant with respect to $x_{i}$ and t, we ignore this parameter here and plot $v_{C}\left(x_{i}\right)+\int_{0}^{T}\sum_{i}\mu \left(x_{i},t\right)dt$ as a function of $x_{i}$ at different values of t. For these plots, we use the standard prospect theory value function for $v_{C}\left(x_{i}\right)$ and a linear value function for $v_{A}\left(x_{i}\right)$ (i.e., $v_{A}\;\left(x_{i}\right)=x_{i}\;$), to ensure that our analysis does not depend on the S‐shaped prospect theory value function for anticipation. We set the vividness parameter α=0.05.

**Fig 2:**
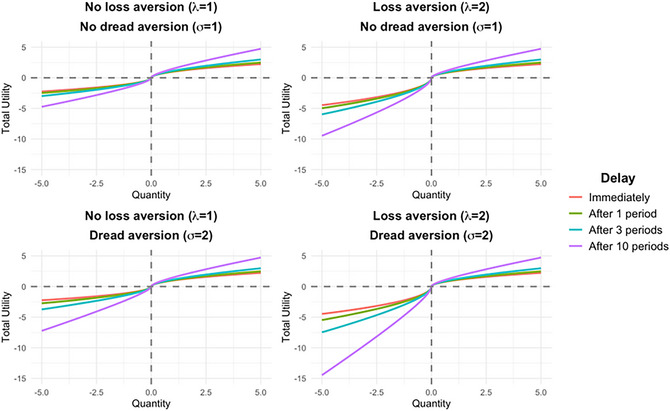
Total utility (consumption and anticipation) at four delays under different assumptions about loss aversion and dread aversion.

Each plot in Fig. 2 depicts total utility (consumption and anticipation) at four different delays—immediately (red line), after one period (green), after three periods (blue), and after 10 periods (purple). The four panels depict different combinations of loss aversion and dread aversion.

The top left plot shows total utility with neither loss aversion nor dread aversion, which is symmetric around 0 as losses and gains are weighted equally. Importantly, both the utility from gains and disutility from losses are greater at longer delays because there is more time for anticipatory (dis)utility to accumulate at longer delays. The red line therefore represents the standard prospect theory value function (minus loss aversion), and the other lines show how the utility from both gains and losses becomes more extreme with delay. For mixed gambles, however, these more extreme utility values would not produce a change in expected utility and would therefore not lead to risk avoidance.

The top right plot introduces loss aversion. The immediate (red) line is now the familiar kinked S‐shape of prospect theory. Losses become more and more unpleasant at longer delays because of dread, whereas the corresponding and opposite effect for gains due to savoring is smaller. The difference between the immediate line and 10 period lines reflects anticipatory (dis)utility accrued over 10 periods, and this difference is larger for losses than for gains (unlike the upper left plot without loss aversion). This represents the first way that anticipatory emotions can be asymmetrical: anticipating losses adds more disutility than anticipating gains adds utility because the object of anticipation is weighed more heavily (loss aversion). This exacerbates the risk‐avoidance‐inducing effect of loss aversion at greater delays. It also creates an incentive to shorten delays, so that utility can shift from the 10 period (purple) line toward the immediate (red) line.

The bottom left plot adds dread aversion instead of loss aversion. Whereas the immediate (red) is the same as in the top left plot (without loss or dread aversion), the 10 period (purple) line diverges more for losses than for gains, as in the top right plot (with loss aversion). This represents the second way that anticipatory emotions can be asymmetrical: Even if the object of anticipation is the same (i.e., no loss aversion), the experience of negative anticipation can itself be a more powerful emotion (dread aversion). As with loss aversion, dread aversion can lead to risk avoidance for mixed gambles. Unlike loss aversion, however, this effect occurs only when outcomes are delayed—not for immediate gambles—since the immediate (red) line is symmetric in the absence of loss aversion. Both loss aversion and dread aversion create similar incentives to shorten delays, moving from the more kinked 10 period line toward the less kinked immediate line.

Finally, the bottom right plot includes both loss aversion and dread aversion, showing that both channels combined can have an even larger effect on total utility and therefore on risk and time preferences.

### 2.3 Risk preferences

Loss aversion and dread aversion both straightforwardly increase risk‐avoidance in our model. As both λ and σ increase, the utility of the gamble decreases at any given T>0. Specifically, when at least one of the $x_{i}$’s is negative, this implies that U decreases as a function of λ and σ since λ and σ only appear in the negative terms, $x_{i}<0$, of the expressions for both $U_{A}$ and $U_{C}$ in Eqs. (1) and (2). Intuitively, to the extent that dread outweighs savoring, risky (mixed) gambles are unappealing because the dread of the possible downside increasingly outweighs the savoring of the possible upside (i.e., the difference between the negative and positive sides of the plots in Fig. 2).

To illustrate this, let us return to the coin flip example, with equal chances of gaining or losing $100, with the gamble resolved after three periods. To study risk preferences, we calculate the *delayed certainty equivalent* of this gamble. These isolate risk preferences (over‐and‐above time preferences) by specifying the sure‐thing payoff—after the same three‐period delay—that would make one indifferent between that sure‐thing payoff and the gamble. We make the same assumptions for these calculations as in Fig. 2, except that we now assume the value functions for both consumption and anticipation are linear, with the exception of loss aversion [$v_{C}\;\left(x_{i}\right)=v_{A}\;\left(x_{i}\right)=x_{i}$ for $x_{i}\geq 0$ and $v_{C}\;\left(x_{i}\right)=v_{A}\;\left(x_{i}\right)=\lambda x_{i}\;$ for $x_{i}<0$]. Importantly, these are simplifying assumptions for the numeric example and are *not* assumptions required by our model. Our theoretical results depend only on the much more general assumptions described in Section 2.1.

In Table 2, we calculate the delayed certainty equivalent while varying three parameters. We set α to either 0 or 0.05 (to study the effect of anticipatory utility), λ to either 1 or 2 (to study the effect of loss aversion), and σ to either 1 or 2 (to study the effect of dread aversion). More negative values reflect greater risk‐avoidance since one is willing to pay more to avoid the gamble.

**Table 2:** Examples of risk and time preferences for time‐delayed gambles

Assumptions			Utility				Preferences	
α	λ	σ	$U_{C}\left(gain\right)$	$U_{C}\left(loss\right)$	$U_{A}\left(gain\right)$	$U_{A}\left(loss\right)$	Delayed Certainty Equivalent	Present Gamble Equivalent
0	1	1	50	−50	0	0	$0	$100
0	2	1	50	−100	0	0	−$25	$100
0	1	3	50	−50	0	0	$0	$100
0	2	3	50	−100	0	0	−$25	$100
0.05	1	1	50	−50	7.5	−7.5	$0	$100
0.05	2	1	50	−100	7.5	−15	−$28.75	$85
0.05	1	2	50	−50	7.5	−15	−$7.50	$85
0.05	2	2	50	−100	7.5	−30	−$36.25	$55

*Note*. Entries relate to a gamble to be resolved after three periods, with a 50% chance of gaining $100 and 50% chance of losing $100. This gamble is modeled under the assumptions that (i) anticipatory utility is either absent or accrues at 0.05 times consumption utility per period (α of 0 or 0.05); (ii) loss aversion is either absent or losses are twice as powerful as gains (λ of 1 or 2); and (iii) dread aversion is either absent or dread is twice as powerful as savoring (σ of 1 or 2). Consumption utility ($U_{C}$) and anticipatory utility ($U_{A}$) are calculated separately for the $100 gain and $100 loss after the three periods; see main text for the assumptions underlying these calculations (importantly, there is no discounting). The *delayed certainty equivalent* is the sure‐thing payoff after three periods, which would make the decision‐maker indifferent between that payoff and the gamble; thus, negative values reflect risk‐avoidance and positive values reflect risk‐seeking. The *present gamble equivalent* is the value of the upside in an immediate gamble with a $100 downside, which would make the decision‐maker equivalent between that gamble and the delayed gamble; thus, values less than $100 indicate greater impatience.

When α=0, there is no anticipatory utility. Since dread aversion σ is only reflected through anticipatory utility, it has no effect under these conditions (Row 1 vs. 3). However, loss aversion λ has the usual effect of increasing risk‐avoidance, with lower delayed certainty equivalents when λ is higher (Row 1 vs. 2). Introducing anticipatory utility by setting α=0.05 does not affect risk preferences if gains and losses are weighted symmetrically (λ=σ=1; Row 1 vs. 5). However, asymmetries in gain/loss weighting affect anticipatory utility and hence risk‐avoidance. Greater loss aversion λ results in more negative consumption utility, and through this effect more anticipatory disutility (Row 5 vs. 6). Greater dread aversion σ has no effect on consumption utility but a multiplicative effect with loss aversion on anticipatory disutility (Rows 5–8). As a result, increasing both loss aversion λ (through both consumption and anticipatory utility) and dread aversion σ (through anticipatory utility only) lead the gamble to appear less attractive, making a decision‐maker willing to pay increasingly more to avoid the risk. In this example, the effect of loss aversion on risk preferences is larger than the effect of dread aversion because loss aversion affects both consumption and anticipatory utility, whereas dread aversion affects only anticipatory utility, and both contribute to risk preferences. Finally, it is worth noting that higher values of α lead to both more anticipatory utility *and* disutility. Intuitively, this is because people with more vivid emotional imaginations tend to experience both dread and savoring more strongly.

### 2.4 Time preferences

In the case of impatience, the argument is slightly more complex. The model implies that utility declines more steeply with time for higher values of σ due to more negative anticipatory utility. To see this, consider the derivative of $U=U_{A}+U_{C}$ (i.e., the sum of Eqs. 1 and 2) with respect to time:

(5)

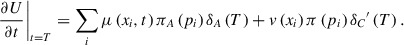




Negative values of $\frac{\partial U}{\partial t}{|}_{t=T}$ corresponds to greater impatience in the sense of preferring outcomes to occur sooner. The σ parameter only appears through $\mu (x_{i},t)$ and only for terms where $x_{i}<0$. These terms are all negative: in Eq. (3), $\mu (x_{i},t)$ is proportional to $v_{A}\left(x_{i}\right)$, which is an increasing function with $v_{A}\;\left(0\right)=0$, and no other constant or function in $\mu (x_{i},t)$ can take negative values. Therefore, $\frac{\partial U}{\partial t}{|}_{t=T}$ becomes more negative as σ increases, showing that dread aversion implies greater impatience. Intuitively, this happens because greater σ corresponds to higher levels of anticipatory disutility, which accumulates as the interval increases in length T.

This should be contrasted to λ, where the effect is ambiguous. Higher values of λ may or may not increase impatience since λ appears in the terms for both anticipatory utility (where it unambiguously increases impatience for the reasons described in the above paragraph) and consumption utility (where its effect depends on the sign of ${\delta_{C}}^{\prime }\left(T\right)$). Since discounting functions are typically assumed to be decreasing in T—that is, one places less value on future outcomes—this derivative is usually negative, producing *lower* impatience through the consumption utility channel. Intuitively, the more one is loss averse, the less consumption disutility one expects to experience as the potential loss is delayed further into the future. Since the effects of consumption and anticipatory utility move in opposite directions, λ may or may not produce impatience in the model.

To illustrate the effects of λ and σ on time preferences, let us return once again to the coin flip above. For each setting of α, λ, and σ and using the same assumptions as above, we now calculate *present gamble equivalents* in the rightmost column of Table 2. These isolate time preferences (over‐and‐above risk preferences) by specifying the upside of the gamble—resolved and paid out at the present time, with the same probabilities and same downside as the delayed gamble—that would make one indifferent between that present gamble and the delayed gamble. For instance, in our running example, a present gamble equivalent of $50 would mean that one would be indifferent between (i) a coin‐flip, resolved immediately, with a $50 upside and $100 downside, and (ii) a coin‐flip, resolved after three periods, with a $100 upside and $100 downside. Thus, lower values reflect greater impatience since one is willing to accept a smaller potential gain to resolve the gamble sooner rather than later.

Loss aversion and dread aversion have no effect on time preferences when there is no anticipatory utility (α=0; Rows 1–4) due to our simplifying assumption of no time discounting. However, anticipatory (dis)utility affects time preferences through a separate channel independent of discounting. With anticipatory utility (α=0.05), there is still no effect on time preferences with symmetric weighting of gains and losses (λ=σ=1; Row 1 vs. 5). Yet, both loss aversion and dread aversion increase impatience via anticipatory utility (Rows 5–8). This happens because greater attention to losses in anticipation (through both λ and σ) increases the attractiveness of resolving the gamble sooner.

### 2.5 Simulations

To supplement this formal argument, we conducted a series of simulations in which we use implementations of the formal framework above to examine the effects of α, λ, and σ on risk and time preferences, as well as the interactions between α∗λ and α∗σ. Specifically, we simulate a decision‐maker who maximizes utility as given by Eqs. (1)–(3) and who faces a mixed gamble with delayed resolution. We calculate the future certainty equivalent (to quantify risk avoidance) and present gamble equivalent (to quantify impatience) while varying α, λ, and σ across five levels each, calculating regression coefficients for α, λ, σ, α∗λ, and α∗σ. We repeat this procedure for several sets of choice characteristics—varying the delay, the ratio of the expected gain to expected loss, and the probability of gain versus loss—and a variety of different assumptions about the value, weighting, and discounting functions—which we allowed to vary for consumption and anticipatory utility—and the attention function.

We report detailed results of these simulations in Appendix A of the Supporting Information. The main finding—albeit not a surprising one, in light of the formal arguments in Sections 2.3 and 2.4—is that out of the 234 combinations of choice characteristics and functional forms tested, greater λ and σ lead to greater risk avoidance in every simulation, and greater σ leads to greater impatience in every simulation. Consistent with the ambiguous effects of λ on time preferences from the formal argument above, this effect was somewhat inconsistent and did not manifest in simulations with high rates of consumption utility discounting. The effects of α on both risk and time preferences also varied across simulations in ways that make intuitive and theoretical sense (e.g., α is more likely to produce risk‐seeking when the expected value of the potential gain is higher relative to the potential loss since this creates more opportunity for savoring). Finally, in all simulations, the interaction effects for α∗λ and α∗σ were negative for both risk preferences and time preferences, indicating that more powerful anticipatory emotions exacerbated the risk‐aversion‐ and impatience‐inducing effects of loss aversion and dread aversion.

### 2.6 Summary

Overall, the model formalizes three insights. First, anticipatory utility is likely to be asymmetric for dread versus savoring—through both a loss aversion and dread aversion mechanism—as explained informally in Section 1. Second, asymmetric experiences of dread and savoring can lead to greater risk‐avoidance through both loss aversion (because the object of dread is more potent) and dread aversion (because the emotion of dread itself is more potent); taking on downside risks introduces dread, and this experience decreases the emotional utility of risk‐taking. Third, these asymmetries also lead to greater impatience, once again due to both loss aversion and dread aversion; lengthening the interval between a decision and its resolution prolongs the period of dread, and this experience decreases the emotional utility of patience.

## 3 Data and sampling

We use data from the British Household Panel Survey (BHPS), a longitudinal survey measuring a wide variety of economic, social, and psychological variables among a nationally representative sample of approximately 10,000 UK households. The BHPS consists of 18 annual waves of data from 1991 to 2008. In addition, we use data from Understanding Society (USoc), a longitudinal survey of over 40,000 UK households that builds on the BHPS. USoc currently consists of 14 annual waves of data from 2009 to 2024. In Wave 2 (2010–2011), over 6000 participants from the BHPS—which ended in 2008—joined USoc. The BHPS and USoc data have been harmonized, which means we can track the original BHPS sample from 1991 to 2024. The sample used within this research is restricted to the original BHPS sample covering Great Britain. No other data restrictions were imposed. As we describe in more detail below, the longitudinal nature of the data allows us to elicit individual levels of anticipatory and reactive emotions over a number of years (Waves 1–17 of the BHPS) and prior to the elicitation of risk preferences (which was only asked in Wave 18 of the BHPS) and time preferences (which was only asked in Wave 5 of USoc). Therefore, anticipatory and reactive emotions, while not strictly exogenous, are expected to be less susceptible to reverse causality with respect to individual risk and time preferences—that is, psychological characteristics are assumed to influence risk and time preferences, rather than the reverse.

### 3.1 Open practices statement

The data used in this study are publicly available from the UK Data Archive. To access the dataset, visit:

https://beta.ukdataservice.ac.uk/datacatalogue/series/series?id=2000053#!/access‐data. Under the “Access data” section, expand the drop‐down menu and select the following dataset:

6614 – Understanding Society: Waves 1–14, 2009–2023 and Harmonised BHPS: Waves 1–18, 1991–2009.

All analysis scripts and the accompanying codebook used in this study—including the simulations script—are available on the Open Science Framework at the following link:

https://osf.io/n4c6q.

## 4 Results

### 4.1 Measuring asymmetric anticipatory emotions and asymmetric reactive emotions

To measure an individual's anticipatory emotions to contemplating losses (dread) and gains (savoring) and reactive emotions to experiencing losses (displeasure) and gains (pleasure), we use a simple approach that looks at how individuals’ current financial realizations (relative to the past year) and expectations about the next year's financial circumstances (relative to the current time) affects their current psychological well‐being. To the extent that dread outweighs savoring, forecasting financial losses should have a greater effect on current psychological well‐being, compared to forecasting gains. Similarly, to the extent that people are loss averse, current financial losses should have a greater effect on current psychological well‐being than current financial gains.

To implement this strategy, we use data from the first 17 waves (Waves 1–17) of the BHPS. This yields a sample size of 125,026 observations from 13,969 individuals. Therefore, intrinsic anticipatory and reactive emotions are constructed from an average of 9 observations per individual, with a minimum of 1 and maximum of 17 observations per person.

Financial expectations (“Looking ahead, how do you think you yourself will be financially a year from now; better than you are now, worse than you are now, or about the same?”) and financial realizations (“Would you say that you yourself are better off, worse off or about the same financially than you were a year ago?”) were measured in each wave. Psychological well‐being was measured in each wave using the General Health Questionnaire (GHQ), a 12‐item scale assessing psychological distress or “disutility” (e.g., “Have you recently lost much sleep over worry?” and “Have you recently been feeling reasonably happy, all things considered?” [reverse‐coded]). Responses are coded on a 4‐point scoring system (items scored 0‐1‐2‐3) that ranges from “strongly disagree” to “strongly agree.” Scores are then summed together, providing each respondent with a total GHQ score ranging from 0 to 36. We rescale the measure so that higher scores correspond to higher psychological well‐being or higher “utility.”

First, we calculate, for each individual, the effect on their *current* well‐being of forecasting a *better* financial situation next year (savoring gains) or a *worse* financial situation next year (dreading losses). That is, we assess the relationship between financial expectations (E) and utility (GHQ), by estimating the following linear random effects multilevel equation for the ith individual at time t:

(6) $$GHQ_{it}=b_{0}+b_{1}E_{it}^{B}+b_{2}E_{it}^{W}+b_{3}{\bar{E}}_{i}^{B}+b_{4}{\bar{E}}_{i}^{W}+b_{5}X_{it}+b_{6}{\bar{X}}_{i}+z_{i0}+z_{i1}E_{it}^{B}+z_{i2}E_{it}^{W}+\varepsilon_{it}.$$

In Eq. (6), $E_{it}^{B}$ is a dummy variable indicating a financial expectation of “better off” (for time t+1) at time t(i.e., $E_{it}^{B}=1$ if the respondent expects to be “better off”; otherwise, $E_{it}^{B}=0$). $E_{it}^{W}$ is a dummy variable indicating a financial expectation of “worse off” (i.e., $E_{it}^{W}=1$ if the respondent expects to be “worse off”; otherwise, $E_{it}^{W}=0$). Therefore, an expectation of “about the same” serves as a baseline category to which the other forecasts are compared. We include a vector of time‐varying sociodemographic and socioeconomic control variables, $X_{it}$. To alleviate concerns that the scenarios individuals consider when forecasting future financial status are not equivalent nor symmetric over future losses and gains (e.g., “a small raise from employer” vs. “starting a successful entrepreneurial venture” vs. “losing one's job” vs. “retirement”), we also include forwarded socioeconomic and sociodemographic control variables (i.e., $X_{it+1}$). To constrain all the variation to within‐person, we follow Mundlak (1978) by including the time means of all the time‐varying independent variables—that is, ${\bar{E}}_{i}^{B}$, $\;{\bar{E}}_{i}^{W}$, and ${\bar{X}}_{i}$. Finally, to allow individuals to have different psychological responses to expected income, we include both a random effect ($z_{i0}$) attached to the intercept and a random effect $(z_{i1}$ and $z_{i2}$) attached to the slopes. These random effects together allow us to estimate heterogeneity in the effect of $E_{it}^{B}$ and $E_{it}^{W}$ across individuals. Specifically, $z_{i1}$ and $z_{i2}$ reflects the difference between the average within‐person effect of anticipatory emotions, $\;b_{1}$ and $b_{2}$, and the effect for each individual. In reporting results below, we therefore use $b_{1}$ and $b_{2}$ as population‐level estimates of anticipatory emotions (savoring and dread, respectively) and $\left(b_{1}+z_{i1}\right)$ and $\left(b_{2}+z_{i2}\right)$ as their individual‐level estimates.

Next, in an identical fashion, Eq. (7) repeats the analysis for financial realizations, R:

(7) $$GHQ_{it}=c_{0}+c_{1}R_{it}^{B}+c_{2}R_{it}^{W}+c_{3}{\bar{R}}_{i}^{B}+c_{4}{\bar{R}}_{i}^{W}+c_{5}X_{it}+c_{6}{\bar{X}}_{i}+u_{i0}+u_{i1}R_{it}^{B}+u_{i2}R_{it}^{W}+\varepsilon_{it}.$$

Directly analogous to our analysis of anticipatory emotions, we use $c_{1}$ and $c_{2}$ as population‐level estimates of reactive emotions (pleasure and displeasure to gains and losses, respectively) and $\left(c_{1}+u_{i1}\right)$ and $\left(c_{2}+u_{i2}\right)$ as their individual‐level estimates. It is worth noting that the predicted individual‐level deviations from the population‐average effects—derived from Eqs. (6) and (7) and used to construct measures of asymmetric anticipatory and reactive emotions—are best linear unbiased predictions (BLUPs), which are shrunk toward the overall average effect. In particular, when data for a particular individual (or group) are sparse, the model “borrows strength” from the population‐level estimates to improve the stability of the individual‐specific predictions. This process, known as shrinkage, adjusts the individual‐level estimates toward the grand mean, reflecting the model's recognition of the limited information available for precise estimation. Such shrinkage improves the precision, reliability, and generalizability of the predictions, particularly for individuals or clusters with few observations.

We estimate both Eqs. (6) and (7) with an unstructured covariance matrix to allow for correlation between the random effect attached to the intercept and the random effects attached to the within‐person coefficients. The within‐person errors are modeled using an autoregressive structure of order 2, which is confirmed by model selection criteria (in particular, the Akaike and Bayesian information criteria). Table B1 in Appendix B of the Supporting Information provides summary statistics of the variables used in the estimation of Eqs. (6) and (7), while Regressions 1 and 2 in Table B2 in Appendix B of the Supporting Information report the respective estimated results. We now use these estimates to answer five questions motivated by our theoretical framework.

#### 4.1.1 Do anticipatory and realized gains and losses affect current (dis)utility?

From Eq. (6), the average within‐effect effects of expecting to be “worse off” ($b_{2}=-0.582,95\%\;\mathrm{CI}\;=\left[-0.493,-0.671\right],t=-12.82,p<.001$) and “better off” ($b_{1}=0.094,95\%\;\mathrm{CI}=\left[0.026,0.163\right],t=2.70,p=.007$) are both significantly different from zero and in the expected direction, providing evidence of both dread and savoring, consistent with Loewenstein (1987). Similarly, from Eq. (7), the average within‐effect effect of a current financial realization of “worse off” ($c_{2}=-1.063,95\%\;\mathrm{CI}\;=\left[-0.988,-1.139\right],t=-27.61,p<.001$) and “better off” ($c_{1}=0.519,95\%\;\mathrm{CI}=\left[0.457,0.581\right],t=16.37,p<.001$) are both significantly different from zero and again in the expected direction. Anticipatory emotions, though statistically significantly different from zero, are less powerful than realized emotions.

#### 4.1.2 Does dread loom larger than savoring?

The average within‐person coefficients on expecting “worse off” and “better off” discussed above make plain that dread is much more powerful than savoring. Indeed, the difference in absolute magnitudes of the effects is highly statistically significant ($|b_{2}\left|-\;\right|b_{1}|=0.488,95\%\;\mathrm{CI}=\left[0.368,0.607\right],t=7.99,p<.001$). If we define asymmetric anticipatory emotions at the group level as the ratio of the average within‐person coefficients associated with dread and savoring (i.e., $b_{2}/b_{1}$), the ratio is 6.19. This accords with Hardisty and Weber's (2020) lab studies, which find substantial amounts of dread but little evidence of savoring. Similarly, the difference in absolute magnitudes of the average within‐person coefficients between a current financial realization of “worse off” and “better off” is highly statistically significant ($|c_{2}\left|-\;\right|c_{1}|=0.544,95\%\;CI=\left[0.433,0.655\right],t=9.57,p<.001$). Moreover, if we define loss aversion at the group level as the ratio of the average within‐person coefficients associated with realized financial loss and gain (i.e., $c_{2}/c_{1}$), the ratio is 2.05. This is in line with the classic estimate of λ, which is approximately 2 (Kahneman & Tversky, 1979). The very large ratio of dread to savoring suggests that asymmetric anticipatory emotions are not fully explained by the loss aversion mechanism (i.e., via λ). This suggests that dread aversion (σ) is also partly accounting for these results, that is, if λσ≈6 and λ≈2, then σ≈3. That is, this suggests that not only do people experience greater dread than savoring because the *object* of dread is more psychologically potent than the object of savoring (i.e., losses loom larger than gains; λ>1) but also because dread is itself a more powerful emotion than savoring (i.e., σ>1).

#### 4.1.3 How much heterogeneity in anticipatory and reactive emotions is there across individuals?

**Fig 3:**
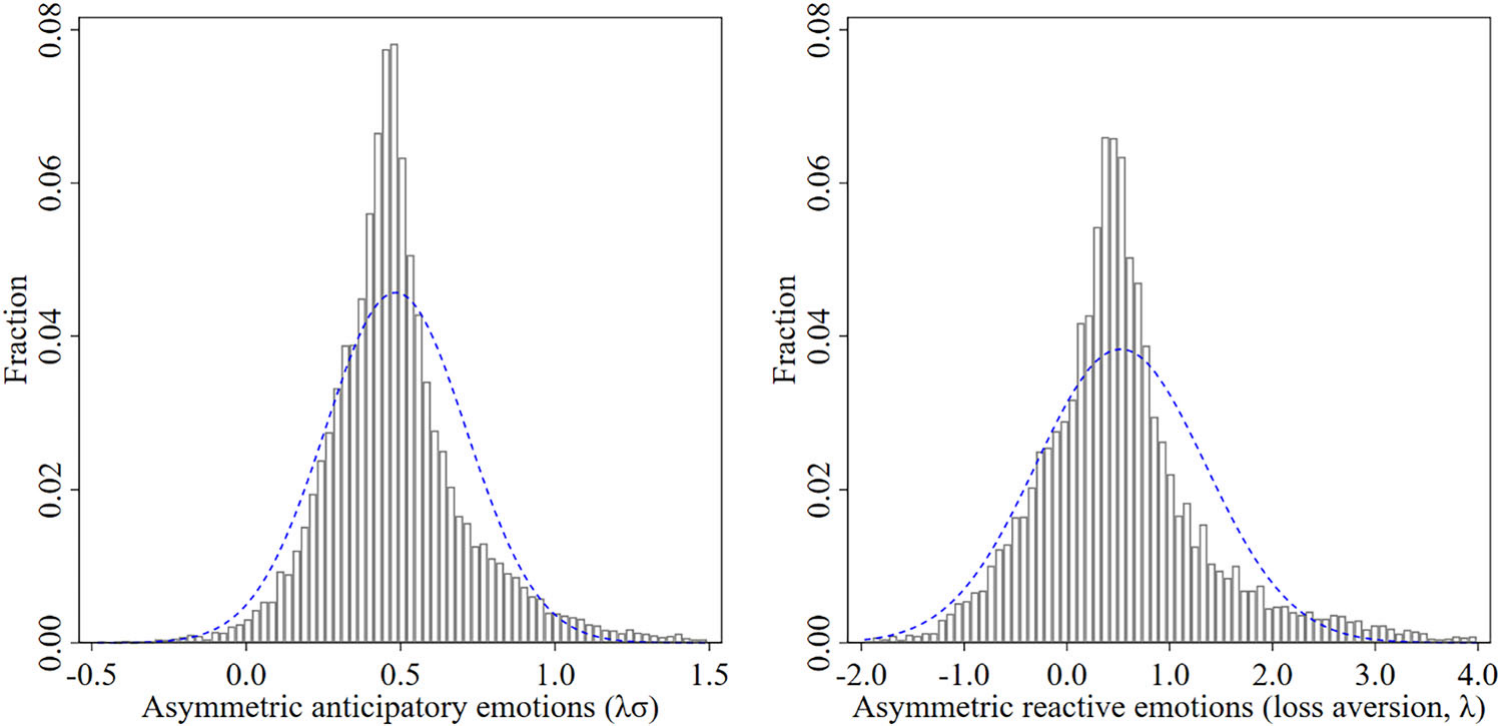
Histogram of asymmetric anticipatory emotions and asymmetric reactive emotions based on a single estimate per individual (n = 13,969). *Note*. A normal density is also plotted in each graph, with the same mean and variance as the estimated data. Higher asymmetric anticipatory emotion scores relate to higher “disutility” from anticipating income losses (dread) relative to “utility” from anticipating income gains (savoring). Higher asymmetric reactive emotion scores relate to higher “disutility” from experiencing income losses relative to “utility” from experiencing income gains.

From Eqs. (6) and (7), we can extract individual‐level estimates of the asymmetries in anticipatory and reactive emotions. Specifically, from Eq. (6), the asymmetry in anticipatory emotions (i.e., λσ) can for each individual be measured as $\left(b_{2}+z_{i2}\right)-\left(b_{1}+z_{i1}\right),$ with coefficients rescaled so that measures of anticipatory reactions to future monetary losses and gains are increasing in disutility and utility, respectively. Higher asymmetric anticipatory emotion scores relate to higher “disutility” from anticipating income losses (dread) relative to “utility” from anticipating income gains (savoring). In the same way, from Eq. (7), the asymmetry in reactive emotions (i.e., loss aversion λ) for each individual can be measured as $\left(c_{2}+u_{i2}\right)-\left(c_{1}+u_{i1}\right)$, with coefficients rescaled so that measures of reactions to current monetary losses and gains are increasing in disutility and utility, respectively. Higher loss aversion scores relate to higher “disutility” from income losses relative to “utility” from income gains. We find substantial heterogeneity across all parameters, with the exception of the parameter $u_{i1}$, which is the measure of heterogeneity in how individuals react to financial gains. Reflecting this, individual loss aversion is measured as $\left(c_{2}+u_{i2}\right)-\left(c_{1}\right)$. Fig. 3 depicts the heterogeneity in asymmetric anticipatory and reactive emotions across individuals. Here, zero represents symmetric anticipatory (left panel) and reactive (right panel) emotional responses to, respectively, anticipated or experienced changes in income. Scores above zero reflect higher “disutility” from anticipating or experiencing income losses relative to the “utility” from anticipating or experiencing income gains. Similarly, scores below zero reflect lower “disutility” from anticipating or experiencing income losses relative to the “utility” from anticipating or experiencing income gains. These individual effects provide estimates of asymmetric anticipatory and reactive emotions, net of environmental influences related to location, time, and other changes in individual circumstances. We use these estimates in the subsequent analysis as key predictors of risk and time preferences.^1^

#### 4.1.4 Are people higher in negative anticipatory emotions (dread) also higher in positive anticipatory emotions (savoring)?

If so, this would suggest that dread and savoring both emanate from the common psychological mechanism of vividly imagining the future as our theory postulates (i.e., the α parameter for vividness). Eq. (6) estimates a negative correlation between the random effect parameters, $z_{i1}$ and $z_{i2},r=-0.428,p<.001$, indicating that individuals with greater sensitivity to savoring are also more sensitive to dread. To our knowledge, this represents the first evidence that individuals who are more responsive to positive anticipatory emotions also tend to be more responsive to negative ones. Although our primary interest is in the *asymmetry* between dread and savoring—as this is what drives our theoretical results—it is useful to observe that some people appear to experience more vivid anticipatory emotions in general, perhaps due to differences in emotion regulation, more vivid mental imagery, or greater preoccupation with the future (e.g., Zabelina & Condon, 2020). The significance of this finding lies in its challenge to the prevailing view that emotional reactivity is strictly valence‐specific, with individuals predominantly sensitive to either positive or negative stimuli (e.g., Canli, Sivers, Whitfield, Gotlib, & Gabrieli, 2002; Fox, Russo, & Dutton, 2002). Instead, our results propose a novel individual difference variable—generalized sensitivity to anticipatory emotions—that transcends valence‐specific biases.

#### 4.1.5 Do people with more asymmetric reactive emotions (loss aversion) also have more asymmetric anticipatory emotions?

Theoretically, asymmetric anticipatory emotions exist in part because the *object* of dread is more psychologically potent than the object of savoring (i.e., losses loom larger than gains; λ>1) but also because dread is itself a more powerful emotion than savoring (i.e., σ>1). Therefore, we expect a moderate correlation between the psychological constructs, which correspond to λ and λσ. Indeed, we estimate a moderate correlation between asymmetric anticipatory emotions and asymmetric reactive emotions (loss aversion), r=0.466,p<.001.

### 4.2 Asymmetric anticipatory emotions, asymmetric reactive emotions, and economic preferences

Next, we determine empirically whether individual differences in loss aversion and asymmetric anticipatory emotions are associated with risk‐avoidance and impatience, as our theory suggests.

#### 4.2.1 Risk preferences

In estimating the effect of asymmetric anticipatory and reactive emotions on risk preferences, we use the general risk question (“*Are you generally a person who is fully prepared to take risks or do you try to avoid taking risks*?” from 1 (“*unwilling to take risks*”) to 10 (“*fully prepared to take risks*”)), which was only asked in the final wave (Wave 18) of the BHPS. This measure of risk has been widely validated across multiple domains and populations (Falk et al., 2018). Dohmen et al. (2011) show that this single‐item measure is a robust and consistent predictor of real‐world risky behavior—including in financial decisions, health choices, occupational paths, and driving—often performing on par with or better than incentivized experimental measures, especially when predicting out‐of‐lab behavior. However, as a general measure of risk, it does not capture domain‐specific heterogeneity in risk preferences (Weber, Blais, & Betz, 2002). Furthermore, as a subjective self‐report, it is susceptible to individual differences in interpretation, including reference‐point effects (Lira et al., 2022). Despite these drawbacks, its predictive validity and ease of implementation make it a valuable proxy for risk preferences in large‐scale observational research, where experimental elicitation is often infeasible. Fig. B1 in Appendix B of the Supporting Information shows the distribution of general risk attitudes for our sample. It is worth noting that we do lose a significant proportion of individuals in our second‐stage analysis owing to sample attrition. However, sample attrition rates in the BHPS are generally low and comparable to those achieved in other similar household panels. In common with nearly all previously published research using this data source, attrition is assumed to be a random event.

Table 3 reports the key results. Although the dependent variable (risk attitudes) is ordinal, we use ordinary least squares (OLS) regression for ease of interpretation. We standardize measures of asymmetric anticipatory and reactive emotions and present results across nested models with increasing sets of control variables. These include age, sex, trait optimism and pessimism, state psychological well‐being, anxiety, and a range of other personal, socioeconomic, and sociodemographic characteristics, which are described in the table caption. Importantly, the results presented in Table 3 are quantitatively and qualitatively similar when using alternative models, functional forms, and categorizations of the dependent variable: ordered logistic regression; interval regression; logistic regression after dichotomizing the risk variable into high versus low risk (equal to 1 if the respondent is in the highest quartile of the risk distribution and 0 otherwise); winsorizing the asymmetric anticipatory and reactive emotions at the 5th and 95th percentiles to mitigate the potential impact of outliers; and when including asymmetric anticipatory emotions by entering their components—estimated savoring and dread—as separate regressors. These robustness checks are reported in Tables B4 to B8 in Appendix B of the Supporting Information.^2^

**Table 3:** Predictors of risk preferences

Dependent Variable: Willingness to Take Risks in General				
Predictors	Regression 1	Regression 2	Regression 3	Regression 4
Asymmetric anticipatory emotions	−0.130^***^	−0.089^***^	−0.073^***^	−0.079^***^
	[−5.630]	[−3.603]	[−2.977]	[−3.245]
Asymmetric reactive emotions		−0.111^***^	−0.102^***^	−0.093^***^
		[−4.197]	[−3.912]	[−3.555]
				
Exogeneous controls	Yes	Yes	Yes	Yes
Trait optimism/anxiety/state General Health Questionnaire (GHQ)	No	No	Yes	Yes
Sociodemographic controls	No	No	No	Yes
*R*‐squared	0.085	0.088	0.120	0.144
Observations	6839	6839	6839	6839
Individuals	6839	6839	6839	6839

*Note*. Entries are results from ordinary least squares (OLS) regressions estimating the determinants of the general risk question—ranging from 1 (“*unwilling to take risks*”) to 10 (“*fully prepared to take risks*”). Unadjusted coefficients are reported, with *t*‐statistics based on robust standard errors shown in brackets. Asymmetric anticipatory and reactive emotions are standardized and measures higher “disutility” from anticipating or experiencing income losses relative to “utility” from income gains. Exogenous controls include age (entered in both linear and quadratic form) and sex. Trait optimism/pessimism is captured by two variables, measured as the individual's time‐averaged (Waves 1–17, British Household Panel Survey [BHPS]) financial expectations of “better off” and “worse off.” Anxiety is a binary variable equal to one if the respondent ever reported anxiety or depression during Waves 1–17 of the BHPS and zero otherwise. State GHQ is calculated as the individual's current GHQ score minus their time‐averaged (Waves 1–17, BHPS) GHQ score. Sociodemographic controls include current education level (highest academic qualification), current marital status, current housing tenure, current labor market status, logarithm of monthly household income [deflated] for the current year, current number of children under the age of 16 present in the household, and square root of current household size. We use analytical weights to account for variation in the number of observations per individual when estimating asymmetric anticipatory and reactive emotions. Individuals observed more frequently are given greater weight, reflecting the higher precision of their emotion estimates. Full results are available in Table B3 in Appendix B of the Supporting Information.

* *p* < .1 ** *p* < .05 *** *p* < .01

As shown in all regressions of Table 3, higher asymmetric anticipatory emotions have a strong negative effect on risk preferences. Specifically, in Regression 1 of Table 3—where we estimated the relationship between asymmetric anticipatory emotions and risk preferences controlling for our exogenous regressors (i.e., age and sex)—a 1 standard deviation (SD) increase in asymmetric anticipatory emotions reduces willingness to take risks by –0.130. Alternatively, those high on asymmetric anticipatory emotions (+2 SDs from the mean) have a predicted willingness to take risks of 5.11, while those low on asymmetric anticipatory emotions (–2 SDs from the mean) have a predicted willingness to take risks of 5.63. In Regression 2, we control for asymmetric reactive emotions (i.e., loss aversion); therefore, the coefficient attached to asymmetric anticipatory emotions now measures the effect of dread aversion on risk preferences. Here, and consistent with our theoretical approach, asymmetric reactive emotions decrease people's willingness to take risks, and these effects are quantitatively similar to the effects of dread aversion. In addition, the effect of asymmetric anticipatory emotions is partially due to loss aversion, with a 1 SD increase in asymmetric anticipatory emotions now reducing willingness to take risks by –0.089. Alternatively, those low on asymmetric anticipatory emotions (–2 SDs) have a predicted willingness to take risks of 5.19, while those high on asymmetric anticipatory emotions (+2 SDs) have a predicted willingness to take risks of 5.55. Taken together, these results suggest that, although moderately correlated, asymmetric anticipatory emotions and loss aversion capture different aspects of behavior and therefore have complementary predictive power for risk preferences.

While the OLS estimates provide a useful benchmark, the ordered logit model accounts for the ordinal nature of the outcome variable and offers alternative interpretations of effect sizes in terms of changes in the predicted probability of being in higher or lower outcome categories. When estimating Regression 2 using ordered logistic regression (Table B4 in Appendix B of the Supporting Information), those low (vs. high) on asymmetric anticipatory emotions have a 40.05% higher predicted probability of reporting the highest risk‐taking category and a 27.80% lower probability of the lowest risk‐taking category. To put these effects in context, all else being equal and consistent with previous research (Charness & Gneezy, 2012; Dohmen et al., 2011; Eckel & Grossman, 2002), men have a 79.52% higher predicted probability of reporting the highest risk‐taking category and a 43.37% lower probability of the lowest risk‐taking category.

In Regressions 3 and 4 of Table 3, we add further controls for trait optimism and pessimism, anxiety, state GHQ, and sociodemographic controls. The addition of these extra control variables has little effect on the estimated effect of dread aversion on risk preferences.

To explore the individual contributions of dread and savoring, we also fit a series of models with these variables as separate regressors (Table B8 in Appendix B of the Supporting Information). In Regression 4 of Table B8, while the coefficients on dread (b=−0.138,95%CI=[−0.214,−0.062],t=−3.57,p<.001) and savoring (b=0.074,95%CI=[0.006,0.141],t=2.13,p=.033) have opposite signs, the absolute magnitudes of the effects are not equal (p=.011). Here, holding savoring constant, those high on dread (+2 SDs) have a predicted willingness to take risks of 5.10, while those low on dread (–2 SDs) have a predicted willingness to take risks of 5.65. These results highlight that dread and savoring have distinct associations with risk preferences: stronger dread reduces the willingness to take risks more substantially than savoring increases it. This underscores the dominant role of dread in decision‐making under risk.

Last, it is important to note that while our measures of asymmetric anticipatory and reactive emotions are best linear unbiased predictions (BLUPs), they are subject to estimation error, as they are estimated rather than directly observed. Under classical measurement error, the OLS regression estimate of asymmetric anticipatory emotions on risk preferences will be biased toward zero. In this view, using an errors‐in‐variables approach, we report in Fig. B3 (Panel A) in Appendix B of the Supporting Information the estimates of Regression 4 from Table 3 for various assumptions concerning the importance of measurement error. Specifically, we plot the relationship between asymmetric anticipatory emotions and risk preferences for different levels of reliability, r=1.0,0.7,0.5, in asymmetric anticipatory emotions. Lower assumed reliability leads to *larger* estimated effect sizes. When asymmetric anticipatory emotions are assumed to be 50% due to measurement error, asymmetric anticipatory emotions continue to significantly affect risk preferences, with a substantially larger coefficient than in Table 3 (b=−0.200,95%CI=[−0.323,−0.079],t=−3.23,p=.001). Here, those high on asymmetric anticipatory emotions (+2 SDs) have a predicted willingness to take risks of 4.97, while those low on asymmetric anticipatory emotions (–2 SDs) have a predicted willingness to take risks of 5.77.^3^ The results presented in Table 3 are therefore likely to be lower bound estimates of the effect of asymmetric anticipatory emotions on risk preferences.

#### 4.2.2 Time preferences

In estimating the effect of asymmetric anticipatory and reactive emotions on time preferences, we operationalize time preferences as an individual's tolerance for delayed gratification—to resist the temptation of an immediate reward in preference for a later reward. In Wave 5 of USoc (2013), all adults completed a 10‐item delayed gratification scale (*α* = 0.66), taken from a longer scale (Hoerger, Quirk, & Weed, 2011). Items included: “*I would have a hard time sticking with a special, healthy diet*” [reverse‐coded]; “*I try to spend money wisely*”; “*I have given up physical pleasure or comfort to reach my goals*”; “*I try to consider how my actions will affect other people in the long‐term*”; “*I cannot be trusted with money*” [reverse‐coded]; “*I do not consider how my behaviour affects other people*” [reverse‐coded]; “*I cannot motivate myself to accomplish long‐term goals*” [reverse‐coded]; “*I have always tried to eat healthy because it pays off in the long run*”; “*When faced with a physically demanding chore, I always tried to put off doing it*” [reverse‐coded]; and “*I have always felt like my hard work would pay off in the end*,” all on 0 (“*strongly disagree*”) to 10 (“*strongly agree*”) scales. The scores are then summed to construct a scale from 0 to 100 that increases with patience.

Fig. B2 in Appendix B of the Supporting Information shows the distribution of time preferences in our sample. To assess its predictive validity, we examine correlations between our measure and key life outcomes previously associated with time preferences in the literature—such as savings behavior, educational attainment, marital status, smoking, BMI (Body Mass Index), and obesity (Basiglio, Foresta, & Turati, 2024; DellaVigna & Paserman 2005; Golsteyn, Grönqvist, & Lindahl, 2014; Tasoff & Zhang, 2022). In line with prior findings, outcomes typically requiring greater patience—saving, being a university graduate, and being married—are positively correlated with our patience measure (0.069<r<0.148,p<.001), while smoking‐related outcomes, higher BMI, and obesity, are negatively correlated, as expected (−0.153<r<−0.095,p<.001).

Table 4 reports the key results. As time preferences are approximately linear and normally distributed, we use OLS regression. We standardize measures of asymmetric anticipatory and reactive emotions and, as for Table 3, present results across nested models with increasing sets of control variables. The results presented in Table 4 are quantitatively and qualitatively similar when using alternative models, functional forms, and categorizations of the dependent variable: ordered logistic regression after categorizing the patience measure into quartiles; logistic regression after dichotomizing the patience variable into high versus low patience (equal to one if the respondent is in the highest quartile of the patience distribution, and zero otherwise); winsorizing the asymmetric anticipatory and reactive emotions at the 5th and 95th percentiles to mitigate the potential impact of outliers; and when including asymmetric anticipatory emotions by entering their components—estimated savoring and dread—as separate regressors. These robustness checks are reported in Tables B10 to B13 in Appendix B of the Supporting Information.

**Table 4:** Predictors of time preferences

Dependent Variable: Delayed Gratification Scale				
Predictors	Regression 1	Regression 2	Regression 3	Regression 4
Asymmetric anticipatory emotions	−1.030^***^	−0.785^***^	−0.738^***^	−0.827^***^
	[−6.447]	[−4.561]	[−4.306]	[−4.964]
Asymmetric reactive emotions		−0.691^***^	−0.620^***^	−0.422^**^
		[−3.647]	[−3.210]	[−2.238]
				
Exogeneous controls	Yes	Yes	Yes	Yes
Trait optimism/anxiety/state GHQ	No	No	Yes	Yes
Sociodemographic controls	No	No	No	Yes
*R*‐squared	0.018	0.022	0.040	0.092
Observations	4173	4173	4173	4173
Individuals	4173	4173	4173	4173

*Note*. Entries are results from OLS regressions estimating the determinants of the delayed gratification scale—ranging from 0 (impatient) to 100 (patient). Unadjusted coefficients are reported, with *t*‐statistics based on robust standard errors shown in brackets. Asymmetric anticipatory and reactive emotions are standardized and measure higher “disutility” from anticipating or experiencing income losses relative to “utility” from income gains. We use analytical weights to account for variation in the number of observations per individual when estimating asymmetric anticipatory and reactive emotions. Individuals observed more frequently are given greater weight, reflecting the higher precision of their emotion estimates. Full results are available in Table B9 in Appendix B of the Supporting Information. For details about the control variables, see the notes for Table 3.

∗p<.1∗∗p<.05∗∗∗p<.01.

As shown in all regressions of Table 4, higher asymmetric anticipatory emotions have a strong negative effect on patience. Specifically, in Regression 1 of Table 4—where we estimated the relationship between asymmetric anticipatory emotions and patience controlling for our exogenous regressors (i.e., age and sex)—a 1 SD increase in asymmetric anticipatory emotions reduces patience by −1.030. Alternatively, those low on asymmetric anticipatory emotions (−2 SDs from the mean) have a predicted patience score of 64.17, while those high on asymmetric anticipatory emotions (+2 SDs from the mean) have a predicted patience score of 68.29. Also, women tend to be more patient, as in prior work (e.g., Dittrich & Leipold, 2014).

In Regression 2, we control for asymmetric reactive emotions (i.e., loss aversion); therefore, the coefficient attached to asymmetric anticipatory emotions now measures the effect of dread aversion on patience. Here, asymmetric reactive emotions are associated with less patience, and these effects are quantitatively similar to the effects of dread aversion. In addition, the effect of asymmetric anticipatory emotions is partially due to loss aversion, with a 1 SD increase in asymmetric anticipatory emotions now reducing patience by −0.785. Alternatively, those high on asymmetric anticipatory emotions have a predicted patience score of 64.65, while those low on asymmetric anticipatory emotions have a predicted patience score of 67.79. These results confirm that dread aversion and loss aversion have complementary predictive power for economic preferences.

For an alternative interpretation of effect sizes, we estimated Regression 2 using ordered logistic regression (Table B10 in Appendix B of the Supporting Information) after categorizing the patience measure into quartiles. Those low (vs. high) on asymmetric anticipatory emotions have a 38.85% higher predicted probability of being in the highest quartile of the patience distribution.

In Regressions 3 and 4 of Table 4, we add further controls for trait optimism and pessimism, anxiety, state GHQ, and sociodemographic controls. The addition of these extra control variables has little effect on the estimated effect of dread aversion on patience.

To explore the individual contributions of dread and savoring, we included these variables as separate regressors (Table B13 in Appendix B of the Supporting Information). In Regression 4 of Table B13, the coefficients on dread (b=−1.398,95%CI=[−1.911,−0.886],t=−5.35,p<.001) and savoring (b=0.863,95%CI=[0.388,1.337],t=3.56,p<.001) have opposite signs; however, the absolute magnitudes of the effects are not equal (p=.002). This opposite and asymmetric effect provides further evidence of the disproportionate effect that dread has on economic preferences.

Last, Panel B of Fig. B3 in Appendix B of the Supporting Information reports the results of Regression 4 in Table 4 for our errors‐in‐variables approach, which makes various assumptions concerning the importance of measurement error. When asymmetric anticipatory emotions are assumed to be 50% due to measurement error, asymmetric anticipatory emotions significantly affect patience, with a larger coefficient than in Table 4 (b=−2.095,95%CI=[−2.927,−1.262],t=−4.93,p<.001). Here, those low on asymmetric anticipatory emotions (–2 SDs) have a predicted patience score of 62.07, while those high on asymmetric anticipatory emotions (+2 SDs) have a predicted patience score of 70.45. As with risk preferences, the results presented in Table 4 are likely to be lower bound estimates of the effect of dread aversion on patience.

### 4.3 Asymmetric anticipatory emotions, asymmetric reactive emotions, and personality

Here, we empirically test whether the link between asymmetric emotions and economic preferences remains once we control for broad‐based personality traits, specifically the Big Five. For instance, neuroticism—a trait strongly associated with anxiety (Jylhä & Isometsä, 2006) and characterized by frequent and intense negative emotions (McCrae & Costa, 1997)—is linked to heightened neural responses to negative stimuli and increased sensitivity to potential threats (DeYoung, 2015; Ormel et al., 2013; Vogeltanz & Hecker, 1999), as well as greater pessimism and uncertainty about the future (Hirsh & Inzlicht, 2008; Marshall et al., 1992). As such, a preference for immediate rewards or risk avoidance may reflect an adaptive response to environmental unpredictability and perceived threat (Ellis, Figueredo, Brumbach, & Schlomer, 2009). This perspective aligns with our central thesis, which posits that individuals may exhibit greater impatience and risk avoidance because they are motivated to avoid the emotional discomfort of anticipating negative outcomes. In line with these perspectives, higher neuroticism has been found to predict lower risk‐taking (Lauriola & Levin, 2001; Smith, Ebert, & Broman‐Fulks, 2016) and a stronger preference for immediate rewards (Keidel, Lu, Suzuki, Murawski, & Ettinger, 2024; Manning et al., 2014). If personality traits like neuroticism are strongly linked to anticipatory and reactive emotions, they may serve as substitutes in explaining heterogeneity in economic preferences. However, if these traits and emotions reflect distinct psychological systems, they may instead exert complementary predictive power.

To explore this question, we draw on Wave 15 of the BHPS, which includes the 15‐item short form of the Big‐Five Inventory. Table 5 reports the correlation matrix between our measures of asymmetric emotions, risk‐taking, patience, and the Big Five personality dimensions. The strongest associations emerge for neuroticism, which—as in prior research (Keidel et al., 2024; Lauriola & Levin, 2001; Manning et al., 2014; Smith et al., 2016)—is negatively correlated with both patience and risk‐taking.

**Table 5:** Pairwise correlations

Variables	(1)	(2)	(3)	(4)	(5)	(6)	(7)	(8)
(1) Willingness to take risks in general								
(2) Delayed gratification scale	0.054^***^							
(3) Asymmetric anticipatory emotions	−0.079^***^	−0.110^***^						
(4) Asymmetric reactive emotions	−0.071^***^	−0.101^***^	0.466^***^					
(5) Openness	0.242^***^	0.177^***^	−0.012	0.006				
(6) Neuroticism	−0.209^***^	−0.148^***^	0.254^***^	0.259^***^	−0.031^***^			
(7) Extraversion	0.192^***^	0.052^***^	−0.035^***^	−0.043^***^	0.318^***^	−0.130^***^		
(8) Conscientious	0.020^*^	0.284^***^	−0.062^***^	−0.041^***^	0.247^***^	−0.108^***^	0.240^***^	
(9) Agreeableness	−0.053^***^	0.178^***^	−0.030^***^	−0.013	0.236^***^	−0.013	0.193^***^	0.406^***^

*Note*. Willingness to take risks in general ranges from 1 (“*unwilling to take risks*”) to 10 (“*fully prepared to take risks*”). The delayed gratification scale ranges from 0 (impatient) to 100 (patient). Asymmetric anticipatory and reactive emotions measure higher “disutility” from anticipating or experiencing income losses relative to “utility” from income gains. Personality traits—openness, neuroticism, extraversion, conscientiousness, agreeableness—are measured using the short 15‐item Big‐Five inventory (BFI‐15). Each trait is based on a level of agreement with three statements, assessed on a 7‐point scale and averaged across items.

∗p<.1∗∗p<.05∗∗∗p<.01.

Tables 6 and 7 report the regressions with the same specification as Tables 3 and 4, respectively, but including standardized variables for openness, neuroticism, extraversion conscientious, and agreeableness. The results in Tables 6 and 7 show that personality factors contribute to the link between risk and time preferences and asymmetric emotions. Specifically, the coefficients on the asymmetric emotions are reduced in size when compared to Tables 3 and 4. Using the approach proposed by Young and Holsteen (2017)—designed to analyze how the introduction of a certain control variable changes the coefficient of interest—the most influential personality factor on the coefficient estimates of asymmetric anticipatory emotions is neuroticism. Specifically, from Regression 1 of Table 3, the coefficient on asymmetric anticipatory emotions is −0.130. The influence estimate for neuroticism shows that, all else equal, when controlling for neuroticism the coefficient on asymmetric anticipatory emotions increases by 0.090. In terms of time preferences, the equivalent influence estimate of neuroticism is 0.279, which can be assessed relative to the coefficient on asymmetric anticipatory emotions from Regression 1 of Table 4, −1.030. In contrast, the remaining personality factors have influence estimates that are approximately zero.

**Table 6:** Predictors of risk preferences

Dependent Variable: Willingness to Take Risks in General				
Predictors	Regression 1	Regression 2	Regression 3	Regression 4
Asymmetric anticipatory emotions	−0.045^**^	−0.032	−0.030	−0.035
	[−2.018]	[−1.325]	[−1.257]	[−1.476]
Asymmetric reactive emotions		−0.040	−0.047^*^	−0.045^*^
		[−1.588]	[−1.872]	[−1.764]
Openness	0.394^***^	0.394^***^	0.377^***^	0.334^***^
	[12.896]	[12.907]	[12.290]	[10.562]
Neuroticism	−0.395^***^	−0.387^***^	−0.333^***^	−0.318^***^
	[−13.326]	[−12.942]	[−10.838]	[−10.369]
Extraversion	0.235^***^	0.234^***^	0.222^***^	0.229^***^
	[7.892]	[7.872]	[7.562]	[7.803]
Conscientious	−0.027	−0.028	−0.036	−0.043
	[−0.893]	[−0.917]	[−1.180]	[−1.424]
Agreeableness	−0.177^***^	−0.176^***^	−0.172^***^	−0.155^***^
	[−5.573]	[−5.561]	[−5.543]	[−5.026]
Exogeneous controls	Yes	Yes	Yes	Yes
Trait optimism/anxiety/state GHQ	No	No	Yes	Yes
Sociodemographic controls	No	No	No	Yes
*R*‐squared	0.173	0.174	0.189	0.202
Observations	6544	6544	6544	6544
Individuals	6544	6544	6544	6544

*Note*. Entries are results from OLS regressions estimating the determinants of the general risk question—ranging from 1 (“*unwilling to take risks*”) to 10 (“*fully prepared to take risks*”). Unadjusted coefficients are reported, with *t*‐statistics based on robust standard errors shown in brackets. Asymmetric anticipatory and reactive emotions are standardized and measure higher “disutility” from anticipating or experiencing income losses relative to “utility” from income gains. Personality traits—openness, neuroticism, extraversion, conscientiousness, and agreeableness—are measured using the short BFI‐15. Each trait is based on a level of agreement with three statements, assessed on a 7‐point scale and averaged across items. We use analytical weights to account for variation in the number of observations per individual when estimating asymmetric anticipatory and reactive emotions. Individuals observed more frequently are given greater weight, reflecting the higher precision of their emotion estimates. For details about the control variables, see the notes for Table 3.

∗p<.1∗∗p<.05∗∗∗p<.01.

**Table 7:** Predictors of time preferences

Dependent Variable: Delayed Gratification Scale				
Predictors	Regression 1	Regression 2	Regression 3	Regression 4
Asymmetric anticipatory emotions	−0.628^***^	−0.465^***^	−0.482^***^	−0.580^***^
	[−4.077]	[−2.857]	[−2.976]	[−3.644]
Asymmetric reactive emotions		−0.511^***^	−0.500^***^	−0.319^*^
		[−2.773]	[−2.675]	[−1.740]
Openness	1.847^***^	1.862^***^	1.932^***^	1.346^***^
	[8.517]	[8.608]	[8.850]	[6.025]
Neuroticism	−1.522^***^	−1.426^***^	−1.233^***^	−1.095^***^
	[−7.465]	[−6.953]	[−5.834]	[−5.296]
Extraversion	−0.853^***^	−0.864^***^	−0.879^***^	−0.711^***^
	[−4.121]	[−4.170]	[−4.245]	[−3.469]
Conscientious	2.926^***^	2.929^***^	2.911^***^	2.972^***^
	[12.243]	[12.245]	[12.208]	[12.701]
Agreeableness	0.585^**^	0.591^**^	0.635^***^	0.873^***^
	[2.540]	[2.564]	[2.747]	[3.841]
Exogeneous controls	Yes	Yes	Yes	Yes
Trait optimism/anxiety/state GHQ	No	No	Yes	Yes
Sociodemographic controls	No	No	No	Yes
*R*‐squared	0.131	0.133	0.146	0.188
Observations	4056	4056	4056	4056
Individuals	4056	4056	4056	4056

*Note*. Entries are results from OLS regressions estimating the determinants of the general risk question—ranging from 0 (impatient) to 100 (patient). Unadjusted coefficients are reported, with *t*‐statistics based on robust standard errors shown in brackets. Asymmetric anticipatory and reactive emotions are standardized and measure higher “disutility” from anticipating or experiencing income losses relative to “utility” from income gains. Personality traits—openness, neuroticism, extraversion, conscientiousness, and agreeableness—are measured using the short BFI‐15. Each trait is based on a level of agreement with three statements, assessed on a 7‐point scale and averaged across items. We use analytical weights to account for variation in the number of observations per individual when estimating asymmetric anticipatory and reactive emotions. Individuals observed more frequently are given greater weight, reflecting the higher precision of their emotion estimates. For details about the control variables, see the notes for Table 3.

∗p<.1∗∗p<.05∗∗∗p<.01.

Interestingly, neuroticism contributes more to the effect of asymmetric anticipatory emotions on risk preferences than on time preferences. Indeed, in Regressions 2 to 4 of Table 6, the effect of asymmetric anticipatory emotions on risk preferences is no longer statistically significant. Speculatively, the remaining effect of asymmetric anticipatory emotions on time preferences, even after accounting for neuroticism, may be due to the “mixed” nature of positive anticipation. As discussed earlier, positive anticipatory emotions are weaker than negative ones because anticipating a positive outcome is a mixture of positive (savoring) and negative (impatience) emotions (Nowlis et al., 2004)—thus asymmetric anticipatory emotions are conceptually linked to impatience over‐and‐above the dread captured by neuroticism.

Overall, these results therefore provide an additional channel through which neuroticism affects economic preferences, that is, through an asymmetric anticipatory sensitivity to potential future negative outcomes relative to positive outcomes. Moreover, these findings add to the to the emerging literature on how variables from formal decision theory are meaningfully related to personality variables (Rustichini, DeYoung, Anderson, & Burks, 2016).

## 5 Discussion

We gain utility not only from consuming experiences but from anticipating our consumption. But all anticipatory emotions are not created equal. This article presents the first large‐scale econometric evidence that dread of imagined bad futures predominates over savoring of imagined good futures—most people are not just *loss averse*, but also *dread averse*—adding to prior evidence from lab studies that anticipatory emotions are on average asymmetric (Hardisty & Weber, 2020). We further show that asymmetric anticipatory emotions are linked to risk‐avoidance and impatience. Our model explains why: when dread outweighs savoring, downside risks loom larger and delays are more aversive. Thus, we provide a unified explanation for why these economic preferences are related. While we leverage rich longitudinal data—with emotions measured before risk and time preferences—the observational nature of our analysis means the results are ultimately correlational, not causal.

### 5.1 Toward a process model of anticipatory and consumption emotions

Section 2 presented a formal model of reactive and anticipatory emotions in a utility‐maximizing framework of the sort commonly used in economics. Yet this model is an idealization of cognitive processes rather than a process model itself. How might assessments of consumption and anticipatory utility be realized in a boundedly rational cognitive agent, and how might such processes impose boundary conditions on the model? In particular, where do anticipatory emotions come from, and what factors influence how they manifest in particular situations?

As a starting point, consider how the model computes anticipatory utility (Eq. 2 in Section 2.1). It computes the imagined (dis)utility each at each timepoint, weighs that utility by a function of its probability, and discounts that utility because it accrues in the future; finally, that product is integrated from the time of choice until the outcome is realized. The most mysterious part of this process is the imagination of utility, which is further specified in Eq. (3). The model assumes this is a function of four psychological forces—the value associated with the outcome (possibly, but not necessarily, using the same value function for an actually experienced outcome), the vividness of one's imagination, fluctuations of forecasted attention between the time of decision and the outcome, and the extent to which dread outweighs savoring. In the Introduction, we discussed why dread is thought to be a more powerful emotion than savoring. We now briefly consider what is known about these other forces.

A plausible simplifying assumption is that anticipatory emotions are themselves viscerally felt forecasts of future reactive emotions; that is, the value function for anticipation may be the same as that for consumption. (If so, then *expected* anticipatory emotions are a forecast of a forecast.) Whatever process we use to forecast reactive emotions at the time of choice may be the same process we use when experiencing anticipatory emotions after the choice but before the outcome is realized. How do people forecast such emotions? It is clear that we can experience dread or savoring of events that we have never experienced, such as Loewenstein's (1987) kiss from a celebrity or, indeed, our own mortality. These prior beliefs may come from intuitive theories, testimony from others, or evolved instincts. It seems likely that we can learn from repeated experience to tame our anticipatory emotions to more accurately reflect the true value of our experiences; still, prior beliefs seem to be quite sticky in this domain since some affective forecasting errors are highly resistant to correction (Wilson & Gilbert, 2003).

One account of these processes is Conviction Narrative Theory (CNT; Johnson et al., 2023). CNT is a sociocognitive theory of choice under uncertainty, which focuses particularly on decisions that require commitment over an extended period of time. On this approach, decision‐makers choose among alternative outcomes by assembling “narratives”—structured hypotheses about a decision context incorporating causal knowledge and analogies to similar situations. Once the best narrative is selected to explain a situation, that narrative is “run forward” through a process of mental simulation or imagination (e.g., Hegarty, 2004), and the imagined future is then appraised via affective mechanisms similar to those we use to evaluate actually present stimuli. CNT was originally conceptualized in terms of reactive emotions—that is, people imagine how they would feel *once the outcome occurs*. But the same basic machinery could be extended if people form narratives about how their anticipatory emotions evolve between the time of a choice and its outcome.

So far, we have considered what determines anticipatory affect itself. But our approach requires that people *forecast* their anticipation; these forecasts in turn depend on the expected vividness of imagination and on the expected attention paid to that outcome throughout the delay. We conjecture that the vividness of affective imagination is linked to the vividness of mental imagery, which is rooted in neural processing in the frontal, parietal, and visual cortex (Dijkstra, Bosch, & van Gerven, 2017). However, even the most vivid imagination will only produce anticipatory emotions when a person is thinking about the future outcome. Thus, mind‐wandering (Smallwood & Schooler, 2015) is likely to lead to more frequent anticipation, and mindfulness (Shapiro, Carlson, Astin, & Freedman, 2006) to less. Given that dread is a more powerful emotion than savoring, it follows that mind‐wandering should typically lead to more negative affect and mindfulness to more positive affect—both robust empirical findings (Killingsworth & Gilbert, 2010; Tomlinson et al., 2018). We predict that individual differences in dread aversion would moderate both of these effects.

Do people use intuitive theories of cognition to forecast their anticipatory emotions? Our research group's recent work on “cognitive forecasting” suggests that they do (Demircioglu, Dawson, & Johnson, 2024). That work revealed individual differences in how people believe that attention, expectations, and risk preferences are related. One group of participants believed that paying greater attention (the equivalent of having a high α in our model) consistently led to more negative affect, regardless of whether expectations of a gain or loss are high or low, whereas other participants believed that attention was an emotional boon for optimists and a bane for pessimists. Most importantly, these beliefs about the relationship between cognitive forecasts and affective forecasts translated into predictions about risk preferences. Those who believed that attention was always emotionally deleterious linked attention to risk‐avoidance for both optimists and pessimists, whereas those who believed that attention could be either beneficial or harmful linked attention to risk‐avoidance for pessimists but to risk‐seeking for optimists.

Going beyond a process model, it is also useful to consider the functional and developmental origins of dread aversion. From an evolutionary perspective, the existence of a heterogeneous trait (such as personality or economic preferences) suggests trade‐offs such that neither phenotype is strictly preferable (Nettle, 2006). One possibility is that differences in risk and time preferences—and the affective processes that govern those preferences, as shown in our model—are advantageous in different sorts of environments (Boon‐Falleur, Baumard, & André, 2025). Moreover, these processes may be calibrated during development based on early‐life environments. For instance, childhood adversity can serve as a forecast of a harsh environment, leading to the adoption of fast life‐history strategies, such as heightened sensitivity to threat (Kraus, Horberg, Goetz, & Keltner, 2011). The uncertainty management perspective (Amir & Jordan, 2017; Amir, Jordan, & Rand, 2018) similarly views early stress as a signal of future danger, shaping preferences that help manage uncertainty, such as impatience and risk aversion. Future work might examine the potential adaptive value of two affective levers—dread aversion and loss aversion—that may independently calibrate risk and time preferences.

### 5.2 Affective imagination theory

Economic preferences have been criticized as lacking a grounding in deeper psychological processes (Almlund, Duckworth, Heckman, & Kautz, 2011). We show that risk and time preferences are partly rooted in affective forecasts, grounding behavioral economics in affective and cognitive science (e.g., Berns et al., 2006; Johnson, Bilovich, & Tuckett, 2023). In forging this link, we motivate many directions for future research.

First—given that most decisions involve both risk and delay—our proposal for why asymmetric anticipatory emotions unify risk and time preferences suggests explanations for other phenomena. In ongoing work, we are extending the model proposed in Section 2 and further explored in Appendix A of the Supporting Information into a broader account of risk and time preferences called *Affective Imagination Theory*. This theory aims to unify a number of hitherto disparate phenomena.

*Sign effect*. As Hardisty and Weber (2020; see also Molouki et al., 2019) argue, dread aversion explains why people discount gains at a faster rate than losses—the *sign effect* (Thaler, 1981). Since contemplating future losses produces negative affect, we are less prone to delay those losses than we would be without anticipation, decreasing the discount rate; the corresponding positive affect from contemplating gains is weaker, so discount rates remain high.

*Certainty effect*. People pay a premium to *eliminate* rather than *reduce* risks (the *certainty effect*; Kahneman & Tversky, 1979)—for example, a much larger willingness to pay to reduce a risk from 1% to 0% than from 2% to 1%, or conversely a much larger willingness to pay to increase the chance of a benefit from 0% to 1% rather than 1% to 2%, despite these pairs of improvements having the same expected value. This follows immediately from the idea that even a small probability of a bad effect licenses savoring and dread, which are absent entirely when the event has a 0% probability. Decisions are often more sensitive to whether something is *possible* rather than its probability (Loewenstein et al., 2001). Our theory makes the further prediction that the certainty effect would be larger for losses than for gains since the disutility associated with dread (and hence the utility of paying to reduce a risk to 0%) is higher than the corresponding utility for savoring. There is some support for this prediction (Verschoor & D'Exelle, 2020), but future research should target this question directly.

*Present bias*. People pay a premium to *eliminate* rather than *reduce* delay (*present bias* or *hyperbolic discounting*; Loewenstein & Prelec, 1992). Our theory provides an analysis of this bias in time preferences that is pleasingly parallel to the certainty effect for risk preferences. As any delay will generate a license to engage in anticipatory emotions, delays are aversive if dread outweighs savoring. But just as our imaginations are often sensitive to possibilities rather than probabilities, we may be sensitive to the *existence* of a delay rather than its *magnitude*—a prediction consistent with prior evidence of duration neglect in retrospective (rather than prospective) contexts (e.g., Frederickson & Kahneman, 1993). Thus, we would be willing to pay more to eliminate dread‐inducing delays rather than to reduce the duration of such a delay.

Second, our theory makes novel predictions and raises new research questions.

*Risk–delay interactions*. For mixed gambles (where both gains and losses are possible), delay should increase the premium paid to avoid potential losses, since the delay introduces disproportionately more disutility for losses. For risky choices about “pure” gains and losses, delay should actually *neutralize* risk relative to prospect theory predictions (i.e., make people less risk‐avoidant for gains and less risk‐seeking for losses) because the delay introduces another source of (dis)utility in the form of savoring (increasing the upside to a gamble in the gain zone and decreasing risk‐avoidance) and dread (increasing the downside to a gamble in the loss zone and decreasing risk‐seeking); the latter effect should be stronger than the former due to dread aversion.

*Individual differences*. The fact that people differ sharply in their anticipatory emotions leads to a rich variety of predictions to test about the magnitudes of intertemporal and risky choice phenomena. If the explanations above are correct, then dread‐averse (vs. “dread‐neutral”) people will (i) show a larger discounting asymmetry between gains and losses (the *sign effect*), (ii) have more nonlinear probability weighting functions (the *certainty effect*), but only for losses, (iii) be more present‐biased when losses are possible, (iv) be more loss‐averse but only for delayed prospects, and (v) have less elastic value functions (i.e., less concave for gains and less convex for losses) but only for delayed prospects.

*Consumption versus anticipatory utility functions*. Our formal model made very general assumptions about the shapes of the value, probability weighting, and discounting functions for consumption and anticipatory utility as our central goal was to show that a wide range of functional forms would lead to links between loss aversion, dread aversion, and risk and time preferences. Moreover, we showed via simulation (Appendix A of the Supporting Information) that our key theoretical results are not sensitive to these functional forms. Yet those simulations also revealed that other effects—such as the net role of the vividness of anticipatory emotions on risk and time preferences—do depend on such assumptions. Despite much work on elicitation of value, weighting, and discounting functions for consumption utility (e.g., Abdellaoui, 2000), little is known about these functions for anticipatory utility. Developing elicitation procedures to estimate these functions would be a valuable contribution.

*Predicted versus experienced emotions*. Disentangling anticipatory from reactive emotions leads to interesting questions about affective forecasting. For example, people overestimate the extent of loss aversion (Kermer et al., 2006) in the sense that they forecast, prior to a decision, that realized losses will be much more aversive than realized gains are pleasant, whereas the true asymmetry is smaller. Potentially, this could be due to participants’ factoring in anticipatory (dis)utility experienced *prior* to the outcome being revealed. Since dread is stronger than savoring—specifically in this instance that σ>1—expected anticipatory emotions enhance forecasts about losses more than for gains. If so, then affective forecasting errors should be larger for vivid outcomes or over longer delays. A related question is whether dread aversion *itself* might be an affective forecasting error. Since decisions, including risk and time preferences, depend on *expected* emotions rather than experienced emotions, our model does not require that expectations about emotions be rational. However, since neural evidence points to the aversive experience of dread (e.g., Berns et al., 2006), some degree of expected dread aversion is probably rational, even if it proves exaggerated in forecasts.

Finally, these results have potential practical value. In health contexts, our results suggest that dread‐averse people may be less willing to seek out information that is dread‐inducing, leading to less preventative care and thus higher long‐term costs—for example, would people with potential HIV (Human Immunodeficiency Virus) exposure be likelier to get tested if results were instantaneous rather than requiring 3 weeks (cf., Ganguly & Tasoff, 2017)? In marketing, dread aversion may help to explain the powerful effects of *promotion‐focus* (motivation to bring about good outcomes) versus *prevention‐focus* (motivation to avoid bad outcomes), which differ both across situations and individuals and have important consequences for the success of marketing appeals (Avnet & Higgins, 2006). In finance, dread aversion likely pushes investors toward safer investments, which is one potential explanation for the equity premium puzzle (Caplin & Leahy, 2001) and suggests that dread‐neutral investors can capitalize on this mispricing without the emotional cost taken by more dread‐averse investors. Likewise, in labor markets, more dread‐averse employees would favor payment structures with lower uncertainty. For strategic management and entrepreneurship, our results suggest that dread‐averse decision‐makers are likely to make more conservative decisions, which may be more or less desirable depending on the firm's industry, size, competitive positioning, and asset profile.

The anticipation of unpleasant events can have an equally, if not larger, impact on distress levels as the event itself (Berns et al., 2006; Lazarus, 1966) and, unlike the event itself, can be experienced continuously from the time of the decision until the event occurs. Therefore, asymmetries in dread and savoring may be a fundamental individual determinant of economic preferences on par with loss aversion.

## Supporting information



Supporting Information

## Notes

1 The reason we define asymmetric anticipatory emotions and asymmetric reactive emotions in terms of differences instead of ratios, at the individual level, is because the distributions of individual level dread and savoring for instance take both positive and negative values. Furthermore, using a ratio would produce unstable and potentially misleading values for individuals with scores near zero.

2 We investigated heterogeneous effects of asymmetric anticipatory emotions on economic preferences across age, sex, income, education, trait optimism, and trait pessimism but found no statistically significant effects. Similarly, we found no evidence of non‐linear effects of asymmetric anticipatory emotions on economic preferences.

3 For both risk and time preferences (Section 4.2.2.) we require a reliability, r<0.25, to rule out a statistically significant effect of asymmetric anticipatory emotions at the 5% level.
