# Supplementary material for: Asymmetric Anticipatory Emotions and Economic Preferences: Dread, Savoring, Risk, and Time
Source: Cogn Sci. 2026 Jan 20;50(1):e70160. doi: 10.1111/cogs.70160 (PMC12818390; doi:10.1111/cogs.70160)
Supplement: Supplementary file 1 — Supporting Information [file COGS-50-e70160-s001.docx]

**Supplementary Materials**

**For**

**“Asymmetric Anticipatory Emotions and Economic Preferences:**

**Dread, Savoring, Risk, and Time”**

**Appendix A**

Tables A1, A2, A3, A4, A5, A6, A7, and A8.

Figures A1 and A2

**Appendix B**Tables B1, B2, B3, B4, B5, B6, B7, B8, B9, B10, B11, B12, and B13.

Figures B1, B2, and B3

**Appendix A**

**Theoretical Simulations**

In the main text, we showed analytically that increasing dread aversion leads to greater risk-avoidance and impatience under a wide range of assumptions. Here, we use computer simulations to further test the robustness of the model to an even wider range of assumptions and also examine effects for cases where the analytical proof does not determine an unambiguous answer.

**Simulation 0: Illustrative Example**

We sketch the simulation strategy by starting with a simple example. First, we restrict ourselves to a choice context in which a decision-maker faces a mixed gamble: a 50% chance to win or lose $100, with the outcome of the gamble resolved after a delay of 3 periods. We assume for the moment that consumption utility is linear in (and equal to) money, anticipatory utility is linear in consumption utility (multiplied by $\alpha$), probability weights are equal to objective probabilities, there is no discounting, and attention is constant across the interval [$a\left( t \right)=1$]. These choice characteristics and functional assumptions match the numerical example in Table 2 of the main text, which is reproduced below as Table A1.

The first three columns indicate that we are parametrically varying $\alpha$ (vividness), $\lambda$ (loss aversion), and $\sigma$ (dread aversion). The next four columns indicate the magnitude of consumption and anticipatory utility for each component of the gamble considered separately. Finally, the last two columns are measures of predicted risk and time preferences related to the valuation of the gamble. The “delayed certainty equivalent” column holds constant time by calculating what certain monetary gain or loss would make the decision-maker indifferent between that certainty versus the gamble. Thus, lower values indicate greater risk-avoidance as the decision-maker is willing to pay more to avoid the gamble. The “present gamble equivalent” column holds constant probability by identifying a gamble to be resolved immediately that would make the decision-maker indifferent between that gamble and the delayed gamble; specifically, the column indicates the value of the gain in this gamble, where the value of the loss and the probabilities of gain and loss are the same as the delayed gamble. Thus, lower values indicate greater impatience, since the decision-maker is willing to accept a smaller upside to the gamble in order to resolve it immediately.

A simple way to quantify the effects of each of these variables is to regress our measures of risk and time preferences on each variable and their interactions. Here we are not interested in measures of statistical significance—since the “data” are the deterministic results of a mathematical model—but rather we are using the regressions as a way to determine the sign of each variable while holding the other variables constant.

**Table A1.** *Example of Varying* $\sigma$, $\lambda$, *and* $\alpha$ *to Examine Risk and Time Preferences*

| Assumptions | | | Utility | | | | Preferences | |
| --- | --- | --- | --- | --- | --- | --- | --- | --- |
| $\alpha$ | $\lambda$ | $\sigma$ | $U_{C}(gain)$ | $U_{C}( loss)$ | $U_{A}(gain)$ | $U_{A}(loss)$ | Delayed Certainty Equivalent | Present Gamble Equivalent |
| 0 | 1 | 1 | 50 | –50 | 0 | 0 | $0 | $100 |
| 0 | 2 | 1 | 50 | –100 | 0 | 0 | –$25 | $100 |
| 0 | 1 | 3 | 50 | –50 | 0 | 0 | $0 | $100 |
| 0 | 2 | 3 | 50 | –100 | 0 | 0 | –$25 | $100 |
| 0.05 | 1 | 1 | 50 | –50 | 7.5 | –7.5 | $0 | $100 |
| 0.05 | 2 | 1 | 50 | –100 | 7.5 | –15 | –$28.75 | $85 |
| 0.05 | 1 | 2 | 50 | –50 | 7.5 | –15 | –$7.50 | $85 |
| 0.05 | 2 | 2 | 50 | –100 | 7.5 | –30 | –$36.25 | $55 |

*Note*. Entries relate to a gamble to be resolved after 3 periods, with a 50% chance of gaining $100 and 50% chance of losing $100. This gamble is modeled under the assumptions that (i) anticipatory utility is either absent or accrues at 0.05 times consumption utility per period ($\alpha$ of 0 or 0.05); (ii) loss aversion is either absent or losses are twice as powerful as gains ($\lambda$ of 1 or 2); and (iii) dread aversion is either absent or dread is twice as powerful as savoring ($\sigma$ of 1 or 2). Consumption utility ($U_{C}$) and anticipatory utility ($U_{A}$) are calculated separately for the $100 gain and $100 loss after the 3 periods; see main text for the assumptions underlying these calculations (importantly, there is no discounting). The delayed certainty equivalent is the sure-thing payoff after 3 periods which would make the decision-maker indifferent between that payoff and the gamble; thus, negative values reflect risk-avoidance and positive values reflect risk-seeking. The present gamble equivalent is the value of the upside in an immediate gamble with a $100 downside, which would make the decision-maker equivalent between that gamble and the delayed gamble; thus, values less than $100 indicate greater impatience.

In the case of Table A2, we conduct a series of four regressions in order to demonstrate ten trends. The ten trends are given by the three main effect coefficients in each of Models 1a (for risk preferences) and 2a (for time preferences) and the two interaction coefficients in each of Models 1b (risk) and 2b (time). Note that the lower-order coefficients in Models 1b and 2b are not informative because these coefficients hold constant the values of the other parameters at 0 –including $\alpha$, where $\alpha=0$ indicates no anticipatory utility, and $\lambda$, where
$\lambda=0$ indicates that losses are not weighed at all (but gains are).

The main effects of $\sigma$ and $\lambda$ are exactly as expected based on the proofs in the main text and a glance at the table. Both dread aversion and loss aversion lead to greater risk-avoidance and impatience because greater anticipatory disutility due to dread (relative to the anticipatory utility due to savoring) makes delayed risks more unpleasant. In addition, there is a main effect of $\alpha$ wherein more vividly anticipating the future leads to greater risk-avoidance and impatience. As we will see, however, this main effect does *not* follow from the model in all cases, nor is it consistent across different choices of choice characteristics and functional assumptions. For example, if we had included multiple cases wherein $\sigma$ and $\lambda$ were less than 1, greater $\alpha$ would be associated with *less* risk-avoidance and impatience, since savoring would in the majority of cases outweigh dread.

**Table A2.** *Example Regression Models Using Data from Table A1.*

| Parameter | DV: Risk Preferences | | DV: Time Preferences | |
| --- | --- | --- | --- | --- |
|  | Model 1a | Model 1b | Model 2a | Model 2b |
| $\sigma$ | *–3.8* | 0.0 | *–11.3* | 0 |
| $\lambda$ | *–27.0* | –25.0 | *–11.3* | 0 |
| $\alpha$ | *–112.5* | 225.0 | *–375.0* | 975.0 |
| $\sigma*\alpha$ |  | *–150.0* |  | *-450.0* |
| $\lambda*\alpha$ |  | *–75.0* |  | *-450.0* |

*Note*. Entries are regression coefficients in models predicting delayed certainty equivalents and present gamble equivalents from $\sigma$, $\lambda$, $\alpha$, and their interactions. The coefficients in *underlined italics* are those we use in subsequent analyses; lower-order coefficients in Models 1b and 2b are uninformative.

To more formally assess this idea, we test for the interaction effects for $\sigma*\alpha$ and for
$\lambda*\alpha$ on both dependent variables. In all four cases, these coefficients too are negative. This indicates that the greater the value of $\alpha$, the larger the effect of dread aversion and loss aversion on risk-avoidance and impatience. In Table A1, for instance, dread aversion has no effect on either risk or time preferences when $\alpha=0$, but a dramatic effect when $\alpha=0.05$.

**Simulation 1: Varying Choice Characteristics**

Simulation 1 adds two layers of complexity: varying the choice characteristics and increasing the number of levels of $\sigma$, $\lambda$, and $\alpha$. The results form the baseline to which the results of subsequent simulations will be compared as all subsequent simulations also vary these characteristics.

We continue to assume that the decision-maker faces a mixed gamble with a possible gain and loss, to be resolved in the future, and for now we make the same assumptions as in the above illustrative example about the weighting, probability, discounting, and attention functions. However, we vary three choice characteristics. First, the delay can be short (1 period), moderate (3 periods), or long (10 periods). Second, the probability of gain can be small (.1), moderate (.5), or large (.9), where the probability of loss simply equals 1 minus the probability of gain. Third, the ratio of the expected value of the gain to loss can be unfavorable (.25), neutral (1), or favorable (4). The loss amount is always held constant at –100. For example, if the probability of gain is small (.1), the magnitude of the gain would be 225 when the gamble is unfavorable (an expected value of $-100*.9=-90$ for the loss and $225*.1=22.5$ for the gain, yielding a ratio of $\frac{22.5}{90}=.25$), 900 when the gamble is neutral (expected value of –90 for the loss and 90 for the gain, yielding a ratio of 1), and 3600 when the gamble is favorable (expected value of –90 for the loss and 360 for the gain, yielding a ratio of 4).

For each of the 27 sets of choice characteristics described above, we repeat the simulation and regression method described in Simulation 0 with a larger number of levels of $\sigma$, $\lambda$, and $\alpha$. Rather than using all combinations of 2 levels of each parameter, we instead use all combinations of 5 levels of $\sigma$ (1/3, 1/2, 1, 2, 3), $\lambda$(1/3, 1/2, 1, 2, 3), and $\alpha$ (0, 0.05, 0.10, 0.15, 0.20). These are reported in Table A3, simplifying the output by reporting the sign rather than magnitude of each regression coefficient (since the magnitudes are not necessarily on comparable scales across the sets of choice characteristics). Thus, each row represents the sign of a regression coefficient on 125 “observations” (for each combination of $\sigma$, $\lambda$, and $\alpha$ tested).

**Table A3.** *Detailed* *Results of Simulation 1*

| Gain/Loss Ratio | Delay | Probability of Gain | Signs of Regression Coefficients | | | | | | | | | | |
| --- | --- | --- | --- | --- | --- | --- | --- | --- | --- | --- | --- | --- | --- |
|  |  |  | Risk Preferences | | | | | Time Preferences | | | | | |
|  |  |  | $\sigma$ | $\lambda$ | $\alpha$ | $\sigma*\alpha$ | $\lambda*\alpha$ | $\sigma$ | $\lambda$ | $\alpha$ | $\sigma*\alpha$ | $\lambda*\alpha$ |  |
| .25 | 1 | .1 | – | – | – | – | – | – | – | – | – | – |  |
| 1 | 1 | .1 | – | – | – | – | – | – | – | – | – | – |  |
| 4 | 1 | .1 | – | – | + | – | – | – | – | + | – | – |  |
| .25 | 3 | .1 | – | – | – | – | – | – | – | – | – | – |  |
| 1 | 3 | .1 | – | – | – | – | – | – | – | – | – | – |  |
| 4 | 3 | .1 | – | – | + | – | – | – | – | + | – | – |  |
| .25 | 10 | .1 | – | – | – | – | – | – | – | – | – | – |  |
| 1 | 10 | .1 | – | – | – | – | – | – | – | – | – | – |  |
| 4 | 10 | .1 | – | – | + | – | – | – | – | + | – | – |  |
| .25 | 1 | .5 | – | – | – | – | – | – | – | – | – | – |  |
| 1 | 1 | .5 | – | – | – | – | – | – | – | – | – | – |  |
| 4 | 1 | .5 | – | – | + | – | – | – | – | + | – | – |  |
| .25 | 3 | .5 | – | – | – | – | – | – | – | – | – | – |  |
| 1 | 3 | .5 | – | – | – | – | – | – | – | – | – | – |  |
| 4 | 3 | .5 | – | – | + | – | – | – | – | + | – | – |  |
| .25 | 10 | .5 | – | – | – | – | – | – | – | – | – | – |  |
| 1 | 10 | .5 | – | – | – | – | – | – | – | – | – | – |  |
| 4 | 10 | .5 | – | – | + | – | – | – | – | + | – | – |  |
| .25 | 1 | .9 | – | – | – | – | – | – | – | – | – | – |  |
| 1 | 1 | .9 | – | – | – | – | – | – | – | – | – | – |  |
| 4 | 1 | .9 | – | – | + | – | – | – | – | + | – | – |  |
| .25 | 3 | .9 | – | – | – | – | – | – | – | – | – | – |  |
| 1 | 3 | .9 | – | – | – | – | – | – | – | – | – | – |  |
| 4 | 3 | .9 | – | – | + | – | – | – | – | + | – | – |  |
| .25 | 10 | .9 | – | – | – | – | – | – | – | – | – | – |  |
| 1 | 10 | .9 | – | – | – | – | – | – | – | – | – | – |  |
| 4 | 10 | .9 | – | – | + | – | – | – | – | + | – | – |  |

*Note*. Entries are the signs of regression coefficients in models with the same structure as those described in Simulation 0 (i.e., signs of the 10 coefficients presented in bold in Table A2, for each combination of choice characteristics). Negative entries correspond to lower delayed certainty equivalents (i.e., more risk-avoidance) and to lower present gamble equivalents (i.e., more impatience). For example, the row for a Gain/Loss ratio of 1, Delay of 3, and Probability of Gain of .5 is a summary of the four regressions in Table A2.

This table reveals several patterns. First, $\sigma$ always has a negative sign for both risk and time preferences, meaning that greater dread aversion yields more risk-avoidance and impatience regardless of the gain/loss ratio, delay, or probability of gain. This is unsurprising because we proved this result in the main text.

Second, $\lambda$ always has a negative sign for both risk preferences and time preferences. This aligns with the analysis in the main text, which showed that $\lambda$ has an unambiguous effect on risk-avoidance but an ambiguous effect on impatience. The reason for this ambiguity, however, is that discounting can create opportunities to reduce consumption (dis)utility by delaying losses, which can lead to a utility gain when loss aversion is high. In this simulation, there is no discounting and therefore this opposing effect cannot occur.

Third, $\alpha$ does not have a consistent effect on either risk or time preferences because a high enough gain/loss ratio can induce enough savoring for more vivid anticipatory emotions to yield greater net anticipatory utility from a mixed gamble.

Finally, the interaction effects are always negative, indicating that higher levels of $\alpha$ always exacerbate the induced risk-avoidance and impatience from $\sigma$ and $\lambda$. We did not examine this analytically in the main text, but it is consistent with the argument presented there.

To ease interpretation, we can also summarize this simulation by summarizing the number of negative coefficients based on each of the three choice characteristics, as in the top rows of Table A4, marked as *Sim 1*. This table—and subsequent summary tables for our other simulations—is best interpreted by seeing which parameters produce variation in the sign of the coefficient. For example, the sign of $\alpha$ (on both risk and time preferences) only varies as a function of the gain/loss ratio. All other effects are invariantly negative.

**Simulation 2: Varying Value Functions**

So far, we relied on very simple assumptions about the value, weighting, and discounting functions and also assumed that they are the same for consumption and anticipatory utility. In Simulations 2–5, we explore a wider range of functional forms and allow them to vary for each type of utility.

Simulation 2 examines the robustness of our theoretical results to different choices of value function. In these simulations, we allow the value function to be either linear (as in Simulation 1) or the prospect theory value function (Tversky & Kahneman, 1992), and we explore all combinations of these value functions for consumption and anticipatory utility.

Doing so requires a few additional assumptions. We retain the assumption of linear probability weighting and no discounting (for comparability to Simulation 1). For the functional form of the prospect theory value function, we use the formulation by Tversky and Kahneman (1992), in which $v\left( x \right)=x^{a}$ for gains ($x\geq0$) and $v\left( x \right)={-\lambda(-x)}^{b}$ for losses ($x<0$). Note that this allows the concavity of the value function to vary for gains and losses, and this tends to vary slightly in estimation studies (e.g., Abdellaoui, 2000). For simplicity, we set these parameters at $a=b=.9$, which are roughly in line with median empirical estimates.

The bottom panels of Table A5—Simulations 2A, 2B, and 2C—summarize the results of simulations in which, respectively, (i) consumption utility is given by prospect theory while anticipatory utility is linear in money, (ii) anticipatory utility is given by prospect theory while consumption utility is linear in money, and (iii) both consumption and anticipatory utility are given by prospect theory. These results can also be compared to Simulation 1 in the top quarter of the table, in which both consumption and anticipatory utility are linear.

**Table A4.** *Summary Results of Simulations 1 and 2 (Varying Value Functions)*

| Value Functions | Choice Characteristic | | Proportion of Negative Regression Coefficients | | | | | | | | | |
| --- | --- | --- | --- | --- | --- | --- | --- | --- | --- | --- | --- | --- |
|  |  |  | Risk Preferences | | | | | Time Preferences | | | | |
|  |  |  | $\sigma$ | $\lambda$ | $\alpha$ | $\sigma*\alpha$ | $\lambda*\alpha$ | $\sigma$ | $\lambda$ | $\alpha$ | $\sigma*\alpha$ | $\lambda*\alpha$ |
| *Sim 1*  Consumption:  Linear  Anticipation:  Linear | Gain/Loss Ratio | .25 | 1 | 1 | 1 | 1 | 1 | 1 | 1 | 1 | 1 | 1 |
|  |  | 1 | 1 | 1 | 1 | 1 | 1 | 1 | 1 | 1 | 1 | 1 |
|  |  | 4 | 1 | 1 | 0 | 1 | 1 | 1 | 1 | 0 | 1 | 1 |
|  | Delay | 1 | 1 | 1 | .67 | 1 | 1 | 1 | 1 | .67 | 1 | 1 |
|  |  | 3 | 1 | 1 | .67 | 1 | 1 | 1 | 1 | .67 | 1 | 1 |
|  |  | 10 | 1 | 1 | .67 | 1 | 1 | 1 | 1 | .67 | 1 | 1 |
|  | Probability of Gain | .1 | 1 | 1 | .67 | 1 | 1 | 1 | 1 | .67 | 1 | 1 |
|  |  | .5 | 1 | 1 | .67 | 1 | 1 | 1 | 1 | .67 | 1 | 1 |
|  |  | .9 | 1 | 1 | .67 | 1 | 1 | 1 | 1 | .67 | 1 | 1 |
| *Sim 2A*  Consumption:  Prospect  Anticipation:  Linear | Gain/Loss Ratio | .25 | 1 | 1 | 1 | 1 | 1 | 1 | 1 | 1 | 1 | 1 |
|  |  | 1 | 1 | 1 | 1 | 1 | 1 | 1 | 1 | 1 | 1 | 1 |
|  |  | 4 | 1 | 1 | 0 | 1 | 1 | 1 | 1 | 0 | 1 | 1 |
|  | Delay | 1 | 1 | 1 | .67 | 1 | 1 | 1 | 1 | .67 | 1 | 1 |
|  |  | 3 | 1 | 1 | .67 | 1 | 1 | 1 | 1 | .67 | 1 | 1 |
|  |  | 10 | 1 | 1 | .67 | 1 | 1 | 1 | 1 | .67 | 1 | 1 |
|  | Probability of Gain | .1 | 1 | 1 | .67 | 1 | 1 | 1 | 1 | .67 | 1 | 1 |
|  |  | .5 | 1 | 1 | .67 | 1 | 1 | 1 | 1 | .67 | 1 | 1 |
|  |  | .9 | 1 | 1 | .67 | 1 | 1 | 1 | 1 | .67 | 1 | 1 |
| *Sim 2B*  Consumption:  Linear  Anticipation:  Prospect | Gain/Loss Ratio | .25 | 1 | 1 | 0 | 1 | 1 | 1 | 1 | 0 | 1 | 1 |
|  |  | 1 | 1 | 1 | 0 | 1 | 1 | 1 | 1 | 0 | 1 | 1 |
|  |  | 4 | 1 | 1 | 0 | 1 | 1 | 1 | 1 | 0 | 1 | 1 |
|  | Delay | 1 | 1 | 1 | 0 | 1 | 1 | 1 | 1 | 0 | 1 | 1 |
|  |  | 3 | 1 | 1 | 0 | 1 | 1 | 1 | 1 | 0 | 1 | 1 |
|  |  | 10 | 1 | 1 | 0 | 1 | 1 | 1 | 1 | 0 | 1 | 1 |
|  | Probability of Gain | .1 | 1 | 1 | 0 | 1 | 1 | 1 | 1 | 0 | 1 | 1 |
|  |  | .5 | 1 | 1 | 0 | 1 | 1 | 1 | 1 | 0 | 1 | 1 |
|  |  | .9 | 1 | 1 | 0 | 1 | 1 | 1 | 1 | 0 | 1 | 1 |
| *Sim 2C*  Consumption:  Prospect  Anticipation:  Prospect | Gain/Loss Ratio | .25 | 1 | 1 | 0 | 1 | 1 | 1 | 1 | 0 | 1 | 1 |
|  |  | 1 | 1 | 1 | 0 | 1 | 1 | 1 | 1 | 0 | 1 | 1 |
|  |  | 4 | 1 | 1 | 0 | 1 | 1 | 1 | 1 | 0 | 1 | 1 |
|  | Delay | 1 | 1 | 1 | 0 | 1 | 1 | 1 | 1 | 0 | 1 | 1 |
|  |  | 3 | 1 | 1 | 0 | 1 | 1 | 1 | 1 | 0 | 1 | 1 |
|  |  | 10 | 1 | 1 | 0 | 1 | 1 | 1 | 1 | 0 | 1 | 1 |
|  | Probability of Gain | .1 | 1 | 1 | 0 | 1 | 1 | 1 | 1 | 0 | 1 | 1 |
|  |  | .5 | 1 | 1 | 0 | 1 | 1 | 1 | 1 | 0 | 1 | 1 |
|  |  | .9 | 1 | 1 | 0 | 1 | 1 | 1 | 1 | 0 | 1 | 1 |

*Note*. For the top panel of the table (Sim 1), entries are the proportion of negative coefficients in Table A3, summarized by each of the three levels of each choice characteristic. For the rest of the table (Sims 2A, 2B, and 2C), entries follow the same procedure but instead summarize the results of Simulation 2.

The most important finding is what does *not* change across these models. For both risk and time preferences, $\sigma$ and $\lambda$ always lead to greater risk-avoidance and impatience. This accords with the analytic results in the main text, which assumed only that the value function was increasing in money, not that it was linear. In addition, the interactions of each of those parameters with $\alpha$ (i.e., $\sigma*\alpha$ and $\lambda*\alpha$ for risk; $\sigma*\alpha$ for time) are consistently negative, with higher levels of $\alpha$ exacerbating the effects of $\sigma$ and $\lambda$ on risk and time preferences.

A further pattern is also apparent from Table A4, going beyond the analytic proof in the main text. When anticipatory utility follows the prospect theory value function (in Simulations 2B and 2C), $\alpha$ on its own (i.e., not in interaction with $\sigma$ or $\lambda$) no longer leads to greater risk-avoidance or impatience. This appears to be due to the prospect theory assumption of diminishing sensitivity to losses (i.e., a convex utility function for losses). The resulting diminishing sensitivity to dread results in less disutility to dread, analogous to the standard result in prospect theory that diminishing sensitivity to losses can induce risk-seeking for gambles in the loss zone.

**Simulation 3: Varying Probability Weighting Functions**

Next, we investigate the probability weighting function. We return to a linear value function and retain our assumption of no discounting. We consider two possible probability weighting functions for consumption utility, and three for anticipatory utility.

The first weighting function is the same linear function used in Simulations 1 and 2. The second weighting function is an implementation of the prospect theory weighting function. Specifically, we use the Lattimore et al. (1992) version of this weighting function because its two-parameter functional form allows for separation of the function’s elevation (i.e., extent of over- and underweighting) and curvature (i.e., sensitivity to discriminating intermediate probabilities). This function is given by $\pi\left( p \right)=\frac{{cp}^{d}}{{cp}^{d}+{(1-p)}^{d}}$, where $c$ is the elevation parameter and $d$ is the curvature parameter. Both curvature and especially elevation tend to vary for gains versus losses in parameter estimation studies, but here we simplify matters by assuming the median value of these parameters for gains from Abdellaoui (2000), namely $c=.65$ and $d=.60$. We do not vary these parameters here, but build the capacity into the simulation framework in anticipation that doing so would be valuable in future theoretical work.

The third weighting function (only for anticipatory utility) is a step function with $\pi\left( 0 \right)=0$ and $\pi\left( p \right)=1$ for $p>0$. This captures the idea that it is the *possibility* of an event that induces savoring and dread, rather than these emotions scaling with likelihood (see Loewenstein et al., 2001).

**Table A5.** *Summary Results of Simulations 1 and 3 (Varying Probability Weighting Functions)*

| Weighting Functions | Choice Characteristic | | Proportion of Negative Regression Coefficients | | | | | | | | | |
| --- | --- | --- | --- | --- | --- | --- | --- | --- | --- | --- | --- | --- |
|  |  |  | Risk Preferences | | | | | Time Preferences | | | | |
|  |  |  | $\sigma$ | $\lambda$ | $\alpha$ | $\sigma*\alpha$ | $\lambda*\alpha$ | $\sigma$ | $\lambda$ | $\alpha$ | $\sigma*\alpha$ | $\lambda*\alpha$ |
| *Sim 1*  Consumption:  Linear  Anticipation:  Linear | Gain/Loss Ratio | .25 | 1 | 1 | 1 | 1 | 1 | 1 | 1 | 1 | 1 | 1 |
|  |  | 1 | 1 | 1 | 1 | 1 | 1 | 1 | 1 | 1 | 1 | 1 |
|  |  | 4 | 1 | 1 | 0 | 1 | 1 | 1 | 1 | 0 | 1 | 1 |
|  | Delay | 1 | 1 | 1 | .67 | 1 | 1 | 1 | 1 | .67 | 1 | 1 |
|  |  | 3 | 1 | 1 | .67 | 1 | 1 | 1 | 1 | .67 | 1 | 1 |
|  |  | 10 | 1 | 1 | .67 | 1 | 1 | 1 | 1 | .67 | 1 | 1 |
|  | Probability of Gain | .1 | 1 | 1 | .67 | 1 | 1 | 1 | 1 | .67 | 1 | 1 |
|  |  | .5 | 1 | 1 | .67 | 1 | 1 | 1 | 1 | .67 | 1 | 1 |
|  |  | .9 | 1 | 1 | .67 | 1 | 1 | 1 | 1 | .67 | 1 | 1 |
| *Sim 3A*  Consumption:  Prospect  Anticipation:  Linear | Gain/Loss Ratio | .25 | 1 | 1 | 1 | 1 | 1 | 1 | 1 | 1 | 1 | 1 |
|  |  | 1 | 1 | 1 | 1 | 1 | 1 | 1 | 1 | 1 | 1 | 1 |
|  |  | 4 | 1 | 1 | 0 | 1 | 1 | 1 | 1 | 0 | 1 | 1 |
|  | Delay | 1 | 1 | 1 | .67 | 1 | 1 | 1 | 1 | .67 | 1 | 1 |
|  |  | 3 | 1 | 1 | .67 | 1 | 1 | 1 | 1 | .67 | 1 | 1 |
|  |  | 10 | 1 | 1 | .67 | 1 | 1 | 1 | 1 | .67 | 1 | 1 |
|  | Probability of Gain | .1 | 1 | 1 | .67 | 1 | 1 | 1 | 1 | .67 | 1 | 1 |
|  |  | .5 | 1 | 1 | .67 | 1 | 1 | 1 | 1 | .67 | 1 | 1 |
|  |  | .9 | 1 | 1 | .67 | 1 | 1 | 1 | 1 | .67 | 1 | 1 |
| *Sim 3B*  Consumption:  Linear  Anticipation:  Prospect | Gain/Loss Ratio | .25 | 1 | 1 | 1 | 1 | 1 | 1 | 1 | 1 | 1 | 1 |
|  |  | 1 | 1 | 1 | .67 | 1 | 1 | 1 | 1 | .67 | 1 | 1 |
|  |  | 4 | 1 | 1 | 0 | 1 | 1 | 1 | 1 | 0 | 1 | 1 |
|  | Delay | 1 | 1 | 1 | .56 | 1 | 1 | 1 | 1 | .56 | 1 | 1 |
|  |  | 3 | 1 | 1 | .56 | 1 | 1 | 1 | 1 | .56 | 1 | 1 |
|  |  | 10 | 1 | 1 | .56 | 1 | 1 | 1 | 1 | .56 | 1 | 1 |
|  | Probability of Gain | .1 | 1 | 1 | .33 | 1 | 1 | 1 | 1 | .33 | 1 | 1 |
|  |  | .5 | 1 | 1 | .67 | 1 | 1 | 1 | 1 | .67 | 1 | 1 |
|  |  | .9 | 1 | 1 | .67 | 1 | 1 | 1 | 1 | .67 | 1 | 1 |
| *Sim 3C*  Consumption:  Prospect  Anticipation:  Prospect | Gain/Loss Ratio | .25 | 1 | 1 | 1 | 1 | 1 | 1 | 1 | 1 | 1 | 1 |
|  |  | 1 | 1 | 1 | .67 | 1 | 1 | 1 | 1 | .67 | 1 | 1 |
|  |  | 4 | 1 | 1 | 0 | 1 | 1 | 1 | 1 | 0 | 1 | 1 |
|  | Delay | 1 | 1 | 1 | .56 | 1 | 1 | 1 | 1 | .56 | 1 | 1 |
|  |  | 3 | 1 | 1 | .56 | 1 | 1 | 1 | 1 | .56 | 1 | 1 |
|  |  | 10 | 1 | 1 | .56 | 1 | 1 | 1 | 1 | .56 | 1 | 1 |
|  | Probability of Gain | .1 | 1 | 1 | .33 | 1 | 1 | 1 | 1 | .33 | 1 | 1 |
|  |  | .5 | 1 | 1 | .67 | 1 | 1 | 1 | 1 | .67 | 1 | 1 |
|  |  | .9 | 1 | 1 | .67 | 1 | 1 | 1 | 1 | .67 | 1 | 1 |
| *Sim 3D*  Consumption:  Linear  Anticipation:  Step | Gain/Loss Ratio | .25 | 1 | 1 | .67 | 1 | 1 | 1 | 1 | .67 | 1 | 1 |
|  |  | 1 | 1 | 1 | .67 | 1 | 1 | 1 | 1 | .67 | 1 | 1 |
|  |  | 4 | 1 | 1 | .33 | 1 | 1 | 1 | 1 | .33 | 1 | 1 |
|  | Delay | 1 | 1 | 1 | .56 | 1 | 1 | 1 | 1 | .56 | 1 | 1 |
|  |  | 3 | 1 | 1 | .56 | 1 | 1 | 1 | 1 | .56 | 1 | 1 |
|  |  | 10 | 1 | 1 | .56 | 1 | 1 | 1 | 1 | .56 | 1 | 1 |
|  | Probability of Gain | .1 | 1 | 1 | 0 | 1 | 1 | 1 | 1 | 0 | 1 | 1 |
|  |  | .5 | 1 | 1 | .67 | 1 | 1 | 1 | 1 | .67 | 1 | 1 |
|  |  | .9 | 1 | 1 | 1 | 1 | 1 | 1 | 1 | 1 | 1 | 1 |
| *Sim 3E*  Consumption:  Prospect  Anticipation:  Step | Gain/Loss Ratio | .25 | 1 | 1 | .67 | 1 | 1 | 1 | 1 | .67 | 1 | 1 |
|  |  | 1 | 1 | 1 | .67 | 1 | 1 | 1 | 1 | .67 | 1 | 1 |
|  |  | 4 | 1 | 1 | .33 | 1 | 1 | 1 | 1 | .33 | 1 | 1 |
|  | Delay | 1 | 1 | 1 | .56 | 1 | 1 | 1 | 1 | .56 | 1 | 1 |
|  |  | 3 | 1 | 1 | .56 | 1 | 1 | 1 | 1 | .56 | 1 | 1 |
|  |  | 10 | 1 | 1 | .56 | 1 | 1 | 1 | 1 | .56 | 1 | 1 |
|  | Probability of Gain | .1 | 1 | 1 | 0 | 1 | 1 | 1 | 1 | 0 | 1 | 1 |
|  |  | .5 | 1 | 1 | .67 | 1 | 1 | 1 | 1 | .67 | 1 | 1 |
|  |  | .9 | 1 | 1 | 1 | 1 | 1 | 1 | 1 | 1 | 1 | 1 |

*Note*. The top panel of the table (Sim 1) is identical to the top panel of Table A4. The rest of the table (Sims 3A–E) reports the results of Simulation 3, using the same summarization method as Table A4.

This produces six sets of simulations (crossing two possible weighting functions for consumption by three for anticipation), which are summarized in Table A5. As in the analysis of different value functions, the coefficients for $\sigma$, $\lambda$, $\sigma*\alpha$, and $\lambda*\alpha$ are always consistent for risk-avoidance and impatience.

The effect of $\alpha$ on risk and time preferences, however, varied across simulations. The simulations can be examined in pairs that share the same anticipatory weighting function but vary in their consumption weighting function—1 and 3A, 3B and 3C, and 3D and 3E. Within each pair, the results are nearly identical, showing that it is the weighting function for anticipatory utility, rather than consumption utility, that produces variation. We focus on Simulations 1, 3B, and 3D—with linear probability weighting for consumption but varying in the weighting function for anticipatory utility—to examine patterns. The table is easiest to read by focusing on the three rows varying “probability of gain” within each of those three simulations.

For both $\alpha$ coefficients, making the weighting function for anticipatory utility increasingly steep for low probabilities (linear to prospect theory to step functions) leads to a sharper differentiation across the three probability levels. For example, $\alpha$ is half as likely to increase both risk-avoidance and impatience when the probability of gain is .1 in the prospect theory versus linear weighting function. This is because the .1 probability of gain is overweighted and the .9 probability of loss is underweighted, reducing the amount of dread relative to savoring and therefore making more vivid anticipatory emotions less aversive. This effect is even sharper with the step function, where all probability levels are treated the same for anticipatory emotions. This results in a severe overweighting of savoring (when the probability of gain = .1) and dread (when the probability of gain = .9), amplifying the effect described under the prospect theory weighting function.

Overall, the analytic results from the main text hold up again without exception, while the effect of $\alpha$ produces more interesting variance, particularly as we alter the weighting function for anticipatory utility.

**Simulation 4: Varying Discount Rates**

We next consider the impact of different discount rates for consumption and anticipatory utility. We use an exponential discounting function in Simulation 4, but consider the potential effects of varying the shape of this function in Simulation 5. Specifically, Simulation 4 used a discount factor $\delta\left( t \right)=-e^{r_{1}t}$, where $r_{1}$ is the discount rate. Discount rates tend to vary between gains and losses (the *sign effect*; Thaler, 1981), but this is at least partly due to anticipatory emotions (Hardisty & Weber, 2020)—specifically dread outweighing savoring—rather than to pure time preference. For the purpose of the present simulations, we assume discount rates are the same for gains and losses.


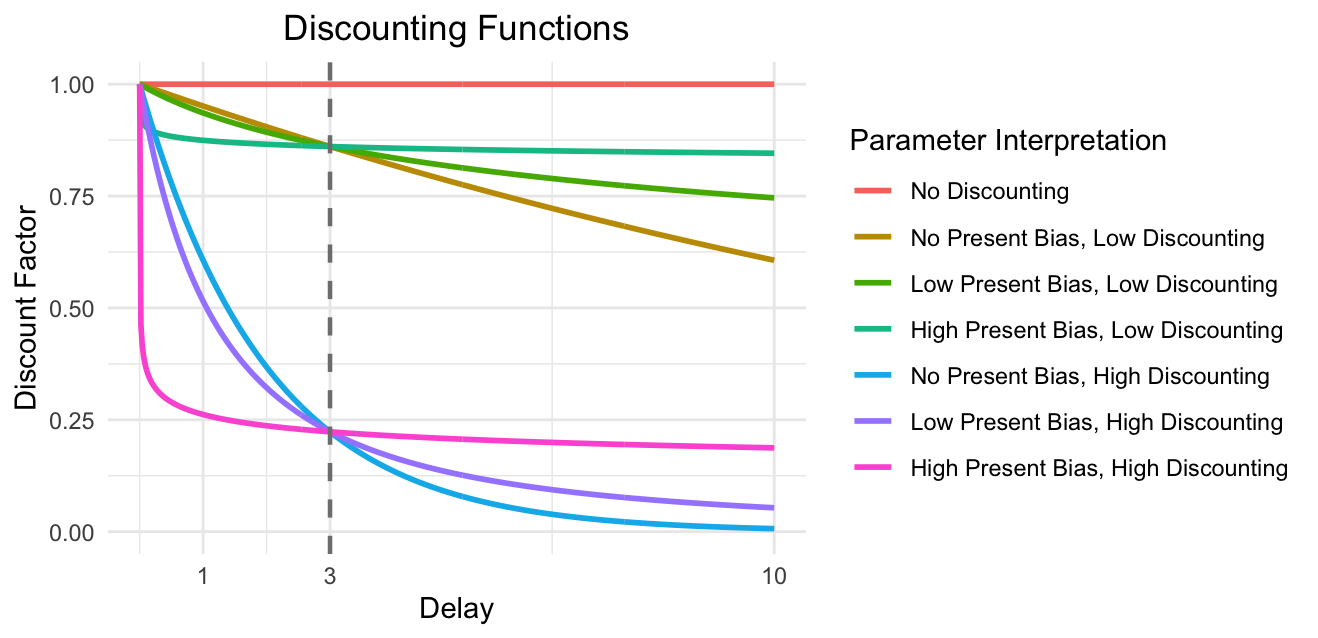


**Figure A1.** Discounting Functions Used in Simulations 4 and 5.

We selected discount rates of 0, .05, and .50. Estimates of discount rates vary widely in the literature (e.g., Yao et al., 2012). However, these rates should vary sufficiently to get an intuition for how the model behaves over different timeframes and rates. For example, if one interpreted one period as one year, then these rates would be roughly equivalent to demanding an annual interest rate of 0%, 5%, or 50% to accept a delayed payment. Alternatively, if one interpreted one period as one *month*, then these discount rates would be much steeper on an annualized basis (roughly 0%, 80%, and 13,000% annualized). It seems reasonable to say, therefore, that these discount rates span the full range of plausible values. These three exponential discounting functions are plotted in Figure A1 as “No Discounting” (0%), “No Present Bias, Low Discounting” (5%), and “No Present Bias, High Discounting” (50%).

Table A6 shows the results. Even across this very wide range of discount rates, the main results do not change, and in fact all parameter estimates are exactly the same as in Simulation 1 with the exception of the effect of $\lambda$ on time preferences. When consumption utility has a very high discount rate (50% in Simulations 4B and 4F), $\lambda$ is much less likely to induce impatience compared to all other simulations—indeed, these are the only cases we have seen so far where $\lambda$ does not have a uniform impatience-inducing effect. In Simulation 4B, this occurs especially for more unfavorable gain/loss ratios (e.g., .25 vs 4) and shorter delays (e.g., 1 vs 10 periods). The moderating role of delay makes sense here: At longer delays, the loss would have already been discounted so much that pushing it further into the future may not increase consumption utility very much. In Simulation 4F—where anticipatory utility is also discounted at a high (50%) rate— $\lambda$ never is associated with impatience. This also makes sense: When anticipatory utility is highly discounted, this reduces the relative importance of anticipation vs consumption utility in the model, so the delay-inducing effect of loss aversion via consumption utility prevails. **Table A6.** *Summary Results of Simulations 1 and 4 (Varying Discount Rates)*

| Value Functions | Choice Characteristic | | Proportion of Negative Regression Coefficients | | | | | | | | | | | | |
| --- | --- | --- | --- | --- | --- | --- | --- | --- | --- | --- | --- | --- | --- | --- | --- |
|  |  |  | Risk Preferences | | | | | | | | Time Preferences | | | | |
|  |  |  | $\sigma$ | | $\lambda$ | | $\alpha$ | | $\sigma*\alpha$ | $\lambda*\alpha$ | $\sigma$ | $\lambda$ | $\alpha$ | $\sigma*\alpha$ | $\lambda*\alpha$ |
| *Sim 1*  Consumption:  0% Rate  Anticipation:  0% Rate | Gain/Loss Ratio | .25 | 1 | 1 | | 1 | | 1 | | 1 | 1 | 1 | 1 | 1 | 1 |
|  |  | 1 | 1 | 1 | | 1 | | 1 | | 1 | 1 | 1 | 1 | 1 | 1 |
|  |  | 4 | 1 | 1 | | 0 | | 1 | | 1 | 1 | 1 | 0 | 1 | 1 |
|  | Delay | 1 | 1 | 1 | | .67 | | 1 | | 1 | 1 | 1 | .67 | 1 | 1 |
|  |  | 3 | 1 | 1 | | .67 | | 1 | | 1 | 1 | 1 | .67 | 1 | 1 |
|  |  | 10 | 1 | 1 | | .67 | | 1 | | 1 | 1 | 1 | .67 | 1 | 1 |
|  | Probability of Gain | .1 | 1 | 1 | | .67 | | 1 | | 1 | 1 | 1 | .67 | 1 | 1 |
|  |  | .5 | 1 | 1 | | .67 | | 1 | | 1 | 1 | 1 | .67 | 1 | 1 |
|  |  | .9 | 1 | 1 | | .67 | | 1 | | 1 | 1 | 1 | .67 | 1 | 1 |
| *Sim 4A*  Consumption:  5% Rate  Anticipation:  0% Rate | Gain/Loss Ratio | .25 | 1 | 1 | | 1 | | 1 | | 1 | 1 | 1 | 1 | 1 | 1 |
|  |  | 1 | 1 | 1 | | 1 | | 1 | | 1 | 1 | 1 | 1 | 1 | 1 |
|  |  | 4 | 1 | 1 | | 0 | | 1 | | 1 | 1 | 1 | 0 | 1 | 1 |
|  | Delay | 1 | 1 | 1 | | .67 | | 1 | | 1 | 1 | 1 | .67 | 1 | 1 |
|  |  | 3 | 1 | 1 | | .67 | | 1 | | 1 | 1 | 1 | .67 | 1 | 1 |
|  |  | 10 | 1 | 1 | | .67 | | 1 | | 1 | 1 | 1 | .67 | 1 | 1 |
|  | Probability of Gain | .1 | 1 | 1 | | .67 | | 1 | | 1 | 1 | 1 | .67 | 1 | 1 |
|  |  | .5 | 1 | 1 | | .67 | | 1 | | 1 | 1 | 1 | .67 | 1 | 1 |
|  |  | .9 | 1 | 1 | | .67 | | 1 | | 1 | 1 | 1 | .67 | 1 | 1 |
| *Sim 4B*  Consumption:  50% Rate  Anticipation:  0% Rate | Gain/Loss Ratio | .25 | 1 | 1 | | 1 | | 1 | | 1 | 1 | 0 | 1 | 1 | 1 |
|  |  | 1 | 1 | 1 | | 1 | | 1 | | 1 | 1 | 0 | 1 | 1 | 1 |
|  |  | 4 | 1 | 1 | | 0 | | 1 | | 1 | 1 | .33 | 0 | 1 | 1 |
|  | Delay | 1 | 1 | 1 | | .67 | | 1 | | 1 | 1 | 0 | .67 | 1 | 1 |
|  |  | 3 | 1 | 1 | | .67 | | 1 | | 1 | 1 | 0 | .67 | 1 | 1 |
|  |  | 10 | 1 | 1 | | .67 | | 1 | | 1 | 1 | .33 | .67 | 1 | 1 |
|  | Probability of Gain | .1 | 1 | 1 | | .67 | | 1 | | 1 | 1 | .11 | .67 | 1 | 1 |
|  |  | .5 | 1 | 1 | | .67 | | 1 | | 1 | 1 | .11 | .67 | 1 | 1 |
|  |  | .9 | 1 | 1 | | .67 | | 1 | | 1 | 1 | .11 | .67 | 1 | 1 |
| *Sim 4C*  Consumption:  0% Rate  Anticipation:  5% Rate | Gain/Loss Ratio | .25 | 1 | 1 | | 1 | | 1 | | 1 | 1 | 1 | 1 | 1 | 1 |
|  |  | 1 | 1 | 1 | | 1 | | 1 | | 1 | 1 | 1 | 1 | 1 | 1 |
|  |  | 4 | 1 | 1 | | 0 | | 1 | | 1 | 1 | 1 | 0 | 1 | 1 |
|  | Delay | 1 | 1 | 1 | | .67 | | 1 | | 1 | 1 | 1 | .67 | 1 | 1 |
|  |  | 3 | 1 | 1 | | .67 | | 1 | | 1 | 1 | 1 | .67 | 1 | 1 |
|  |  | 10 | 1 | 1 | | .67 | | 1 | | 1 | 1 | 1 | .67 | 1 | 1 |
|  | Probability of Gain | .1 | 1 | 1 | | .67 | | 1 | | 1 | 1 | 1 | .67 | 1 | 1 |
|  |  | .5 | 1 | 1 | | .67 | | 1 | | 1 | 1 | 1 | .67 | 1 | 1 |
|  |  | .9 | 1 | 1 | | .67 | | 1 | | 1 | 1 | 1 | .67 | 1 | 1 |
| *Sim 4D*  Consumption:  0% Rate  Anticipation:  50% Rate | Gain/Loss Ratio | .25 | 1 | 1 | | 1 | | 1 | | 1 | 1 | 1 | 1 | 1 | 1 |
|  |  | 1 | 1 | 1 | | 1 | | 1 | | 1 | 1 | 1 | 1 | 1 | 1 |
|  |  | 4 | 1 | 1 | | 0 | | 1 | | 1 | 1 | 1 | 0 | 1 | 1 |
|  | Delay | 1 | 1 | 1 | | .67 | | 1 | | 1 | 1 | 1 | .67 | 1 | 1 |
|  |  | 3 | 1 | 1 | | .67 | | 1 | | 1 | 1 | 1 | .67 | 1 | 1 |
|  |  | 10 | 1 | 1 | | .67 | | 1 | | 1 | 1 | 1 | .67 | 1 | 1 |
|  | Probability of Gain | .1 | 1 | 1 | | .67 | | 1 | | 1 | 1 | 1 | .67 | 1 | 1 |
|  |  | .5 | 1 | 1 | | .67 | | 1 | | 1 | 1 | 1 | .67 | 1 | 1 |
|  |  | .9 | 1 | 1 | | .67 | | 1 | | 1 | 1 | 1 | .67 | 1 | 1 |
| *Sim 4E*  Consumption:  5% Rate  Anticipation:  5% Rate | Gain/Loss Ratio | .25 | 1 | 1 | | 1 | | 1 | | 1 | 1 | 1 | 1 | 1 | 1 |
|  |  | 1 | 1 | 1 | | 1 | | 1 | | 1 | 1 | 1 | 1 | 1 | 1 |
|  |  | 4 | 1 | 1 | | 0 | | 1 | | 1 | 1 | 1 | 0 | 1 | 1 |
|  | Delay | 1 | 1 | 1 | | .67 | | 1 | | 1 | 1 | 1 | .67 | 1 | 1 |
|  |  | 3 | 1 | 1 | | .67 | | 1 | | 1 | 1 | 1 | .67 | 1 | 1 |
|  |  | 10 | 1 | 1 | | .67 | | 1 | | 1 | 1 | 1 | .67 | 1 | 1 |
|  | Probability of Gain | .1 | 1 | 1 | | .67 | | 1 | | 1 | 1 | 1 | .67 | 1 | 1 |
|  |  | .5 | 1 | 1 | | .67 | | 1 | | 1 | 1 | 1 | .67 | 1 | 1 |
|  |  | .9 | 1 | 1 | | .67 | | 1 | | 1 | 1 | 1 | .67 | 1 | 1 |
| *Sim 4F*  Consumption:  50% Rate  Anticipation:  50% Rate | Gain/Loss Ratio | .25 | 1 | 1 | | 1 | | 1 | | 1 | 1 | 0 | 1 | 1 | 1 |
|  |  | 1 | 1 | 1 | | 1 | | 1 | | 1 | 1 | 0 | 1 | 1 | 1 |
|  |  | 4 | 1 | 1 | | 0 | | 1 | | 1 | 1 | 0 | 0 | 1 | 1 |
|  | Delay | 1 | 1 | 1 | | .67 | | 1 | | 1 | 1 | 0 | .67 | 1 | 1 |
|  |  | 3 | 1 | 1 | | .67 | | 1 | | 1 | 1 | 0 | .67 | 1 | 1 |
|  |  | 10 | 1 | 1 | | .67 | | 1 | | 1 | 1 | 0 | .67 | 1 | 1 |
|  | Probability of Gain | .1 | 1 | 1 | | .67 | | 1 | | 1 | 1 | 0 | .67 | 1 | 1 |
|  |  | .5 | 1 | 1 | | .67 | | 1 | | 1 | 1 | 0 | .67 | 1 | 1 |
|  |  | .9 | 1 | 1 | | .67 | | 1 | | 1 | 1 | 0 | .67 | 1 | 1 |

*Note*. The top panel of the table (Sim 1) is identical to the top panel of Tables A4 and A5. The rest of the table (Sims 4A–F) reports the results of Simulation 4, using the same summarization method as Tables A4 and A5.

**Simulation 5: Varying Present Bias**

Although the overall *rate* of discounting affects the model’s behavior, we assumed an exponential discounting function which does not account for the much steeper discounting in the near future than over the distant future. To test whether present bias impacts any of the results, we switch from an exponential to a hyperbolic discounting function, specifically that of Prelec and Loewenstein (1992), with a discount factor of $\delta\left( t \right)={(1+r_{2}t)}^{-r_{3}/r_{2}}$. We chose this formulation because this function approaches exponential discounting (with rate $r_{3}$) as $r_{2}$ goes toward 0, facilitating comparison with the results of Simulation 4.

To select values of $r_{2}$ and $r_{3}$, we set values that yield the same discount factor after 3 periods as 5% and 50% exponential discount rates. This allows us to see the impact of present bias both during the earlier period (when the hyperbolic discount factor is higher than its exponential cousin, at period 1) and later period (when it is lower, at period 10). Specifically, we set $r_{2}$ to either a modest degree of present bias ($r_{2}=5$) or a high degree ($r_{2}=10,000$), and then adjusted $r_{3}$ to a value that approximately equated the discount factor after 3 periods relative to either a 5% or 50% discount rate; this allowed us to orthogonally vary discount rate (the overall level of discounting) and present bias (the bias toward discounting earlier time periods more than later ones).

Specifically, for low discounting (i.e., a discount factor of 0.86 after 3 periods), we set $r_{3}=0.0818$ when present bias was low and $r_{3}=145.5$ when present bias was high. For high discounting (i.e., a discount factor of 0.22 after 3 periods), we set $r_{3}=0.818$ when present bias was low and $r_{3}=1455$ when present bias was high. The resulting four curves are plotted in Figure A1 as the four “Low Present Bias” and “High Present Bias” curves, with either “Low Discounting” or “High Discounting.”

Simulations 5A–D in Table A7 test these four discounting functions for consumption utility (assuming no discounting of anticipatory utility), showing that, as for varying discount rates, only the effect of $\lambda$ on time preferences changes across discounting functions. Although varying the discount rate makes a larger difference than varying present bias, introducing high present bias makes $\lambda$ less likely to produce impatience at short time horizons. This makes sense: Present bias simply means that discount rates are larger at shorter time horizons, so if larger discount rates reduce the impatience-inducing effect of $\lambda$, this should be more true at shorter time horizons with greater present bias.

Simulations 5E–H repeat the same exercise, this time with the discounting functions varying for anticipatory utility instead of consumption utility. As in Simulation 4, however, varying this discounting function in the absence of discounting for consumption utility had no impact on any result. In particular, discounting for anticipatory utility would not create a consumption utility improvement for delaying losses, so $\lambda$ purely increases impatience in these simulations.

**Table A7.** *Summary Results of Simulation 5 (Varying Present Bias and Discount Rate)*

| Value Functions | Choice Characteristic | | | Proportion of Negative Regression Coefficients | | | | | | | | | | | | |
| --- | --- | --- | --- | --- | --- | --- | --- | --- | --- | --- | --- | --- | --- | --- | --- | --- |
|  |  |  |  | Risk Preferences | | | | | | | | Time Preferences | | | | |
|  |  |  |  | $\sigma$ | | $\lambda$ | | $\alpha$ | | $\sigma*\alpha$ | $\lambda*\alpha$ | $\sigma$ | $\lambda$ | $\alpha$ | $\sigma*\alpha$ | $\lambda*\alpha$ |
| *Sim 5A*  Consumption:  Low Pres Bias  Low Rate  Anticipation:  None | Gain/Loss Ratio | .25 | 1 | | 1 | | 1 | | 1 | | 1 | 1 | 1 | 1 | 1 | 1 |
|  |  | 1 | 1 | | 1 | | 1 | | 1 | | 1 | 1 | 1 | 1 | 1 | 1 |
|  |  | 4 | 1 | | 1 | | 0 | | 1 | | 1 | 1 | 1 | 0 | 1 | 1 |
|  | Delay | 1 | 1 | | 1 | | .67 | | 1 | | 1 | 1 | 1 | .67 | 1 | 1 |
|  |  | 3 | 1 | | 1 | | .67 | | 1 | | 1 | 1 | 1 | .67 | 1 | 1 |
|  |  | 10 | 1 | | 1 | | .67 | | 1 | | 1 | 1 | 1 | .67 | 1 | 1 |
|  | Probability of Gain | .1 | 1 | | 1 | | .67 | | 1 | | 1 | 1 | 1 | .67 | 1 | 1 |
|  |  | .5 | 1 | | 1 | | .67 | | 1 | | 1 | 1 | 1 | .67 | 1 | 1 |
|  |  | .9 | 1 | | 1 | | .67 | | 1 | | 1 | 1 | 1 | .67 | 1 | 1 |
| *Sim 5B*  Consumption:  High Pres Bias  Low Rate  Anticipation:  None | Gain/Loss Ratio | .25 | 1 | | 1 | | 1 | | 1 | | 1 | 1 | .67 | 1 | 1 | 1 |
|  |  | 1 | 1 | | 1 | | 1 | | 1 | | 1 | 1 | 1 | 1 | 1 | 1 |
|  |  | 4 | 1 | | 1 | | 0 | | 1 | | 1 | 1 | 1 | 0 | 1 | 1 |
|  | Delay | 1 | 1 | | 1 | | .67 | | 1 | | 1 | 1 | .67 | .67 | 1 | 1 |
|  |  | 3 | 1 | | 1 | | .67 | | 1 | | 1 | 1 | 1 | .67 | 1 | 1 |
|  |  | 10 | 1 | | 1 | | .67 | | 1 | | 1 | 1 | 1 | .67 | 1 | 1 |
|  | Probability of Gain | .1 | 1 | | 1 | | .67 | | 1 | | 1 | 1 | .89 | .67 | 1 | 1 |
|  |  | .5 | 1 | | 1 | | .67 | | 1 | | 1 | 1 | .89 | .67 | 1 | 1 |
|  |  | .9 | 1 | | 1 | | .67 | | 1 | | 1 | 1 | .89 | .67 | 1 | 1 |
| *Sim 5C*  Consumption:  Low Pres Bias  High Rate  Anticipation:  None | Gain/Loss Ratio | .25 | 1 | | 1 | | 1 | | 1 | | 1 | 1 | 0 | 1 | 1 | 1 |
|  |  | 1 | 1 | | 1 | | 1 | | 1 | | 1 | 1 | 0 | 1 | 1 | 1 |
|  |  | 4 | 1 | | 1 | | 0 | | 1 | | 1 | 1 | .33 | 0 | 1 | 1 |
|  | Delay | 1 | 1 | | 1 | | .67 | | 1 | | 1 | 1 | 0 | .67 | 1 | 1 |
|  |  | 3 | 1 | | 1 | | .67 | | 1 | | 1 | 1 | 0 | .67 | 1 | 1 |
|  |  | 10 | 1 | | 1 | | .67 | | 1 | | 1 | 1 | .33 | .67 | 1 | 1 |
|  | Probability of Gain | .1 | 1 | | 1 | | .67 | | 1 | | 1 | 1 | .11 | .67 | 1 | 1 |
|  |  | .5 | 1 | | 1 | | .67 | | 1 | | 1 | 1 | .11 | .67 | 1 | 1 |
|  |  | .9 | 1 | | 1 | | .67 | | 1 | | 1 | 1 | .11 | .67 | 1 | 1 |
| *Sim 5D*  Consumption:  High Pres Bias  High Rate  Anticipation:  None | Gain/Loss Ratio | .25 | 1 | | 1 | | 1 | | 1 | | 1 | 1 | 0 | 1 | 1 | 1 |
|  |  | 1 | 1 | | 1 | | 1 | | 1 | | 1 | 1 | .33 | 1 | 1 | 1 |
|  |  | 4 | 1 | | 1 | | 0 | | 1 | | 1 | 1 | .33 | 0 | 1 | 1 |
|  | Delay | 1 | 1 | | 1 | | .67 | | 1 | | 1 | 1 | 0 | .67 | 1 | 1 |
|  |  | 3 | 1 | | 1 | | .67 | | 1 | | 1 | 1 | 0 | .67 | 1 | 1 |
|  |  | 10 | 1 | | 1 | | .67 | | 1 | | 1 | 1 | .67 | .67 | 1 | 1 |
|  | Probability of Gain | .1 | 1 | | 1 | | .67 | | 1 | | 1 | 1 | .22 | .67 | 1 | 1 |
|  |  | .5 | 1 | | 1 | | .67 | | 1 | | 1 | 1 | .22 | .67 | 1 | 1 |
|  |  | .9 | 1 | | 1 | | .67 | | 1 | | 1 | 1 | .22 | .67 | 1 | 1 |
| *Sim 5E*  Consumption:  None  Anticipation:  Low Pres Bias  Low Rate | Gain/Loss Ratio | .25 | 1 | | 1 | | 1 | | 1 | | 1 | 1 | 1 | 1 | 1 | 1 |
|  |  | 1 | 1 | | 1 | | 1 | | 1 | | 1 | 1 | 1 | 1 | 1 | 1 |
|  |  | 4 | 1 | | 1 | | 0 | | 1 | | 1 | 1 | 1 | 0 | 1 | 1 |
|  | Delay | 1 | 1 | | 1 | | .67 | | 1 | | 1 | 1 | 1 | .67 | 1 | 1 |
|  |  | 3 | 1 | | 1 | | .67 | | 1 | | 1 | 1 | 1 | .67 | 1 | 1 |
|  |  | 10 | 1 | | 1 | | .67 | | 1 | | 1 | 1 | 1 | .67 | 1 | 1 |
|  | Probability of Gain | .1 | 1 | | 1 | | .67 | | 1 | | 1 | 1 | 1 | .67 | 1 | 1 |
|  |  | .5 | 1 | | 1 | | .67 | | 1 | | 1 | 1 | 1 | .67 | 1 | 1 |
|  |  | .9 | 1 | | 1 | | .67 | | 1 | | 1 | 1 | 1 | .67 | 1 | 1 |
| *Sim 5F*  Consumption:  None  Anticipation:  High Pres Bias  Low Rate | Gain/Loss Ratio | .25 | 1 | | 1 | | 1 | | 1 | | 1 | 1 | 1 | 1 | 1 | 1 |
|  |  | 1 | 1 | | 1 | | 1 | | 1 | | 1 | 1 | 1 | 1 | 1 | 1 |
|  |  | 4 | 1 | | 1 | | 0 | | 1 | | 1 | 1 | 1 | 0 | 1 | 1 |
|  | Delay | 1 | 1 | | 1 | | .67 | | 1 | | 1 | 1 | 1 | .67 | 1 | 1 |
|  |  | 3 | 1 | | 1 | | .67 | | 1 | | 1 | 1 | 1 | .67 | 1 | 1 |
|  |  | 10 | 1 | | 1 | | .67 | | 1 | | 1 | 1 | 1 | .67 | 1 | 1 |
|  | Probability of Gain | .1 | 1 | | 1 | | .67 | | 1 | | 1 | 1 | 1 | .67 | 1 | 1 |
|  |  | .5 | 1 | | 1 | | .67 | | 1 | | 1 | 1 | 1 | .67 | 1 | 1 |
|  |  | .9 | 1 | | 1 | | .67 | | 1 | | 1 | 1 | 1 | .67 | 1 | 1 |
| *Sim 5G*  Consumption:  None  Anticipation:  Low Pres Bias  High Rate | Gain/Loss Ratio | .25 | 1 | | 1 | | 1 | | 1 | | 1 | 1 | 1 | 1 | 1 | 1 |
|  |  | 1 | 1 | | 1 | | 1 | | 1 | | 1 | 1 | 1 | 1 | 1 | 1 |
|  |  | 4 | 1 | | 1 | | 0 | | 1 | | 1 | 1 | 1 | 0 | 1 | 1 |
|  | Delay | 1 | 1 | | 1 | | .67 | | 1 | | 1 | 1 | 1 | .67 | 1 | 1 |
|  |  | 3 | 1 | | 1 | | .67 | | 1 | | 1 | 1 | 1 | .67 | 1 | 1 |
|  |  | 10 | 1 | | 1 | | .67 | | 1 | | 1 | 1 | 1 | .67 | 1 | 1 |
|  | Probability of Gain | .1 | 1 | | 1 | | .67 | | 1 | | 1 | 1 | 1 | .67 | 1 | 1 |
|  |  | .5 | 1 | | 1 | | .67 | | 1 | | 1 | 1 | 1 | .67 | 1 | 1 |
|  |  | .9 | 1 | | 1 | | .67 | | 1 | | 1 | 1 | 1 | .67 | 1 | 1 |
| *Sim 5H*  Consumption:  None  Anticipation:  High Pres Bias  High Rate | Gain/Loss Ratio | .25 | 1 | | 1 | | 1 | | 1 | | 1 | 1 | 1 | 1 | 1 | 1 |
|  |  | 1 | 1 | | 1 | | 1 | | 1 | | 1 | 1 | 1 | 1 | 1 | 1 |
|  |  | 4 | 1 | | 1 | | 0 | | 1 | | 1 | 1 | 1 | 0 | 1 | 1 |
|  | Delay | 1 | 1 | | 1 | | .67 | | 1 | | 1 | 1 | 1 | .67 | 1 | 1 |
|  |  | 3 | 1 | | 1 | | .67 | | 1 | | 1 | 1 | 1 | .67 | 1 | 1 |
|  |  | 10 | 1 | | 1 | | .67 | | 1 | | 1 | 1 | 1 | .67 | 1 | 1 |
|  | Probability of Gain | .1 | 1 | | 1 | | .67 | | 1 | | 1 | 1 | 1 | .67 | 1 | 1 |
|  |  | .5 | 1 | | 1 | | .67 | | 1 | | 1 | 1 | 1 | .67 | 1 | 1 |
|  |  | .9 | 1 | | 1 | | .67 | | 1 | | 1 | 1 | 1 | .67 | 1 | 1 |

*Note*. The table (Sims 5A–H) reports the results of Simulation 5, using the same summarization method as Tables A4-A6.

**Simulation 6: Varying the Time Profile of Anticipation**

A final issue we consider is how anticipatory utility is distributed across time. Consider the four curves shown in Figure A2.


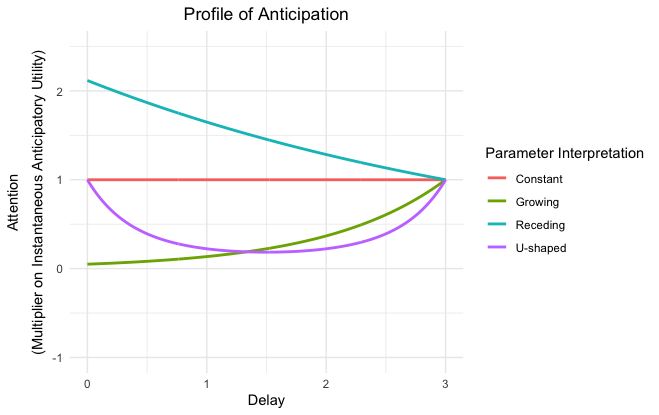


**Figure A2.** Profiles of Anticipation (Attention Functions) Used in Simulation 6.

The horizontal axis represents time from the moment of choice to the moment the outcome is revealed (period 3 in this example), while the horizontal axis is the multiplier on anticipatory utility, the attention function $a\left( t \right)$. The red line (“constant”) depicts a constant multiplier of 1 at all times, meaning that anticipatory utility accumulates at an unchanging rate. The green line (“growing”) depicts an increasing pattern of greater anticipation as the outcome approaches, while the blue line (“receding”) depicts a decreasing pattern of weaker anticipation as the outcome approaches. Finally, the purple line (“U-shaped”) depicts a decreasing and then increasing pattern, which may be the most psychologically realistic pattern of attention: Dread and savoring loom large immediately after making a choice, but fade into the background until the outcome approaches.

To incorporate these trajectories into our formal framework, we implement $a\left( t \right)=e^{-z_{1}*\left( T-t \right)+z_{2}*\left( t-z_{3}T \right)^{2}-z_{2}T^{2}*(1-z_{3})^{2}}$, where $t$ is the current time (position on the horizontal axis of Figure A2), $T$ is the total delay, and $z_{1}$, $z_{2}$, and $z_{3}$ are parameters that govern the shape of the anticipation profile. Although this expression is messy, it has a simple interpretation. The first (linear) term in the exponent controls the overall slope ($z_{1}>0$ for a growing profile, $z_{1}<0$ for a receding profile), the second (quadratic) term controls the shape ($z_{2}>0$ for a U-shape, $z_{2}<0$ for an upside-down U-shape) and distribution of the bend (we set $z_{3}=0.5$, so that the minimum occurs at the halfway point between 0 and $T$), and the last term is a norming term (not dependent on $t$) so that attention is always equal to 1 at time $T$, i.e., $a\left( T \right)=1$.

**Table A8.** *Summary Results of Simulations 1 and 6 (Varying Profile of Anticipation)*

| Value Functions | Choice Characteristic | | | Proportion of Negative Regression Coefficients | | | | | | | | | | | | |
| --- | --- | --- | --- | --- | --- | --- | --- | --- | --- | --- | --- | --- | --- | --- | --- | --- |
|  |  |  |  | Risk Preferences | | | | | | | | Time Preferences | | | | |
|  |  |  |  | $\sigma$ | | $\lambda$ | | $\alpha$ | | $\sigma*\alpha$ | $\lambda*\alpha$ | $\sigma$ | $\lambda$ | $\alpha$ | $\sigma*\alpha$ | $\lambda*\alpha$ |
| *Sim 1*  Constant Profile | Gain/Loss Ratio | .25 | 1 | | 1 | | 1 | | 1 | | 1 | 1 | 1 | 1 | 1 | 1 |
|  |  | 1 | 1 | | 1 | | 1 | | 1 | | 1 | 1 | 1 | 1 | 1 | 1 |
|  |  | 4 | 1 | | 1 | | 0 | | 1 | | 1 | 1 | 1 | 0 | 1 | 1 |
|  | Delay | 1 | 1 | | 1 | | .67 | | 1 | | 1 | 1 | 1 | .67 | 1 | 1 |
|  |  | 3 | 1 | | 1 | | .67 | | 1 | | 1 | 1 | 1 | .67 | 1 | 1 |
|  |  | 10 | 1 | | 1 | | .67 | | 1 | | 1 | 1 | 1 | .67 | 1 | 1 |
|  | Probability of Gain | .1 | 1 | | 1 | | .67 | | 1 | | 1 | 1 | 1 | .67 | 1 | 1 |
|  |  | .5 | 1 | | 1 | | .67 | | 1 | | 1 | 1 | 1 | .67 | 1 | 1 |
|  |  | .9 | 1 | | 1 | | .67 | | 1 | | 1 | 1 | 1 | .67 | 1 | 1 |
| *Sim 6A*  Growing Profile | Gain/Loss Ratio | .25 | 1 | | 1 | | 1 | | 1 | | 1 | 1 | 1 | 1 | 1 | 1 |
|  |  | 1 | 1 | | 1 | | 1 | | 1 | | 1 | 1 | 1 | 1 | 1 | 1 |
|  |  | 4 | 1 | | 1 | | 0 | | 1 | | 1 | 1 | 1 | 0 | 1 | 1 |
|  | Delay | 1 | 1 | | 1 | | .67 | | 1 | | 1 | 1 | 1 | .67 | 1 | 1 |
|  |  | 3 | 1 | | 1 | | .67 | | 1 | | 1 | 1 | 1 | .67 | 1 | 1 |
|  |  | 10 | 1 | | 1 | | .67 | | 1 | | 1 | 1 | 1 | .67 | 1 | 1 |
|  | Probability of Gain | .1 | 1 | | 1 | | .67 | | 1 | | 1 | 1 | 1 | .67 | 1 | 1 |
|  |  | .5 | 1 | | 1 | | .67 | | 1 | | 1 | 1 | 1 | .67 | 1 | 1 |
|  |  | .9 | 1 | | 1 | | .67 | | 1 | | 1 | 1 | 1 | .67 | 1 | 1 |
| *Sim 6B*  Receding Profile | Gain/Loss Ratio | .25 | 1 | | 1 | | 1 | | 1 | | 1 | 1 | 1 | 1 | 1 | 1 |
|  |  | 1 | 1 | | 1 | | 1 | | 1 | | 1 | 1 | 1 | 1 | 1 | 1 |
|  |  | 4 | 1 | | 1 | | 0 | | 1 | | 1 | 1 | 1 | 0 | 1 | 1 |
|  | Delay | 1 | 1 | | 1 | | .67 | | 1 | | 1 | 1 | 1 | .67 | 1 | 1 |
|  |  | 3 | 1 | | 1 | | .67 | | 1 | | 1 | 1 | 1 | .67 | 1 | 1 |
|  |  | 10 | 1 | | 1 | | .67 | | 1 | | 1 | 1 | 1 | .67 | 1 | 1 |
|  | Probability of Gain | .1 | 1 | | 1 | | .67 | | 1 | | 1 | 1 | 1 | .67 | 1 | 1 |
|  |  | .5 | 1 | | 1 | | .67 | | 1 | | 1 | 1 | 1 | .67 | 1 | 1 |
|  |  | .9 | 1 | | 1 | | .67 | | 1 | | 1 | 1 | 1 | .67 | 1 | 1 |
| *Sim 6C*  U-Shaped Profile | Gain/Loss Ratio | .25 | 1 | | 1 | | 1 | | 1 | | 1 | 1 | 1 | 1 | 1 | 1 |
|  |  | 1 | 1 | | 1 | | 1 | | 1 | | 1 | 1 | 1 | 1 | 1 | 1 |
|  |  | 4 | 1 | | 1 | | 0 | | 1 | | 1 | 1 | 1 | 0 | 1 | 1 |
|  | Delay | 1 | 1 | | 1 | | .67 | | 1 | | 1 | 1 | 1 | .67 | 1 | 1 |
|  |  | 3 | 1 | | 1 | | .67 | | 1 | | 1 | 1 | 1 | .67 | 1 | 1 |
|  |  | 10 | 1 | | 1 | | .67 | | 1 | | 1 | 1 | 1 | .67 | 1 | 1 |
|  | Probability of Gain | .1 | 1 | | 1 | | .67 | | 1 | | 1 | 1 | 1 | .67 | 1 | 1 |
|  |  | .5 | 1 | | 1 | | .67 | | 1 | | 1 | 1 | 1 | .67 | 1 | 1 |
|  |  | .9 | 1 | | 1 | | .67 | | 1 | | 1 | 1 | 1 | .67 | 1 | 1 |

*Note*. The top fourth of the table (Sim 1) is identical to the top panel of Tables A4-A6. The rest of the table (Sims 6A–C) reports the results of Simulation 6, using the same summarization method as Tables A4–A7.

For these simulations, we return to linear utility, linear probability weighting, and no discounting. The constant profile we use (Simulation 1 as a benchmark comparison) is equivalent to setting $z_{1}=0$ and $z_{2}=0$. Simulations 6A, 6B, and 6C, respectively test a growing profile ($z_{1}=1$ and $z_{2}=0$), receding profile ($z_{1}=-0.25$ and $z_{2}=0$) and U-shaped profile ($z_{1}=0$ and $z_{2}=0.75$). These are also the parameters used to generate Figure A2.

As shown in Table A8, the results are very similar to the other simulations—indeed, identical in all respects to Simulation 1. Although varying the profile of anticipation likely has important effects on decision-making, it does not interact with the effects we examine in this paper.

**Conclusions and Future Directions**

The overall conclusion we draw from these analyses is that the central implications of the theoretical model presented in the main text—the links between dread aversion and risk-avoidance, loss aversion and risk-avoidance, and dread aversion and impatience—are inescapable results of the theoretical framework. We showed in the main text that these results follow from extremely general assumptions—e.g., value functions that are increasing—and the simulation results confirm what we showed analytically.

However, the simulation results also went beyond the proofs in the main text in a few respects. First, they examined the boundary conditions on the other theorized effect—the link between loss aversion and impatience—which does not follow unambiguously from the theoretical model, but which instead depends on the relative balance of two channels—loss aversion incentivizing one to push potential losses into the future to reduce consumption disutility and to push potential losses sooner to reduce anticipatory disutility. We explored some of the choice characteristics and functional forms that can cause this effect to vary in direction. Second, they examined the role of vividness in producing risk-avoidance and impatience—which we did not consider analytically in the main text—and uncovered several factors that impact the direction of this effect. Third, we tested the interaction effects between vividness and the four primary effects of interest, finding that vividness consistently exacerbated the effects.

We plan to extend this modelling framework in a variety of directions, many of which are described in the Discussion of the main text. We see the simulation method developed here as an important complementary approach for cases in which the expressions become mathematically intractable. For example, a further complication we did not consider was whether the parameterizations of the value, weighting, discounting, and attention functions might vary between gains and losses—a possibility for which there is some evidence (e.g., Abdellaoui, 2000).

Another possibility we did not explore is varying different types of functions within the same simulation—for instance, testing a prospect theory value function, a step function for weighting anticipatory emotions, hyperbolic discounting, and a U-shaped pattern of attention all in the same model. We remain confident that the core predictions of the theory will remain unqualified by such interactions, as we do not envision that any such cases would lead the broad assumptions made in our analysis to fail. Still, the other effects we tested may depend in interesting ways on such interactions, as might some of the speculations in the Discussion about potential extensions to and predictions made by the model.

Finally, we note that a limitation of the simulation method used here is that we restricted ourselves to studying the qualitative behavior of the model (the sign of the regression coefficients) rather than comparing magnitudes. We did this partly for tractability and partly for comparability, but we suspect our approach could be extended to make more precise quantitative predictions. Doing so may yield further hypotheses for future work.

**References**

Abdellaoui, M. (2000). Parameter-free elicitation of utility and probability weighting functions. *Management Science*, *46*, 1497–1512.

Hardisty, D. J., & Weber, E. U. (2020). Impatience and savoring vs. dread: Asymmetries in anticipation explain consumer time preferences for positive vs. negative events. *Journal of Consumer Psychology*, *30*, 598-613.

Lattimore, P. K., Baker, J. R., & Witte, A. D. (1992). The influence of probability on risky choice: A parametric examination. *Journal of Economic Behavior & Organization*, *17*, 377–400.

Loewenstein, G., & Prelec, D. (1992). Anomalies in intertemporal choice: Evidence and an interpretation. *Quarterly Journal of Economics*, *107*, 573–597.

Loewenstein, G. F., Weber, E. U., Hsee, C. K., & Welch, N. (2001). Risk as feelings. *Psychological Bulletin, 127*, 267–286.

Thaler, R. (1981). Some empirical evidence on dynamic inconsistency. *Economics Letters*, *8*, 201–207.

Tversky, A., & Kahneman, D. (1992). Advances in prospect theory: Cumulative representation of uncertainty. *Journal of Risk and Uncertainty*, *5*, 297–323.

Yao, S., Mela, C. F., Chiang, J., & Chen, Y. (2012). Determining consumers’ discount rates with field studies. *Journal of Marketing Research*, *49*, 822–841.

**Appendix B**

**Table B1.** *Descriptive Statistics for the Sample used to Estimate Asymmetric Anticipatory Emotions and Asymmetric Reactive Emotions*.

|  | Mean/ Frequency | Std. Dev. | Min | Max |
| --- | --- | --- | --- | --- |
| *Dependent variable:* |  |  |  |  |
| General Health Questionnaire – 36 Point Scale | 24.979 | 5.278 | 0 | 36 |
| *Financial expectations and realizations:* |  |  |  |  |
| Expectation: Worse off | 0.118 |  | 0 | 1 |
| Expectation: No change | 0.593 |  | 0 | 1 |
| Expectation: Better off | 0.289 |  | 0 | 1 |
| Realization: Worse off | 0.240 |  | 0 | 1 |
| Realization: No change | 0.473 |  | 0 | 1 |
| Realization: Better off | 0.287 |  | 0 | 1 |
| *Controls:* |  |  |  |  |
| Age | 44.691 | 17.988 | 15 | 98 |
| *Highest academic qualification:* |  |  |  |  |
| University/college degree | 0.119 |  | 0 | 1 |
| Other higher degree | 0.084 |  | 0 | 1 |
| A-level’s | 0.202 |  | 0 | 1 |
| GCSE’s | 0.260 |  | 0 | 1 |
| Other qualification | 0.114 |  | 0 | 1 |
| No qualification | 0.114 |  | 0 | 1 |
| *Marital status:* |  |  |  |  |
| Married | 0.567 |  | 0 | 1 |
| Living as a couple | 0.113 |  | 0 | 1 |
| Widowed, divorced or separated | 0.132 |  | 0 | 1 |
| Single, never married | 0.189 |  | 0 | 1 |
| *Housing tenure:* |  |  |  |  |
| Own house outright | 0.255 |  | 0 | 1 |
| Own house with mortgage | 0.508 |  | 0 | 1 |
| Private sector renter | 0.079 |  | 0 | 1 |
| Local authority renter | 0.158 |  | 0 | 1 |
| *Economic activity:* |  |  |  |  |
| Employee | 0.536 |  | 0 | 1 |
| Self-employed | 0.072 |  | 0 | 1 |
| Unemployed | 0.033 |  | 0 | 1 |
| Full-time education | 0.053 |  | 0 | 1 |
| Retired | 0.187 |  | 0 | 1 |
| Economically inactive | 0.120 |  | 0 | 1 |
| *Household income and composition:* |  |  |  |  |
| Log of household income (deflated) | 7.872 | 0.787 | -2.120 | 11.517 |
| Number of dependent children in household | 0.597 | 0.956 | 0 | 9 |
| Square root of household size | 1.644 | 0.390 | 1 | 3.742 |
| Observations | 125,026 |  |  |  |
| Individuals | 13,969 |  |  |  |

*Note.* Highest academic qualification dummy variables indicate the highest level of attainment. These educational dummy variables are: university/college degree - either at undergraduate or postgraduate level; other higher degree - which includes work-related, or vocational, higher education qualifications such as Higher National Diplomas (HND) and Higher National Certificates (HNC); A-level’s – which are post-compulsory examinations taken at 18 to qualify for college or university entrance; GCSE’s – which are schooling attainment qualifications taken at 16; Other qualifications – which includes school leaving exam certificate or matriculation; and lastly, no formal qualifications.

**Table B2.** *Estimating Asymmetric Anticipatory Emotions and Asymmetric Reactive Emotions.*

| Dependent Variable: | General Health Questionnaire – 36 Point Scale | |
| --- | --- | --- |
|  | Regression 1 | Regression 2 |
| *Financial expectations and realizations:* |  |  |
| Expectation: Worse off | -0.582*** |  |
|  | [-12.822] |  |
| Expectation: Better off | 0.094*** |  |
|  | [2.701] |  |
| Realization: Worse off |  | -1.063*** |
|  |  | [-27.610] |
| Realization: Better off |  | 0.519*** |
|  |  | [16.372] |
| *Control variables:* |  |  |
| Age (years) | -0.114** | -0.087 |
|  | [-1.998] | [-1.542] |
| Age squared (years) | 0.000* | 0.000 |
|  | [1.847] | [0.712] |
| University/college degree | 0.415 | 0.568** |
|  | [1.438] | [1.992] |
| Other higher degree | 0.503 | 0.673** |
|  | [1.625] | [2.202] |
| A-level | 0.305 | 0.440** |
|  | [1.429] | [2.093] |
| GCSE's | 0.049 | 0.121 |
|  | [0.259] | [0.647] |
| Other qualification | 0.257 | 0.279 |
|  | [1.191] | [1.312] |
| Married | -0.311*** | -0.219* |
|  | [-2.600] | [-1.854] |
| Living as a couple | -0.017 | 0.063 |
|  | [-0.179] | [0.665] |
| Widowed, divorced or separated | -1.174*** | -0.957*** |
|  | [-8.456] | [-6.964] |
| Own house outright | -0.125 | -0.127 |
|  | [-1.076] | [-1.110] |
| Own house with mortgage | -0.205** | -0.161 |
|  | [-1.994] | [-1.584] |
| Private sector renter | -0.116 | -0.074 |
|  | [-1.099] | [-0.706] |
| Employee | 0.772*** | 0.534*** |
|  | [12.355] | [8.607] |
| Self-employed | 0.917*** | 0.725*** |
|  | [9.478] | [7.569] |
| Unemployed | -0.760*** | -0.435*** |
|  | [-8.296] | [-4.761] |
| Full-time education | 0.951*** | 0.936*** |
|  | [8.823] | [8.795] |
| Retired | 0.942*** | 0.922*** |
|  | [11.653] | [11.519] |
| Log of household income (deflated) | 0.158*** | 0.049* |
|  | [5.412] | [1.694] |
| Number of dependent children in household | 0.030 | 0.053 |
|  | [0.684] | [1.240] |
| Square root of household size | -0.371*** | -0.345*** |
|  | [-3.753] | [-3.526] |
| Region controls | Yes | Yes |
| Year (survey wave) controls | Yes | Yes |
| Random effects parameters: |  |  |
| $\sigma_{z0}$ | 2.920*** | 2.724*** |
| $\sigma_{z1}$ | 1.022*** |  |
| $\sigma_{z2}$ | 1.174*** | 1.815*** |
| $\rho_{z0,z1}$ | -0.183*** |  |
| $\rho_{z0,z2}$ | 0.233*** | 0.238*** |
| $\rho_{z1,z2}$ | -0.428*** |  |
|  |  |  |
| Observations | 125,026 | 125,026 |
| Individuals | 13,969 | 13,969 |

*Note.* Entries are results of Multilevel regressions estimating asymmetric anticipatory emotions and asymmetric reactive emotions. Robust t-statistics in brackets. The multilevel regressions are estimated with an unstructured covariance matrix to allow for correlation between the random effect attached to the intercept and the random effects attached to the within-person coefficients. We assume the within-person errors follow an autoregressive structure of order 2. This assumption is confirmed by model selection criteria (in particular, the Akaike and Bayesian Information Criteria). The regressions include the individual time means of all the time-varying independent variables as well as forwarded control variables (i.e., $t+1$) and their respective individual time means. The random-effects parameters $\sigma_{z0}$, $\sigma_{z1}$and $\sigma_{z2}$ are the estimates of the random coefficient standard deviation for the intercept and the within-person coefficients “Better off” and “Worse off,” respectively;$\rho_{z0,z1}$ and $\rho_{z0,z2}$ are the correlations between the intercept and the within-person coefficients and $\rho_{z1,z2}$ is the correlation between the within-person coefficients.

$$*p< .1. **p< .05. ***p< .01.$$

**Table B3.** *Predictors of Risk Preferences.*

| Dependent variable: Willingness to take risks in general | | | | |
| --- | --- | --- | --- | --- |
| Predictors | Regression 1 | Regression 2 | Regression 3 | Regression4 |
| *Anticipatory and realized emotions:* |  |  |  |  |
| Asymmetric anticipatory emotions | -0.130*** | -0.089*** | -0.073*** | -0.079*** |
|  | [-5.630] | [-3.603] | [-2.977] | [-3.245] |
| Asymmetric reactive emotions |  | -0.111*** | -0.102*** | -0.093*** |
|  |  | [-4.197] | [-3.912] | [-3.555] |
| *Control variables:* |  |  |  |  |
| Age (years) | -0.042*** | -0.040*** | -0.025*** | -0.030*** |
|  | [-4.714] | [-4.475] | [-2.738] | [-2.750] |
| Age squared (years) | 0.000 | 0.000 | 0.000 | 0.000** |
|  | [1.390] | [1.106] | [0.872] | [2.025] |
| Female | -0.682*** | -0.676*** | -0.547*** | -0.474*** |
|  | [-12.907] | [-12.811] | [-10.241] | [-8.699] |
| Trait pessimism |  |  | -0.406** | -0.522*** |
|  |  |  | [-2.045] | [-2.617] |
| Trait optimism |  |  | 1.317*** | 1.114*** |
|  |  |  | [11.562] | [9.745] |
| State GHQ |  |  | 0.034*** | 0.029*** |
|  |  |  | [5.501] | [4.816] |
| Anxiety or depression |  |  | -0.385*** | -0.301*** |
|  |  |  | [-6.168] | [-4.741] |
| University/college degree |  |  |  | 0.610*** |
|  |  |  |  | [5.666] |
| Other higher degree |  |  |  | 0.630*** |
|  |  |  |  | [5.356] |
| A-level |  |  |  | 0.366*** |
|  |  |  |  | [3.697] |
| GCSE's' |  |  |  | 0.408*** |
|  |  |  |  | [4.312] |
| Other qualification |  |  |  | 0.156 |
|  |  |  |  | [1.416] |
| Married |  |  |  | -0.044 |
|  |  |  |  | [-0.450] |
| Living as a couple |  |  |  | 0.134 |
|  |  |  |  | [1.286] |
| Widowed, divorced or separated |  |  |  | 0.081 |
|  |  |  |  | [0.701] |
| Own house outright |  |  |  | -0.102 |
|  |  |  |  | [-1.072] |
| Own house with mortgage |  |  |  | -0.105 |
|  |  |  |  | [-1.144] |
| Private sector renter |  |  |  | 0.140 |
|  |  |  |  | [1.147] |
| Employee |  |  |  | 0.336*** |
|  |  |  |  | [3.550] |
| Self-employed |  |  |  | 0.834*** |
|  |  |  |  | [6.672] |
| Unemployed |  |  |  | 0.179 |
|  |  |  |  | [0.927] |
| Full-time education |  |  |  | 1.042*** |
|  |  |  |  | [5.024] |
| Retired |  |  |  | 0.134 |
|  |  |  |  | [1.049] |
| Log of household income (deflated) |  |  |  | 0.151*** |
|  |  |  |  | [3.140] |
| Number of children in household |  |  |  | 0.002 |
|  |  |  |  | [0.050] |
| Square root of household size |  |  |  | -0.021 |
|  |  |  |  | [-0.179] |
|  |  |  |  |  |
| Exogeneous controls | Yes | Yes | Yes | Yes |
| Trait optimism/anxiety/state GHQ | No | No | Yes | Yes |
| Sociodemographic controls | No | No | No | Yes |
| R-squared | 0.085 | 0.088 | 0.120 | 0.144 |
| Observations | 6839 | 6839 | 6839 | 6839 |
| Individuals | 6839 | 6839 | 6839 | 6839 |

*Note.* Entries are results from OLS regressions estimating the determinants of the general risk question—ranging from 1 (“*unwilling to take risks*”) to 10 (“*fully prepared to take risks*”). Unadjusted coefficients are reported, with t-statistics based on robust standard errors shown in brackets. Asymmetric anticipatory and reactive emotions are standardized and measures higher ‘disutility’ from anticipating or experiencing income losses relative to ‘utility’ from income gains. Exogenous controls include age (entered in both linear and quadratic form) and sex. Trait optimism/pessimism is captured by two variables, measured as the individual's time-averaged (Waves 1-17, BHPS) financial expectations of “better off” and “worse off”. Anxiety is a binary variable equal to one if the respondent ever reported anxiety or depression during Waves 1-17 of the BHPS, and zero otherwise. State GHQ (General Health Questionnaire) is calculated as the individual's current GHQ score minus their time-averaged (Waves 1-17, BHPS) GHQ score. Sociodemographic controls include current education level (highest academic qualification), current marital status, current housing tenure, current labor market status, logarithm of monthly household income (deflated) for the current year, current number of children under the age of 16 present in the household and square root of current household size. We use analytical weights to account for variation in the number of observations per individual when estimating asymmetric anticipatory and reactive emotions. Individuals observed more frequently are given greater weight, reflecting the higher precision of their emotion estimates.

$$*p< .1 **p< .05 ***p< .01$$

**Table B4.** *Predictors of Risk Preferences – Ordered Logistic Regression*

| Dependent variable: Willingness to take risks in general | | | | |
| --- | --- | --- | --- | --- |
| Predictors | Regression 1 | Regression 2 | Regression 3 | Regression4 |
| Asymmetric anticipatory emotions | 0.888*** | 0.917*** | 0.927*** | 0.922*** |
|  | [-5.849] | [-3.964] | [-3.469] | [-3.615] |
| Asymmetric reactive emotions |  | 0.912*** | 0.917*** | 0.922*** |
|  |  | [-3.895] | [-3.721] | [-3.385] |
|  |  |  |  |  |
| Exogeneous controls | Yes | Yes | Yes | Yes |
| Trait optimism/anxiety/state GHQ | No | No | Yes | Yes |
| Sociodemographic controls | No | No | No | Yes |
| Observations | 6839 | 6839 | 6839 | 6839 |
| Individuals | 6839 | 6839 | 6839 | 6839 |

*Note.* Entries are results from Ordered Logistic regressions estimating the determinants of the general risk question——ranging from 1 (“*unwilling to take risks*”) to 10 (“*fully prepared to take risks*”). Odds ratios are reported, with t-statistics based on robust standard errors shown in brackets. Asymmetric anticipatory and reactive emotions are standardized and measures higher ‘disutility’ from anticipating or experiencing income losses relative to ‘utility’ from income gains. We use analytical weights to account for variation in the number of observations per individual when estimating asymmetric anticipatory and reactive emotions. Individuals observed more frequently are given greater weight, reflecting the higher precision of their emotion estimates. Lastly, the parallel lines (proportional odds) assumption—where the effect of each predictor variable on the outcome is consistent across all categories—is met for our measures of asymmetric anticipatory and reactive emotions within the Ordered Logistic regressions. Moreover, the unreported results, from a Partial Proportional Odds Model—which relaxes the parallel lines assumption of the Ordered Logistic model—are quantitively and qualitatively similar. For details about the control variables see the notes for Table B3.

$$*p< .1 **p< .05 ***p< .01$$

**Table B5.** *Predictors of Risk Preferences – Interval Regression*

| Dependent variable: Willingness to take risks in general | | | | |
| --- | --- | --- | --- | --- |
| Predictors | Regression 1 | Regression 2 | Regression 3 | Regression4 |
| Asymmetric anticipatory emotions | -0.132*** | -0.090*** | -0.074*** | -0.080*** |
|  | [-5.627] | [-3.600] | [-2.987] | [-3.274] |
| Asymmetric reactive emotions |  | -0.113*** | -0.103*** | -0.094*** |
|  |  | [-4.208] | [-3.912] | [-3.572] |
|  |  |  |  |  |
| Exogeneous controls | Yes | Yes | Yes | Yes |
| Trait optimism/anxiety/state GHQ | No | No | Yes | Yes |
| Sociodemographic controls | No | No | No | Yes |
| Observations | 6839 | 6839 | 6839 | 6839 |
| Individuals | 6839 | 6839 | 6839 | 6839 |

*Note.* Entries are results from interval regressions estimating the determinants of the general risk question—ranging from 1 (“*unwilling to take risks*”) to 10 (“*fully prepared to take risks*”). Unadjusted coefficients are reported, with t-statistics based on robust standard errors shown in brackets. As responses to the general risk question are bounded and ordinal, someone who reports a “7” is telling us their true risk tolerance lies somewhere above 6 and below 8—not an exact value, but an interval. Treating this as a continuous variable in OLS ignores that imprecision and the scale's limits. Interval regression is more appropriate because it models the underlying, unobserved risk preference as a continuous latent variable and accounts for the fact that each observed value represents a range. Asymmetric anticipatory and reactive emotions are standardized and measures higher ‘disutility’ from anticipating or experiencing income losses relative to ‘utility’ from income gains. We use analytical weights to account for variation in the number of observations per individual when estimating asymmetric anticipatory and reactive emotions. Individuals observed more frequently are given greater weight, reflecting the higher precision of their emotion estimates. For details about the control variables see the notes for Table B3.

$$*p< .1 **p< .05 ***p< .01$$

**Table B6.** *Predictors of Risk Preferences – Logistic Regression*

| Dependent variable: High vs. low willingness to take risks in general | | | | |
| --- | --- | --- | --- | --- |
| Predictors | Regression 1 | Regression 2 | Regression 3 | Regression4 |
| Asymmetric anticipatory emotions | 0.883*** | 0.909*** | 0.912*** | 0.905*** |
|  | [-5.074] | [-3.622] | [-3.422] | [-3.684] |
| Asymmetric reactive emotions |  | 0.926*** | 0.931** | 0.941** |
|  |  | [-2.766] | [-2.536] | [-2.112] |
|  |  |  |  |  |
| Exogeneous controls | Yes | Yes | Yes | Yes |
| Trait optimism/anxiety/state GHQ | No | No | Yes | Yes |
| Sociodemographic controls | No | No | No | Yes |
| Observations | 6839 | 6839 | 6839 | 6839 |
| Individuals | 6839 | 6839 | 6839 | 6839 |

*Note.* Entries are results from Logistic regressions estimating the determinants of the general risk question—ranging from 1 (“*unwilling to take risks*”) to 10 (“*fully prepared to take risks”)—*which has been dichotomized into high vs. low risk (equal to one if the respondent is in the highest quartile of the risk distribution, and zero otherwise). Odds ratios are reported, with t-statistics based on robust standard errors shown in brackets. Asymmetric anticipatory and reactive emotions are standardized and measures higher ‘disutility’ from anticipating or experiencing income losses relative to ‘utility’ from income gains. For details about the control variables see the notes for Table B3.

$$*p< .1 **p< .05 ***p< .01$$

**Table B7.** *Predictors of Risk Preferences – Winsorizing Asymmetric Emotions*

| Dependent variable: Willingness to take risks in general | | | | |
| --- | --- | --- | --- | --- |
| Predictors | Regression 1 | Regression 2 | Regression 3 | Regression4 |
| Asymmetric anticipatory emotions | -0.163*** | -0.108*** | -0.088*** | -0.096*** |
|  | [-5.730] | [-3.488] | [-2.874] | [-3.174] |
| Asymmetric reactive emotions |  | -0.138*** | -0.125*** | -0.113*** |
|  |  | [-4.493] | [-4.092] | [-3.684] |
|  |  |  |  |  |
| Exogeneous controls | Yes | Yes | Yes | Yes |
| Trait optimism/anxiety/state GHQ | No | No | Yes | Yes |
| Sociodemographic controls | No | No | No | Yes |
| R-squared | 0.085 | 0.089 | 0.121 | 0.146 |
| Observations | 6839 | 6839 | 6839 | 6839 |
| Individuals | 6839 | 6839 | 6839 | 6839 |

*Note.* Entries are results from OLS regressions estimating the determinants of the general risk question—ranging from 1 (“*unwilling to take risks*”) to 10 (“*fully prepared to take risks*”). Unadjusted coefficients are reported, with t-statistics based on robust standard errors shown in brackets. Asymmetric anticipatory and reactive emotions are standardized and measures higher ‘disutility’ from anticipating or experiencing income losses relative to ‘utility’ from income gains. We winsorize the asymmetric anticipatory and reactive emotions at the 5^th^ and 95^th^ percentiles to mitigate the potential impact of outliers. We use analytical weights to account for variation in the number of observations per individual when estimating asymmetric anticipatory and reactive emotions. Individuals observed more frequently are given greater weight, reflecting the higher precision of their emotion estimates. For details about the control variables see the notes for Table B3.

$$*p< .1 **p< .05 ***p< .01$$

**Table B8.** *Predictors of Risk Preferences – The Impact of Dread and Savoring*

| Dependent variable: Willingness to take risks in general | | | | |
| --- | --- | --- | --- | --- |
| Predictors | Regression 1 | Regression 2 | Regression 3 | Regression4 |
| Dread | -0.217*** | -0.156*** | -0.125*** | -0.138*** |
|  | [-5.937] | [-3.942] | [-3.213] | [-3.567] |
| Savoring | 0.115*** | 0.081** | 0.071** | 0.074** |
|  | [3.402] | [2.287] | [2.044] | [2.132] |
| Asymmetric reactive emotions |  | -0.101*** | -0.096*** | -0.085*** |
|  |  | [-3.794] | [-3.665] | [-3.250] |
| $\left\vert bDread \right\vert-\vert bSavoring\vert$ | 0.102*** | 0.075*** | 0.054** | 0.064** |
|  | [3.950] | [2.850] | [2.100] | [2.540] |
|  |  |  |  |  |
| Exogeneous controls | Yes | Yes | Yes | Yes |
| Trait optimism/anxiety/state GHQ | No | No | Yes | Yes |
| Sociodemographic controls | No | No | No | Yes |
| R-squared | 0.086 | 0.089 | 0.121 | 0.144 |
| Observations | 6839 | 6839 | 6839 | 6839 |
| Individuals | 6839 | 6839 | 6839 | 6839 |

*Note.* Entries are results from OLS regressions estimating the determinants of the general risk question—ranging from 1 (“*unwilling to take risks*”) to 10 (“*fully prepared to take risks*”). Unadjusted coefficients are reported, with t-statistics based on robust standard errors shown in brackets. Dread and savoring are both standardized and measure the ‘disutility’ from anticipating income losses and the ‘utility’ from anticipating income gains, respectively. Asymmetric reactive emotions are standardized and measures higher ‘disutility’ from experiencing income losses relative to ‘utility’ from experiencing income gains. We use analytical weights to account for variation in the number of observations per individual when estimating asymmetric anticipatory and reactive emotions. Individuals observed more frequently are given greater weight, reflecting the higher precision of their emotion estimates. For details about the control variables see the notes for Table B3.

$$*p< .1 **p< .05 ***p< .01$$

**Table B9.** *Predictors of Time Preferences*.

| Dependent variable: Delayed gratification scale | | | | |
| --- | --- | --- | --- | --- |
| Predictors | Regression 1 | Regression 2 | Regression 3 | Regression4 |
| *Anticipatory and realized emotions:* |  |  |  |  |
| Asymmetric anticipatory emotions | -1.030*** | -0.785*** | -0.738*** | -0.827*** |
|  | [-6.447] | [-4.561] | [-4.306] | [-4.964] |
| Asymmetric reactive emotions |  | -0.691*** | -0.620*** | -0.422** |
|  |  | [-3.647] | [-3.210] | [-2.238] |
| *Control variables:* |  |  |  |  |
| Age (years) | 0.172** | 0.186** | 0.196*** | -0.003 |
|  | [2.368] | [2.556] | [2.658] | [-0.039] |
| Age squared (years) | -0.001* | -0.001** | -0.001** | 0.001 |
|  | [-1.817] | [-2.034] | [-2.015] | [0.756] |
| Female | 1.381*** | 1.416*** | 1.755*** | 2.309*** |
|  | [3.607] | [3.702] | [4.522] | [5.865] |
| Trait pessimism |  |  | 0.563 | -1.688 |
|  |  |  | [0.391] | [-1.175] |
| Trait optimism |  |  | 1.824** | 0.830 |
|  |  |  | [2.119] | [0.970] |
| State GHQ |  |  | 0.247*** | 0.220*** |
|  |  |  | [6.106] | [5.521] |
| Anxiety or depression |  |  | -1.956*** | -1.230*** |
|  |  |  | [-4.333] | [-2.712] |
| University/college degree |  |  |  | 7.005*** |
|  |  |  |  | [8.885] |
| Other higher degree |  |  |  | 4.180*** |
|  |  |  |  | [4.869] |
| A-level |  |  |  | 4.748*** |
|  |  |  |  | [6.304] |
| GCSE's' |  |  |  | 3.700*** |
|  |  |  |  | [5.087] |
| Other qualification |  |  |  | 2.528*** |
|  |  |  |  | [3.041] |
| Married |  |  |  | 2.688*** |
|  |  |  |  | [3.581] |
| Living as a couple |  |  |  | 1.680* |
|  |  |  |  | [1.945] |
| Widowed, divorced or separated |  |  |  | 2.254*** |
|  |  |  |  | [2.697] |
| Own house outright |  |  |  | 2.133*** |
|  |  |  |  | [3.024] |
| Own house with mortgage |  |  |  | 0.832 |
|  |  |  |  | [1.177] |
| Private sector renter |  |  |  | -0.248 |
|  |  |  |  | [-0.264] |
| Employee |  |  |  | 1.723** |
|  |  |  |  | [2.094] |
| Self-employed |  |  |  | 4.020*** |
|  |  |  |  | [4.039] |
| Unemployed |  |  |  | 0.726 |
|  |  |  |  | [0.466] |
| Full-time education |  |  |  | 6.130** |
|  |  |  |  | [2.528] |
| Retired |  |  |  | 1.786* |
|  |  |  |  | [1.823] |
| Log of household income (deflated) |  |  |  | 0.783** |
|  |  |  |  | [2.208] |
| Number of children in household |  |  |  | 0.797** |
|  |  |  |  | [2.471] |
| Square root of household size |  |  |  | -2.214** |
|  |  |  |  | [-2.412] |
|  |  |  |  |  |
| Exogeneous controls | Yes | Yes | Yes | Yes |
| Trait optimism/anxiety/state GHQ | No | No | Yes | Yes |
| Sociodemographic controls | No | No | No | Yes |
| R-squared | 0.018 | 0.022 | 0.040 | 0.092 |
| Observations | 4173 | 4173 | 4173 | 4173 |
| Individuals | 4173 | 4173 | 4173 | 4173 |

*Note.* Entries are results from OLS regressions estimating the determinants of the delayed gratification scale—ranging from 0 (impatient) to 100 (patient). Unadjusted coefficients are reported, with t-statistics based on robust standard errors shown in brackets. Asymmetric anticipatory and reactive emotions are standardized and measures higher ‘disutility’ from anticipating or experiencing income losses relative to ‘utility’ from income gains. Exogenous controls include age (entered in both linear and quadratic form) and sex. Trait optimism/pessimism is captured by two variables, measured as the individual's time-averaged (Waves 1-17, BHPS) financial expectations of “better off” and “worse off”. Anxiety is a binary variable equal to one if the respondent ever reported anxiety or depression during Waves 1-17 of the BHPS, and zero otherwise. State GHQ (General Health Questionnaire) is calculated as the individual's current GHQ score minus their time-averaged (Waves 1-17, BHPS) GHQ score. Sociodemographic controls include current education level (highest academic qualification), current marital status, current housing tenure, current labor market status, logarithm of monthly household income (deflated) for the current year, current number of children under the age of 16 present in the household and square root of current household size. We use analytical weights to account for variation in the number of observations per individual when estimating asymmetric anticipatory and reactive emotions. Individuals observed more frequently are given greater weight, reflecting the higher precision of their emotion estimates.

$$*p< .1 **p< .05 ***p< .01$$

**Table B10.** *Predictors of Time Preferences – Ordered Logistic Regression*

| Dependent variable: Quartiles of the delayed gratification scale | | | | |
| --- | --- | --- | --- | --- |
| Predictors | Regression 1 | Regression 2 | Regression 3 | Regression4 |
| Asymmetric anticipatory emotions | 0.866*** | 0.897*** | 0.899*** | 0.884*** |
|  | [-5.722] | [-3.869] | [-3.781] | [-4.352] |
| Asymmetric reactive emotions |  | 0.905*** | 0.910*** | 0.936** |
|  |  | [-3.353] | [-3.068] | [-2.153] |
|  |  |  |  |  |
| Exogeneous controls | Yes | Yes | Yes | Yes |
| Trait optimism/anxiety/state GHQ | No | No | Yes | Yes |
| Sociodemographic controls | No | No | No | Yes |
| Observations | 4173 | 4173 | 4173 | 4173 |
| Individuals | 4173 | 4173 | 4173 | 4173 |

*Note.* Entries are results from Ordered Logistic regressions estimating the determinants of the delayed gratification scale—ranging from 0 (impatient) to 100 (patient)—which has been categorized into quartiles. Odds ratios are reported, with t-statistics based on robust standard errors shown in brackets. Asymmetric anticipatory and reactive emotions are standardized and measures higher ‘disutility’ from anticipating or experiencing income losses relative to ‘utility’ from income gains. We use analytical weights to account for variation in the number of observations per individual when estimating asymmetric anticipatory and reactive emotions. Individuals observed more frequently are given greater weight, reflecting the higher precision of their emotion estimates. Lastly, the parallel lines (proportional odds) assumption—where the effect of each predictor variable on the outcome is consistent across all categories—is met for our measures of asymmetric anticipatory and reactive emotions within the Ordered Logistic regressions. Moreover, the unreported results, from a Partial Proportional Odds Model—which relaxes the parallel lines assumption of the Ordered Logistic model—are quantitively and qualitatively similar. For details about the control variables see the notes for Table B9.

$$*p< .1 **p< .05 ***p< .01$$

**Table B11.** *Predictors of Time Preferences – Logistic Regression*

| Dependent variable: High vs. low delayed gratification | | | | |
| --- | --- | --- | --- | --- |
| Predictors | Regression 1 | Regression 2 | Regression 3 | Regression4 |
| Asymmetric anticipatory emotions | 0.851*** | 0.885*** | 0.882*** | 0.868*** |
|  | [-5.209] | [-3.668] | [-3.662] | [-4.139] |
| Asymmetric reactive emotions |  | 0.896*** | 0.899*** | 0.924** |
|  |  | [-3.027] | [-2.796] | [-2.066] |
|  |  |  |  |  |
| Exogeneous controls | Yes | Yes | Yes | Yes |
| Trait optimism/anxiety/state GHQ | No | No | Yes | Yes |
| Sociodemographic controls | No | No | No | Yes |
| Observations | 4173 | 4173 | 4173 | 4173 |
| Individuals | 4173 | 4173 | 4173 | 4173 |

*Note.* Entries are results from Logistic regressions estimating the determinants of the delayed gratification scale—ranging from 0 (impatient) to 100 (patient)—which has been dichotomized into high vs. low patience (equal to one if the respondent is in the highest quartile of the patience distribution, and zero otherwise). Odds ratios are reported, with t-statistics based on robust standard errors shown in brackets. Asymmetric anticipatory and reactive emotions are standardized and measures higher ‘disutility’ from anticipating or experiencing income losses relative to ‘utility’ from income gains. For details about the control variables see the notes for Table B9.

$$*p< .1 **p< .05 ***p< .01$$

**Table B12.** *Predictors of Time Preferences – Winsorizing Asymmetric Emotions*

| Dependent variable: Delayed gratification scale | | | | |
| --- | --- | --- | --- | --- |
| Predictors | Regression 1 | Regression 2 | Regression 3 | Regression4 |
| Asymmetric anticipatory emotions | -1.221*** | -0.869*** | -0.812*** | -0.935*** |
|  | [-6.287] | [-4.133] | [-3.891] | [-4.569] |
| Asymmetric reactive emotions |  | -0.931*** | -0.837*** | -0.605*** |
|  |  | [-4.235] | [-3.785] | [-2.798] |
|  |  |  |  |  |
| Exogeneous controls | Yes | Yes | Yes | Yes |
| Trait optimism/anxiety/state GHQ | No | No | Yes | Yes |
| Sociodemographic controls | No | No | No | Yes |
| R-squared | 0.017 | 0.022 | 0.040 | 0.091 |
| Observations | 4173 | 4173 | 4173 | 4173 |
| Individuals | 4173 | 4173 | 4173 | 4173 |

*Note.* Entries are results from OLS regressions estimating the determinants of the delayed gratification scale—ranging from 0 (impatient) to 100 (patient). Unadjusted coefficients are reported, with t-statistics based on robust standard errors shown in brackets. Asymmetric anticipatory and reactive emotions are standardized and measures higher ‘disutility’ from anticipating or experiencing income losses relative to ‘utility’ from income gains. We winsorize the asymmetric anticipatory and reactive emotions at the 5^th^ and 95^th^ percentiles to mitigate the potential impact of outliers. We use analytical weights to account for variation in the number of observations per individual when estimating asymmetric anticipatory and reactive emotions. Individuals observed more frequently are given greater weight, reflecting the higher precision of their emotion estimates. For details about the control variables see the notes for Table B9.

$$*p< .1 **p< .05 ***p< .01$$

**Table B13.** *Predictors of Time Preferences – The Impact of Dread and Savoring*

| Dependent variable: Delayed gratification scale | | | | |
| --- | --- | --- | --- | --- |
| Predictors | Regression 1 | Regression 2 | Regression 3 | Regression4 |
| Dread | -1.701*** | -1.331*** | -1.247*** | -1.398*** |
|  | [-6.801] | [-4.939] | [-4.628] | [-5.346] |
| Savoring | 1.011*** | 0.789*** | 0.769*** | 0.863*** |
|  | [4.212] | [3.134] | [3.100] | [3.565] |
| Asymmetric reactive emotions |  | -0.635*** | -0.580*** | -0.377** |
|  |  | [-3.366] | [-3.011] | [-2.003] |
| $\left\vert bDread \right\vert-\vert bSavoring\vert$ | 0.690*** | 0.542*** | 0.478*** | 0.536*** |
|  | [3.890] | [3.040] | [2.690] | [3.090] |
|  |  |  |  |  |
| Exogeneous controls | Yes | Yes | Yes | Yes |
| Trait optimism/anxiety/state GHQ | No | No | Yes | Yes |
| Sociodemographic controls | No | No | No | Yes |
| R-squared | 0.020 | 0.023 | 0.040 | 0.093 |
| Observations | 4173 | 4173 | 4173 | 4173 |
| Individuals | 4173 | 4173 | 4173 | 4173 |

*Note.* Entries are results from OLS regressions estimating the determinants of the delayed gratification scale—ranging from 0 (impatient) to 100 (patient). Unadjusted coefficients are reported, with t-statistics based on robust standard errors shown in brackets. Dread and savoring are both standardized and measure the ‘disutility’ from anticipating income losses and the ‘utility’ from anticipating income gains, respectively. Asymmetric reactive emotions are standardized and measures higher ‘disutility’ from experiencing income losses relative to ‘utility’ from experiencing income gains. We use analytical weights to account for variation in the number of observations per individual when estimating asymmetric anticipatory and reactive emotions. Individuals observed more frequently are given greater weight, reflecting the higher precision of their emotion estimates. For details about the control variables see the notes for Table B9.

$$*p< .1 **p< .05 ***p< .01$$

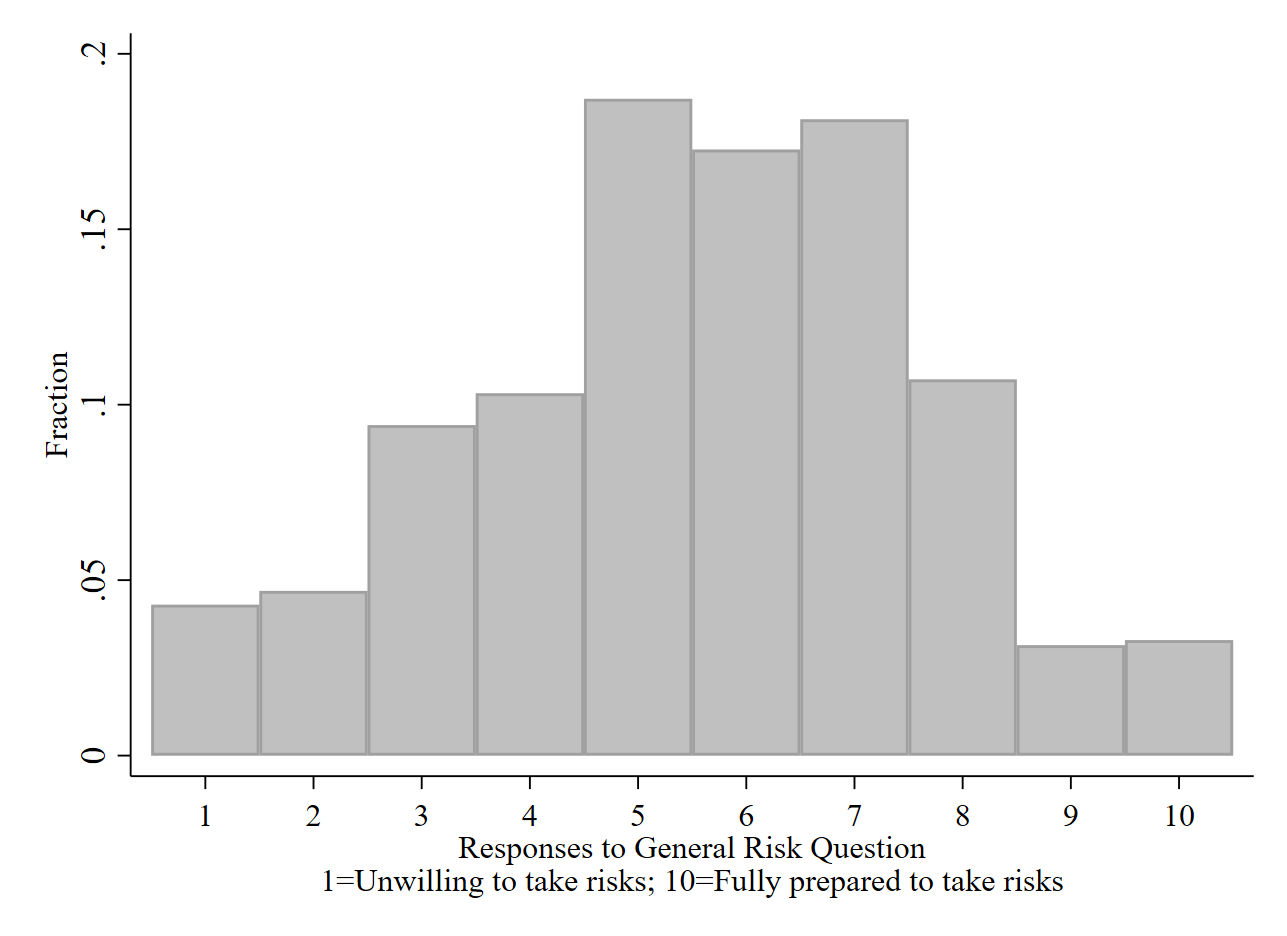


**Figure B1.** Histogram of responses to the General Risk Question ($n$ = 6,839).


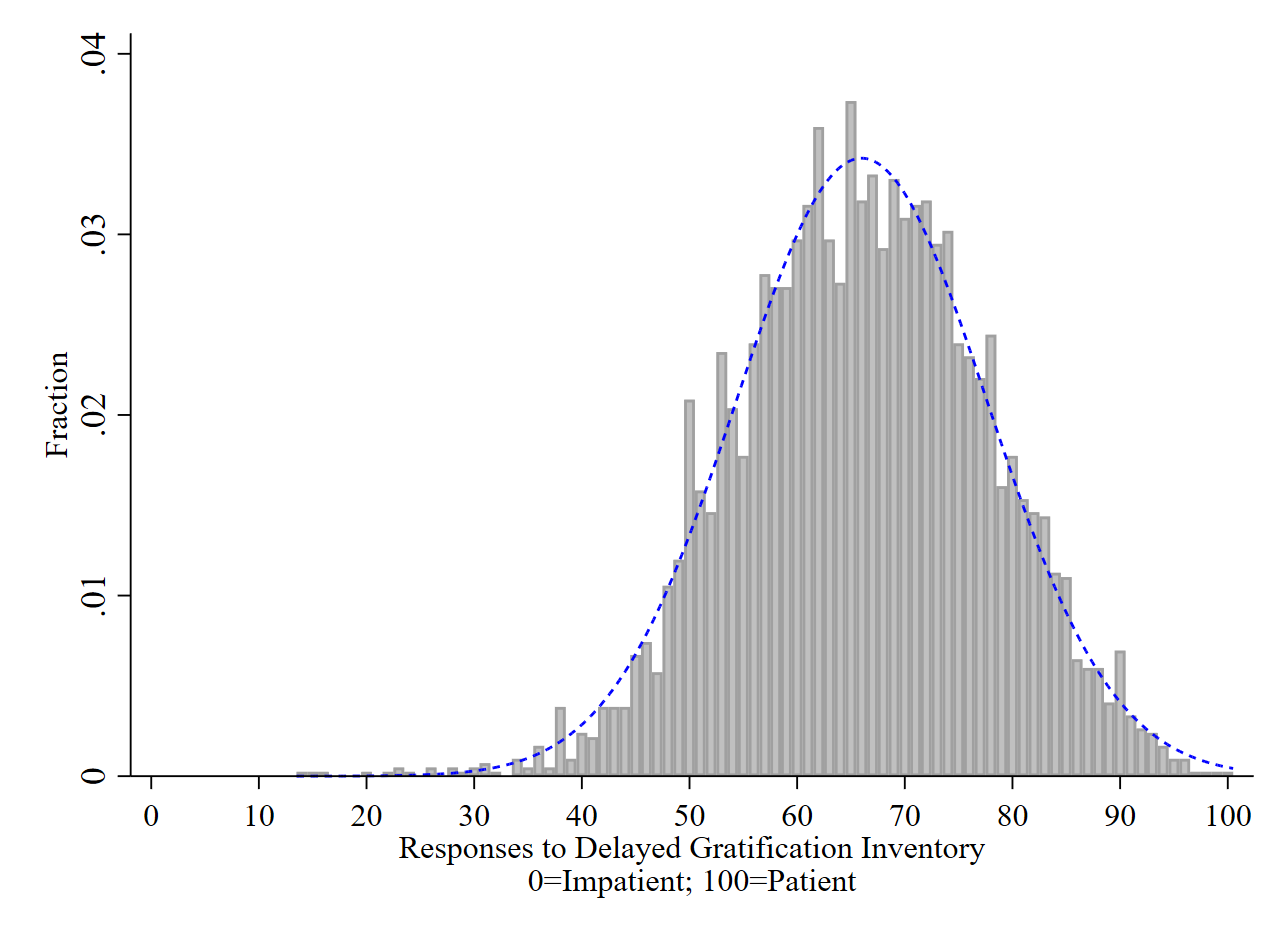


**Figure B2.** Histogram of responses to the Delayed Gratification Inventory ($n$ = 4,173).

*Note.* A normal density is also plotted, with the same mean and variance as the data.


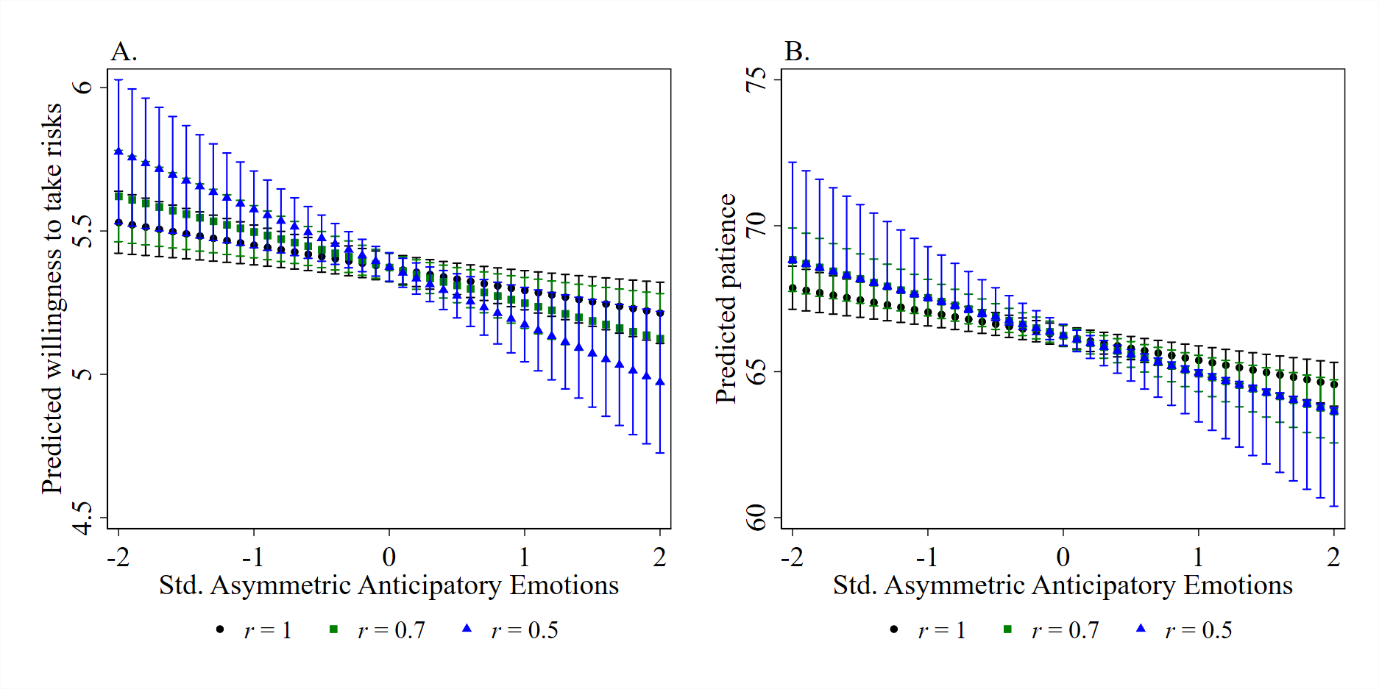


**Figure B3.** The predicted relationship between asymmetric anticipatory emotions and economic preferences for different levels of reliability in asymmetric anticipatory emotions.

*Note.* The predicted regression lines are for different levels of reliability, $r$, in asymmetric anticipatory emotions. Error bars are 95% confidence intervals.
